# Identification of a novel defined inflammation-related long noncoding RNA signature contributes to predicting prognosis and distinction between the cold and hot tumors in bladder cancer

**DOI:** 10.3389/fonc.2023.972558

**Published:** 2023-03-29

**Authors:** Xi Xiong, Chen Chen, Xinxin Li, Jun Yang, Wei Zhang, Xiong Wang, Hong Zhang, Min Peng, Lili Li, Pengcheng Luo

**Affiliations:** ^1^ Department of Urology, Wuhan Third Hospital School of Medicine, Wuhan University of Science Technology, Wuhan, China; ^2^ Department of Urology, Wuhan Third Hospital and Tongren Hospital of Wuhan University, Wuhan, China; ^3^ Department of Urology, Wuhan Third Hospital, Wuhan, China; ^4^ Department of Pharmacy, Wuhan Third Hospital, Wuhan, China; ^5^ Department of Oncology, Renmin Hospital of Wuhan University, Wuhan, China; ^6^ Central Laboratory, Renmin Hospital of Wuhan University, Wuhan, China

**Keywords:** bladder cancer, inflammation, lncRNA, prognosis, immune

## Abstract

**Purpose:**

Bladder cancer (BLCA) is one of the most frequently diagnosed urological malignancies and is the 4th most common cancer in men worldwide. Molecular targets expressed in bladder cancer (BLCA) are usually used for developing targeted drug treatments. However, poor prognosis and poor immunotherapy efficacy remain major challenges for BLCA. Numerous studies have shown that long non-coding RNAs (LncRNAs) play an important role in the development of cancer. However, the role of lncRNAs related to inflammation in BLCA and their prognostic value remain unclear. Therefore, this study is aimed to explore new potential biomarkers that can predict cancer prognosis.

**Methods:**

We downloaded BLCA-related RNA sequencing data from The Cancer Genome Atlas (TCGA) and searched for inflammation-related prognostic long non-coding RNAs (lncRNAs) by univariate Cox (uniCox) regression and co-expression analysis. We used the least absolute shrinkage and selection operator (LASSO) analysis to construct an inflammation-related lncRNA prognosis risk model. Samples were divided into high-risk score (HRS) group and low-risk score (LRS) group based on the median value of risk scores. The independent variable factors were identified by univariate Cox (uni-Cox) and multivariate Cox (multi-Cox) regression analyses, and receiver operating characteristic (ROC) curves were used to compare the role of different factors in predicting outcomes. Nomogram and Calibration Plot were generated by the R package rms to analyze whether the prediction results are correct and show good consistency. Correlation coefficients were calculated by Pearson analysis. The Kaplan-Meier method was used to assess the prognostic value. The expression of 7 lncRNAs related with inflammation was also confirmed by qRT-PCR in BLCA cell lines. Kyoto Encyclopedia of Gene and Genome (KEGG) pathways that were significantly enriched (*P* < 0.05) in each risk group were identified by the GSEA software. The R package pRRophetic was used to predict the IC50 of common chemotherapeutic agents. TIMER, XCELL, QUANTISEQ, MCPCOUNTER, EPIC and CIBERSORT were applied to quantify the relative proportions of infiltrating immune cells. We also used package ggpubr to evaluate TME scores and immune checkpoint activation in LRS and HRS populations. R package GSEABase was used to analyze the activity of immune cells or immune function. Different clusters of principal component analysis (PCA), t-distribution random neighborhood embedding (t-SNE), and Kaplan-Meier survival were analyzed using R package Rtsne’s. The R package ConsensesClusterPlus was used to class the inflammation-related lncRNAs.

**Results:**

In this study, a model containing 7 inflammation-related lncRNAs was constructed. The calibration plot of the model was consistent with the prognosis prediction outcomes. The 1-, 3-, and 5-year ROC curve (AUC) were 0.699, 0.689, and 0.699, respectively. High-risk patients were enriched in lncRNAs related with tumor invasion and immunity, and had higher levels of immune cell infiltration and immune checkpoint activation. Hot tumors and cold tumors were effectively distinguished by clusters 2 and 3 and cluster 1, respectively, which indicated that hot tumors are more susceptible to immunotherapy.

**Conclusion:**

Our study showed that inflammation-related LncRNAs are closely related with BLCA, and inflammation-related lncRNA can accurately predict patient prognosis and effectively differentiate between hot and cold tumors, thus improving individualized immunotherapy for BLCA patients. Therefore, this study provides an effective predictive model and a new therapeutic target for the prognosis and clinical treatment of BLCA, thus facilitating the development of individualized tumor therapy.

## 1 Introduction

Bladder cancer (BLCA) is a common cancer with high morbidity and mortality rates worldwide. Most BLCA patients are often diagnosed in the late stages and lack effective treatments (1, 2). There were about 81,400 new BLCA cases and 17,980 BLCA deaths in the United States in 2020 alone. Due to the poor selectivity and side effects of traditional treatments, new treatment strategies are urgently needed for BCLA (3). Immunotherapy is increasingly used in the treatment of malignant tumors, particularly advanced BLCA. Although immunotherapy with immune checkpoint inhibitors (ICIs) can greatly increase the survival rate and duration of response, only a few patients respond to ICIs (4). Therefore, finding an effective immunotherapy for BCLA patients remains a key direction of research.

Previous studies have shown that many malignant tumors are caused by infection, which along with inflammation accounts for 25% of the causes of cancer (5, 6). Persistent infection during chronic inflammation can cause cell DNA damage and tissue damage and repair, leading to genomic changes and hence tumor development. Chronic inflammation is characterized by persistent neutrophil infiltration, which is also a feature of the tumor microenvironment (TME). Neutrophils can promote tumorigenesis by inhibiting adaptive immune responses in the TME (7). It has been reported that inflammation is the initiating factor for TME. Macrophages are important components of tumor immune cells and can promote tumorigenesis and tumor development (8). M1 macrophages promote type I immune response by secreting pro-inflammatory cytokines (9, 10), whereas M2 macrophages promote tumor progression by producing T helper 2 (Th2) cytokines (11, 12). Based on these findings, we hypothesize that inflammation-related genes may be potential molecular targets for BLCA treatment.

The role of long non-coding RNAs (lncRNAs) in cancer progression has been increasingly investigated in recent years. LncRNAs are > 200 bp in length and widely distributed throughout the genome. They play a key role in diverse biological and physiological processes, including epigenetic control, cell cycle regulation, and cell differentiation (13). LncRNAs have also been shown to be involved in inflammation-related diseases (14). The inflammatory TME is the key to most tumors progressing from controllable inflammation to uncontrollable inflammation. Therefore, inflammation-related lncRNAs may play a pivotal role in the transition to inflammatory TME during tumor development (15–17). According to research, LncRNA-PDPK2P promotes hepatocellular carcinoma (HCC) progression *via* the PDK1/Akt/Casepase-3 signaling pathway, and PDPK2P expression can be used as a prognostic marker for HCC (18). However, the role of inflammation-related lncRNAs in BCLA treatment is yet to be examined. Here, we investigated the effectiveness of inflammation-related lncRNAs in classifying cold and hot tumors to improve the efficacy of immunotherapy and provide potential biomarkers for the selection of immunotherapy in BLCA patients (19).

## 2 Materials and methods

### 2.1 Data collection

**Figure 1:**
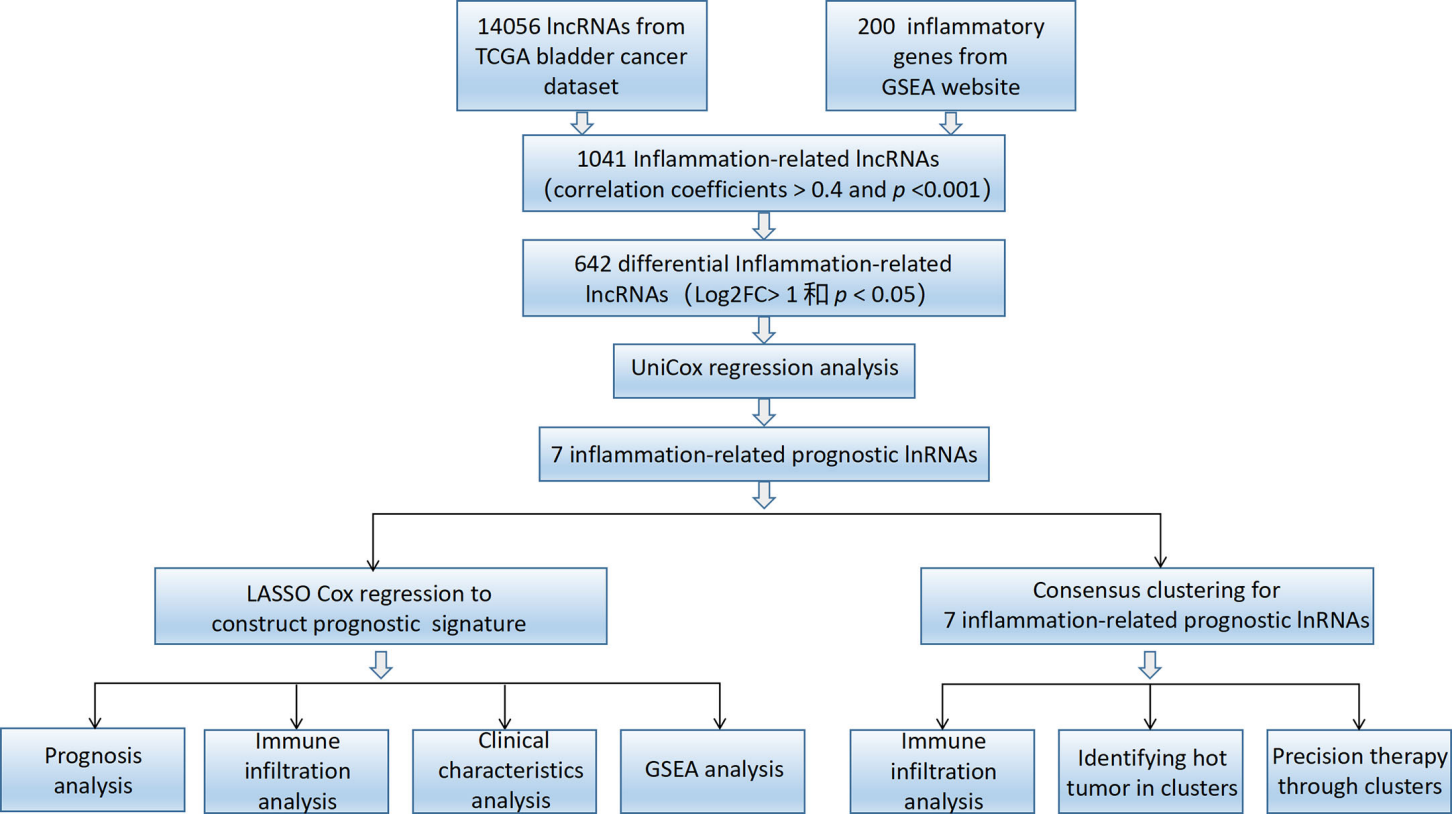
Study workflow.

A workflow for this study is summarized in **Figure 1**. RNA-seq data and clinical characteristics were acquired from the TCGA (https://portal.gdc.cancer.gov/), including 19 normal bladder samples and 414 BLCA samples. Perl was used to combine the individual RNA-seq data by sample ID. A total of 14,056 lncRNAs were identified in the BLCA cohort. Patients with short or missing overall survival (OS; <30 days) were excluded.

### 2.2 Acquisition of inflammation-related lncRNAs

A total of 200 inflammation-related genes were downloaded from the Molecular Signatures Database (http://www.gsea-msigdb.org/gsea/index.jsp) (**Supplementary Table 2**). The correlation between the 14056 lncRNAs and the 200 inflammation-related genes in BLCA was determined using the Spearman approach (20). LncRNAs with a correlation coefficient of > 0.4 and *P* < 0.001 were defined as inflammation-related lncRNAs. Differentially expressed inflammation-related lncRNAs were identified by |log|FC| > 1 and *P* < 0.05 using the R package Limma.

### 2.3 Prognostic model construction and LASSO regression analysis

To determine the stability and accuracy of the prognosis, we analyzed the 1,041 inflammation-related lncRNAs by uniCox regression and removed overfitting variables using LASSO regression. A PRS model was constructed to accurately predict overall survival rate (OS) of BLCA samples. The ideal values for the penalty parameters were identified after 10 cross-validations to build the inflammation-related lncRNAs-based prognostic model (21). The 1-, 3-, and 5-year receiver operating characteristic (ROC) curves of the model were constructed. The risk score was calculated by: Risk score $\sum_{i=1}^{n}\text{coef(i)}\times \text{expr(i)}$ , where expr (i) is the relative mRNA expression of the gene in every patient i, and coef (i) is the LASSO coefficient of the gene in every patient i. The median risk score was used to classify samples into low-risk score (LRS) and high-risk score (HRS) groups. We obtained 400 patients with pertinent clinical information and randomly divided them at 1:1 into the training and test groups using by “caret” package.

### 2.4 Independence factor analysis

Independent factors, including risk scores and clinical parameters, were analyzed by uniCox and multiCox regression analyses. ROC analysis was used to determine the predictive accuracy of each factor.

### 2.5 Construction of nomogram and calibration plot

Nomograms for the 1-, 3-, and 5-year OS rates were generated by the “rms” package based on the risk score and clinical parameters. A calibration plot was created to measure the consistency between the predicted outcome and the actual survival rate.

### 2.6 Functional enrichment analysis

Kyoto Encyclopedia of Gene and Genome (KEGG) pathways that were significantly enriched (*P* < 0.05) in each risk group were identified by the GSEA software (https://www.gsea-msigdb.org/gsea/login.jsp).

### 2.7 TME and immune checkpoints

The enrichment of immune cytokines in the HRS group were evaluated based on the GSEA results. To evaluate immune cell infiltration, correlations between risk scores and immune checkpoint molecule expression were determined by TIMER, EPIC, MCPcounter, QUANTISEQ, CIBERSORT, XCELL, and CIBERSORT. TME variations in each risk group were analyzed by Limma package and presented in a bubble chart. TME score and immune checkpoint activity were compared between the LRS and HRS groups by the “ggpubr” package.

### 2.8 Prediction of potential compounds for BLCA treatment

IC_50_ values of compounds were obtained from the Genomics of Drug Sensitivity in Cancer (GDSC) website (https://www.cancerrxgene.org/) to identify candidate drugs for BLCA treatment. Chemicals that may be used for BCLA treatment were predicted using the R packages pRRophetic, limma, ggpub, and ggplot2 (22).

### 2.9 Consensus clustering by seven inflammation-related prognostic lncRNAs

To study BLCA response to immunotherapy, BLCA patients were divided into subgroups by the “ConsensesClusterPlus” package, and the immunotherapy responses in these subgroups were determined based on the lncRNA expression levels. Principal component analysis (PCA), Kaplan–Meier estimation, and t-distributed stochastic neighbor embedding (t-SNE) were performed by the “Rtsne” package, and immunoassay and drug sensitivity analyses were performed by “Limma”, “scales”, and “pRRophetic” packages.

### 2.10 Cell culture

Sv-huc-1, T24, and 5637 cell lines were purchased from IMMOCELL (Xiamen, Fujian, China) and respectively cultured in DMEM-F12K medium, McCoy’s 5A medium, and RPMI-1640 medium containing 1% streptomycin-penicillin and 10% FBS (Gibco) at 37°C.

### 2.11 RNA extraction and qRT-PCR

Total RNA extraction was performed on the three cell lines using the RNA Isolation Kit (R6934-01, Omega Bio-Tek, USA) as per the manufacturer’s protocol. Total RNA concentration and purity were determined by absorbance readings at 260 nm with a spectrophotometer (Quawell UV Spectrophotometer Q3000). RNA was reversely transcribed into cDNA using the TOYOBO ReverTra Ace qPCR RT kit, and amplified by real-time PCR using the Bio-Rad CFX Manager system. Expression levels of AC068196.1, LNCAROD, MAP3K14-AS1, AC021321.1, LINC02256, NR2F1-AS1, and KCNQ1OT1 were calculated by the 2^−ΔΔCq^ method (23). Primers are shown in **Supplementary Table 1**.

### 2.12 Statistic analysis

Spearman correlation analysis was employed to calculate correlation coefficients. Using the Kaplan–Meier method, the Cox regression model, and log-rank test, the prognostic value was evaluated. The Wilcoxon rank-sum test was utilized to compare the two groups. Three or more groups were compared by the Kruskal-Wallis test. Two-tailed P < 0.05 indicated significant difference.

## 3 Results

### 3.1 Identification of inflammation-related lncRNAs in BLCA

**Figure 2:**
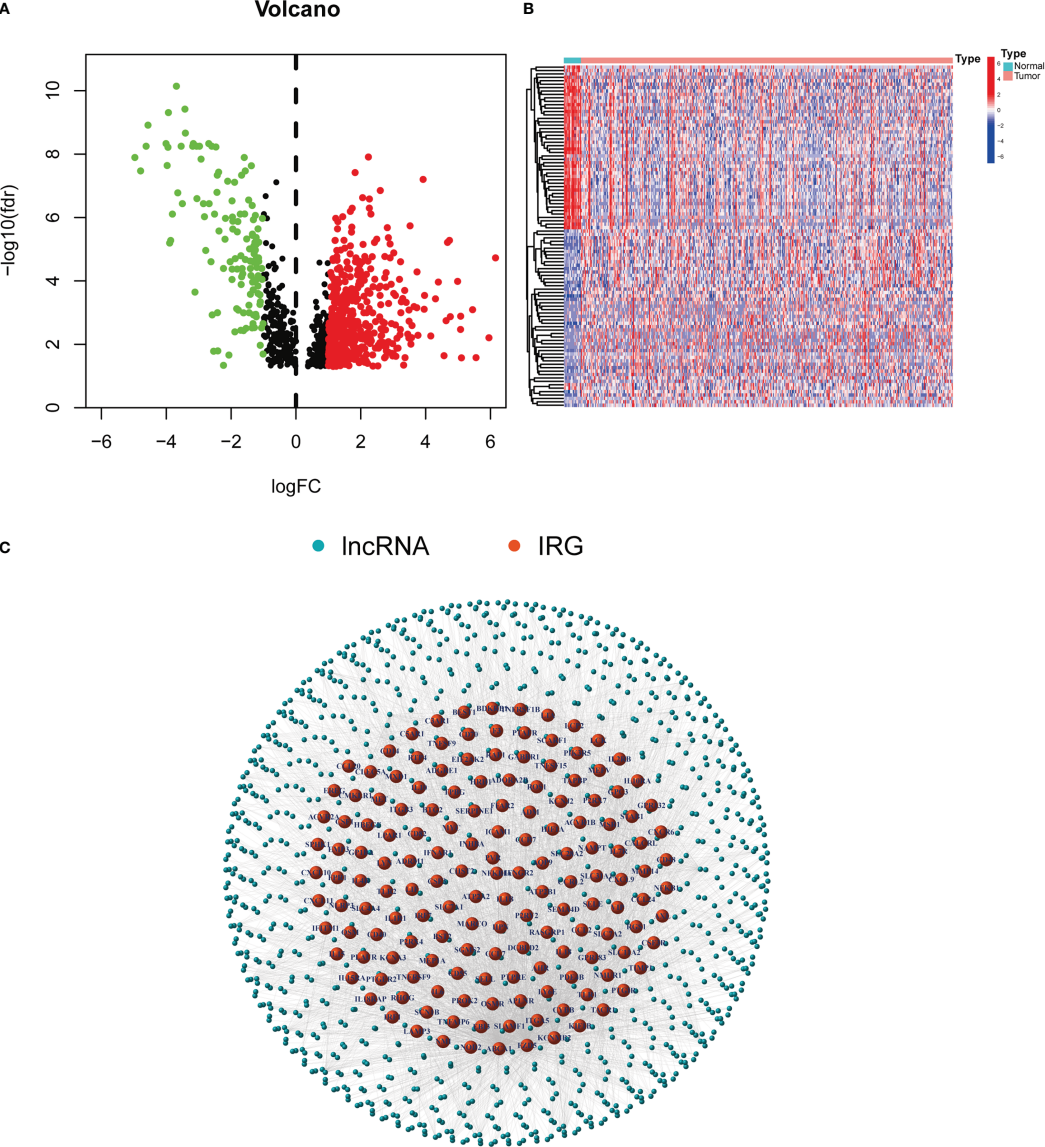
Inflammation-related lncRNAs in BLCA patients. **(A)** Volcano plot of 642 differentially expressed lncRNAs. **(B)** Heatmap of the expression of 642 inflammation-related lnRNAs in normal samples and tumor samples. **(C)** Network between 200 inflammation-related genes and 642 lncRNAs.

A workflow of this study is shown in **Figure 1**. The RNA-seq data of 433 samples, including 19 normal samples and 414 BCLA samples, and their corresponding 412 clinical files were analyzed. A total of 1,041 inflammation-related lncRNAs were identified through correlation analysis of the expression of 14,056 lncRNAs and the mRNA expression of 200 inflammation-related genes. 642 differentially expressed inflammation-related lncRNAs, including 515 upregulated and 127 downregulated lncRNAs, were identified (**Figure 2A**). The heatmap results revealed that BLCA patients could be clearly divided into two distinct groups (**Figure 2B**). The correlations between the 200 inflammation-related genes and 642 lncRNAs are shown in **Figure 2C**; **Supplementary Table 3**.

### 3.2 Prognostic signature construction and validation

**Figure 3:**
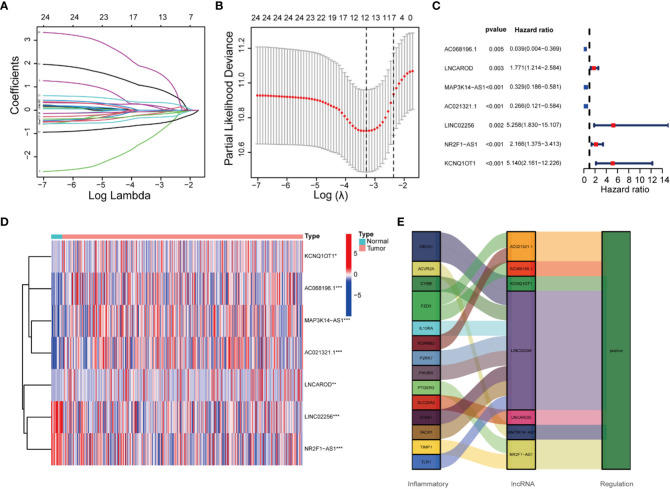
Construction of the 7 inflammation-related lncRNAs model.Cox regression analysis of inflammation-related lncRNAs in BLCA. **(A)** 10-fold cross-validation in the LASSO analysis. **(B)** LASSO coefficient profile of the 7 inflammation-related lncRNAs. **(C)** Univariate Cox regression analysis. **(D)** Heatmap of the expression profiles of 7 lncRNAs. **(E)** The Sankey diagram of inflammation genes and lncRNAs.

**Figure 4:**
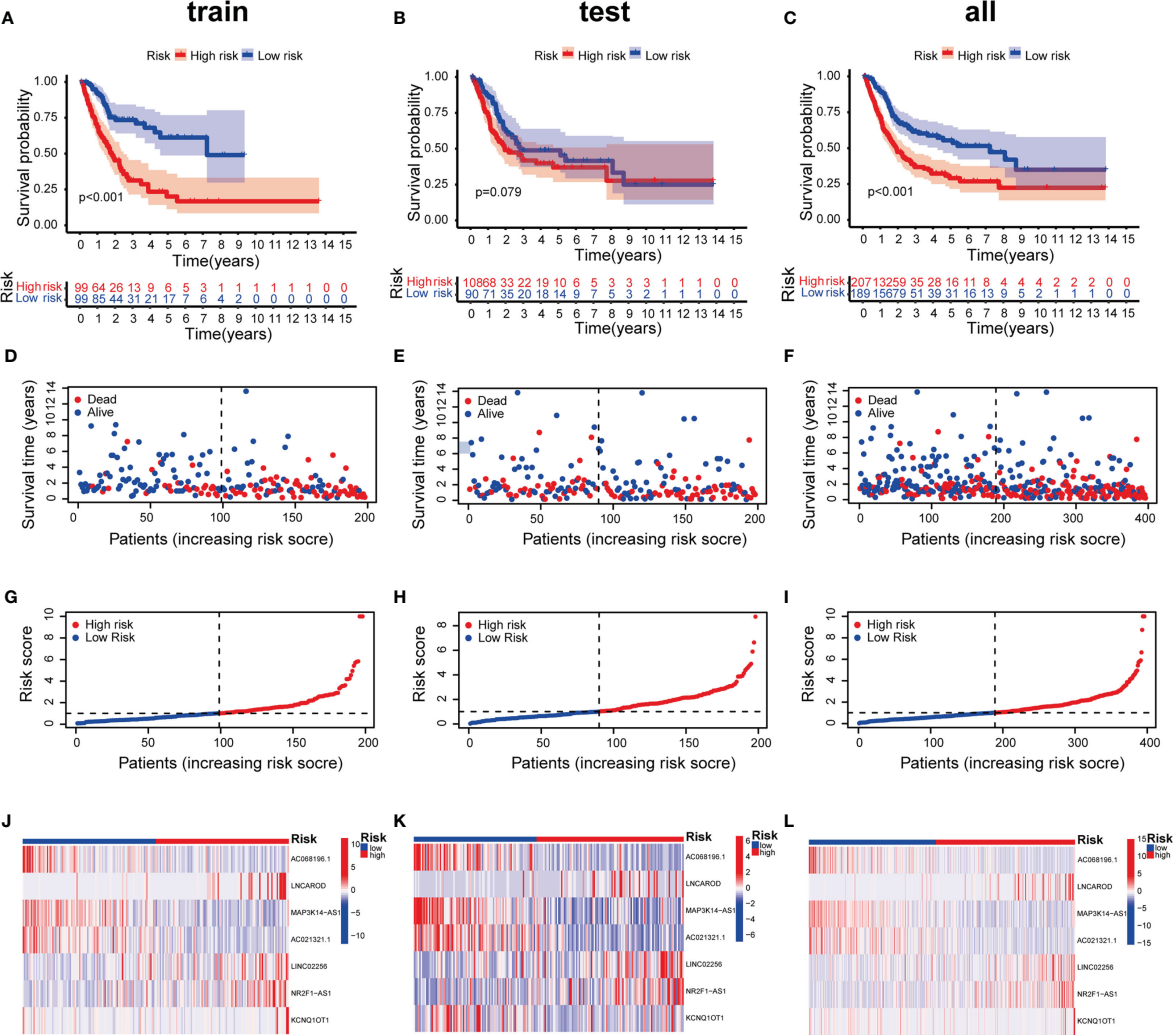
Prognosis value of the 7 inflammation-related lncRNAs model in the train, test, and entire sets. **(A–C)** Kaplan–Meier survival curves of patients in the train, test, and entire datasets, respectively. **(D-F)** Survival time and status in the train, test, and entire datasets, respectively. **(G–I)** Inflammation-related lncRNAs model based on risk scores of the train, test, and entire datasets, respectively. **(J–L)** Heatmaps of the expression of the 7 lncRNAs in the train, test, and entire sets, respectively.

To prevent overfitting of the prognostic signature, We performed lasso regression on these lncRNAs and extracted 7 lncRNAs related to inflammation in BLCA, when the first-order value of Log(λ) was the least probable deviation (**Figures 3A, B**). We used the following formula to calculate the risk score: AC068196.1 × (-2.68949664829451) + LNCAROD × (0. 313548802975356) + MAP3K14-AS1 × (-0.936638242135287) + AC021321.1 × (-0.746810149653322) + LINC02256 × (1.412877497541) + NR2F1-AS1 × (0.563351996798577) + KCNQ1OT1 × (2.09002192071773) (24). 7 inflammation-related lncRNAs were markedly correlated with OS as determined by uniCox regression (all *P* < 0.05) and made a heatmap (**Figures 3C, D**). In addition, The Sankey diagram revealed that 7 lncRNAs were upregulated (**Figure 3E**). Furthermore, using the risk score formula, the distribution of risk scores, survival status, survival time, and the related expression criteria of these lncRNAs were compared between the LRS and HRS groups of patients in the train, test, and entire sets, which revealed that the patients in the high-risk group had low overall survival rate and worse prognosis (**Figure 4**).

### 3.3 LncRNA expression in BCLA cell lines

**Figure 5:**
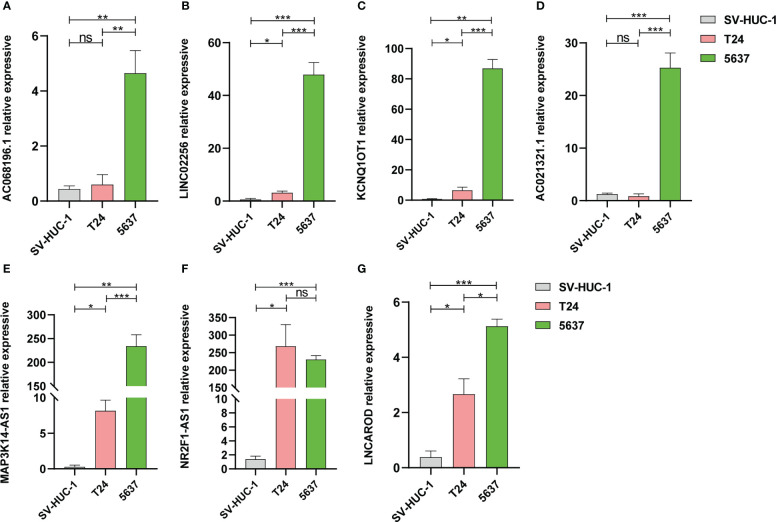
The mRNA expression levels of 7 lncRNAs in two bladder cancer cell lines by qRT-PCR. **(A)** AC068196.1; **(B)** LINC02256; **(C)** KCNQ1OT1; **(D)** AC021321.1; **(E)** MAP3K14-AS1; **(F)** NR2F1-AS1; **(G)** LNCAROD. *p < 0.05, **p < 0.01, and ***p < 0.001. ns, not statistically significant.

Furthermore, we validated expression of the 7 inflammation-related lncRNAs in the normal bladder cell (SV-HUC-1), and BLCA cells (T24 and 5637) by qRT-PCR. The expression level of AC021321.1, AC068196.1 showed no difference between T24 cell line and SV-HUC-1. The expression level of LNCAROD, LINC02256, NR2F1-AS1, MAP3K14-AS1, and KCNQ1OT were significantly higher in the BLCA cell lines than in SV-HUC-1 cells (**Figure 5**).

### 3.4 Cox analysis and nomogram construction

**Figure 6:**
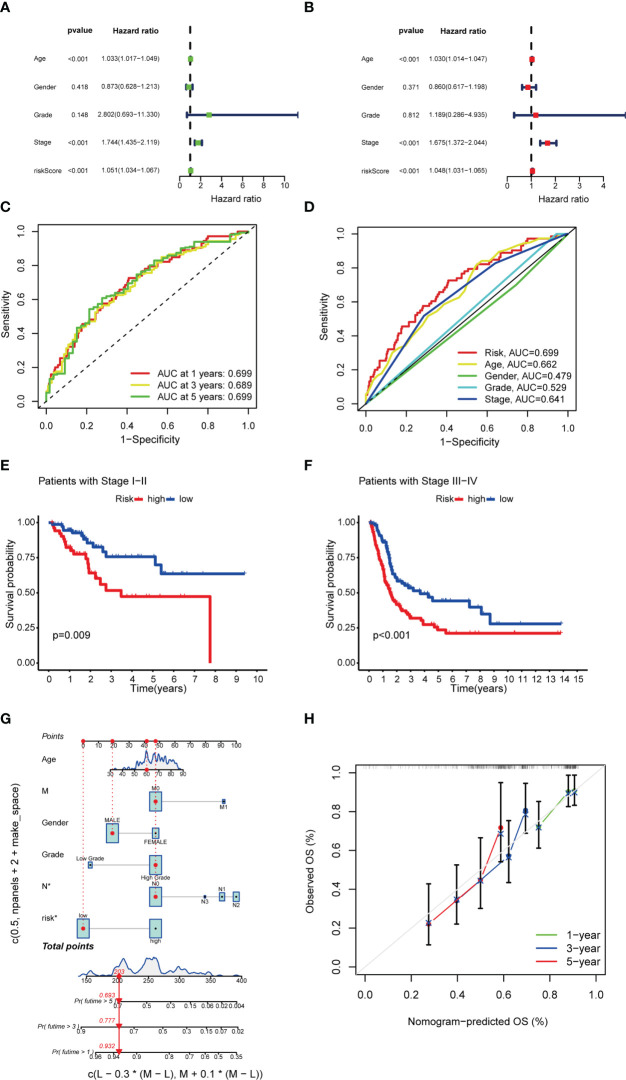
Univariate Cox and multivariate Cox regression analyses and nomogram construction. **(A, B)** Univariate Cox **(A)** and multivariate Cox **(B)** analyses of clinical factors and risk score for prediction of overall survival. **(C, D)** 1-, 3-, and 5-year ROC curves of risk score and clinical characteristics. **(E, F)** Kaplan–Meier survival curves of patients in the stage I-II and stage III-IV groups, respectively. **(G)** 1-, 3-, and 5-year overall survival predicted by the nomogram integrating risk score, age, and tumor staging. **(H)** Calibration curves for 1-, 3-, and 5-year overall survival.

UniCox regression revealed significant differences in risk scores (HR=1.051 and 95%CI [1.034-1.067], *P* < 0.001), age (HR=1.030, 95%CI [1.014-1.047], *P* < 0.001) and stage (HR=1.675, 95%CI [1.372-2. 044]; *P* < 0.001) (**Figure 6A**). MultiCox regression also showed significant differences in risk scores (HR=1.048 and 95%CI [1.031-1. 065], *P* < 0.001), age (HR=1.030 and 95%CI [1.014-1.047], *P* < 0.001) and stage (HR=1.675 and 95%CI [1.372-2.044], *P* < 0.001) (**Figure 6B**), indicating that risk score, age, and stage were three independent prognostic factors for BCLA. Furthermore, the ROC AUC of the 1-, 3-, and 5-year OS rates was 0.699, 0.689, and 0.699, respectively, which demonstrated that the constructed model accurately predicted the OS rate of BLCA patients (**Figure 6C**). By combining the results of the constructed model with clinical traits, it was revealed that the risk score has a significantly higher AUC than other clinical traits, indicating that risk score was superior to other clinical traits in OS prediction for BLCA patients (**Figure 6D**). OS rates of stages I-II and stages III-IV BLCA patients were significantly lower in the HRS group than in the LRS group (**Figures 6E, F**). It indicated that patients with different staging had significantly lower survival rates in the HRS group than in the LRS group. A nomogram was further constructed to predict the 1-, 3-, and 5- year OS of BCLA patients (**Figure 6G**), We also utilized the 1-, 3-, and 5-year calibration plots to attest that the nomogram had a good concordance with the prediction of 1-,3-, and 5-year OS (**Figure 6H**).

### 3.5 GSEA

**Figure 7:**
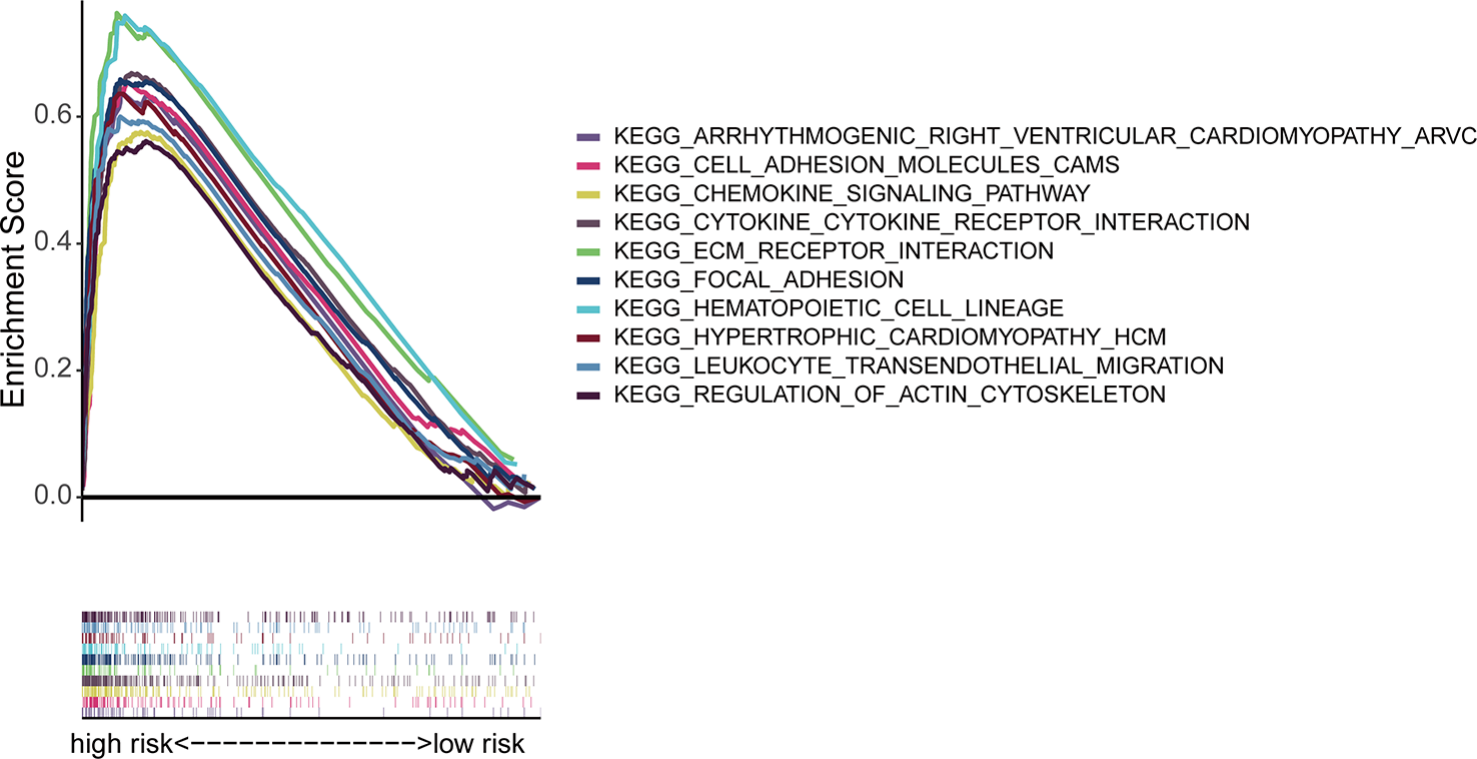
Enrichment of immune cytokines in the top 10 pathways in the high-risk group.

In order to study the differences in biological functions between risk groups, we used GSEA software to explore HRS groups across the entire set KEGG pathway. The GSEA of the HRS group showed that the majority of the top 10 enriched KEGG pathways were related with tumor immunity and invasion, including “chemokine signal pathway” and “leukocyte transendothelial migration” (**Figure 7**; **Supplementary Figure 1**). Therefore, we attempted to perform immune analysis in the model.

### 3.6 Immune cell and drug sensitivity analyses

**Figure 8:**
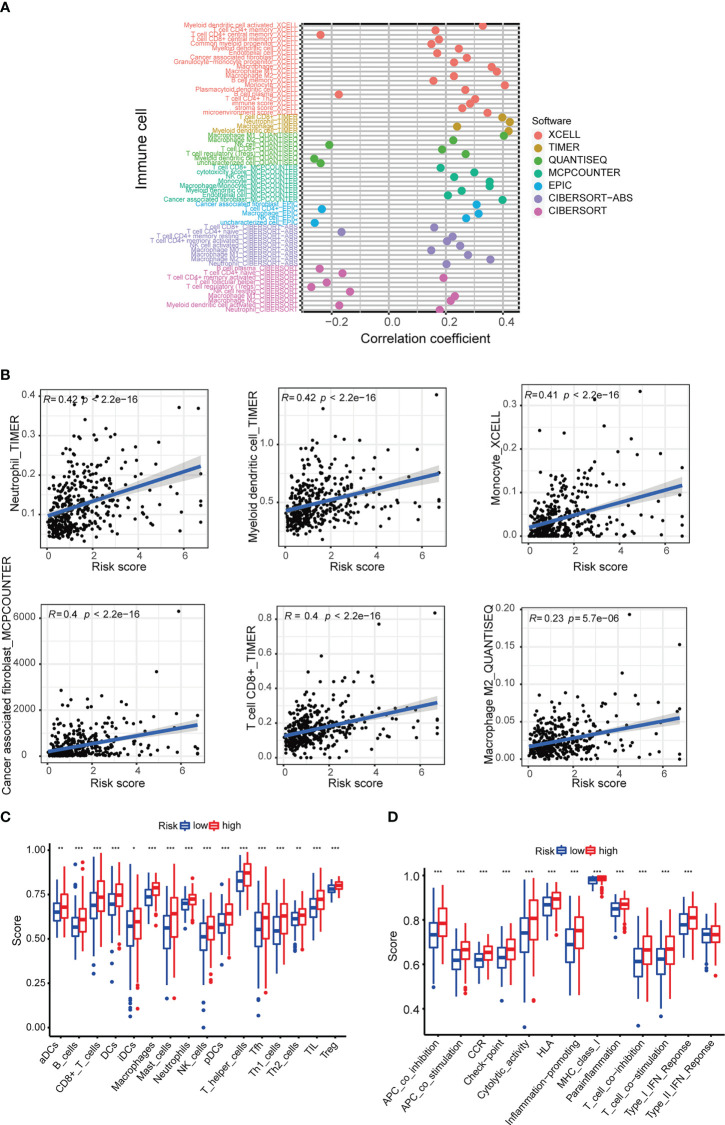
Investigation of tumor immune factors. **(A)** The immune cell bubble of risk groups. **(B)** The correlation between risk score and immune cells. **(C, D)** Relative frequency of immune cells and immunological activities assessed using ssGSE. *p < 0.05, **p < 0.01, and ***p < 0.001.

**Figure 9:**
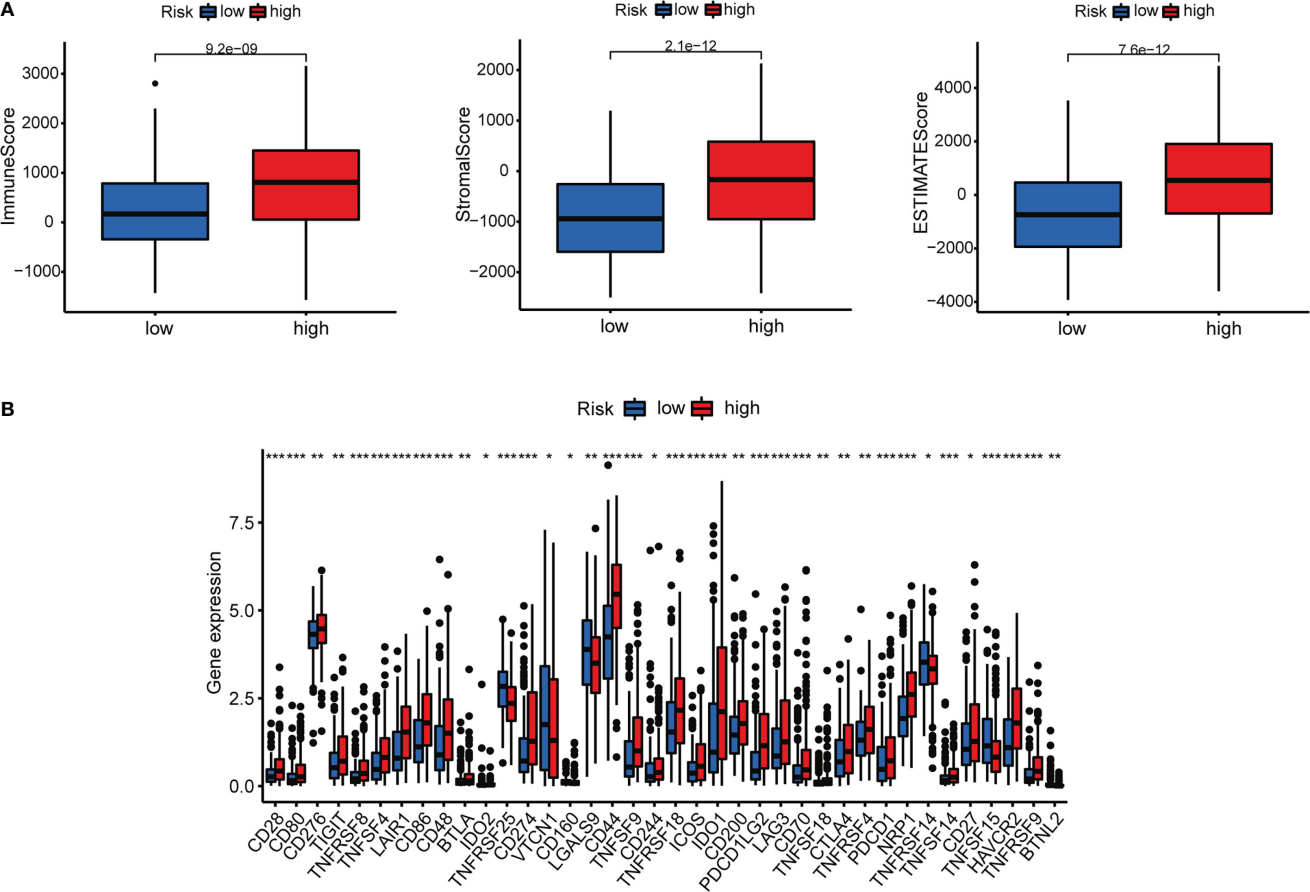
Investigation of tumor immunotherapy. **(A)** Comparison of immune scores between low-risk and high-risk groups. **(B)** Expression of 18 immune checkpoints in different risk groups. *p<0.05, **p<0.01, and ***p<0.001.

**Figure 10:**
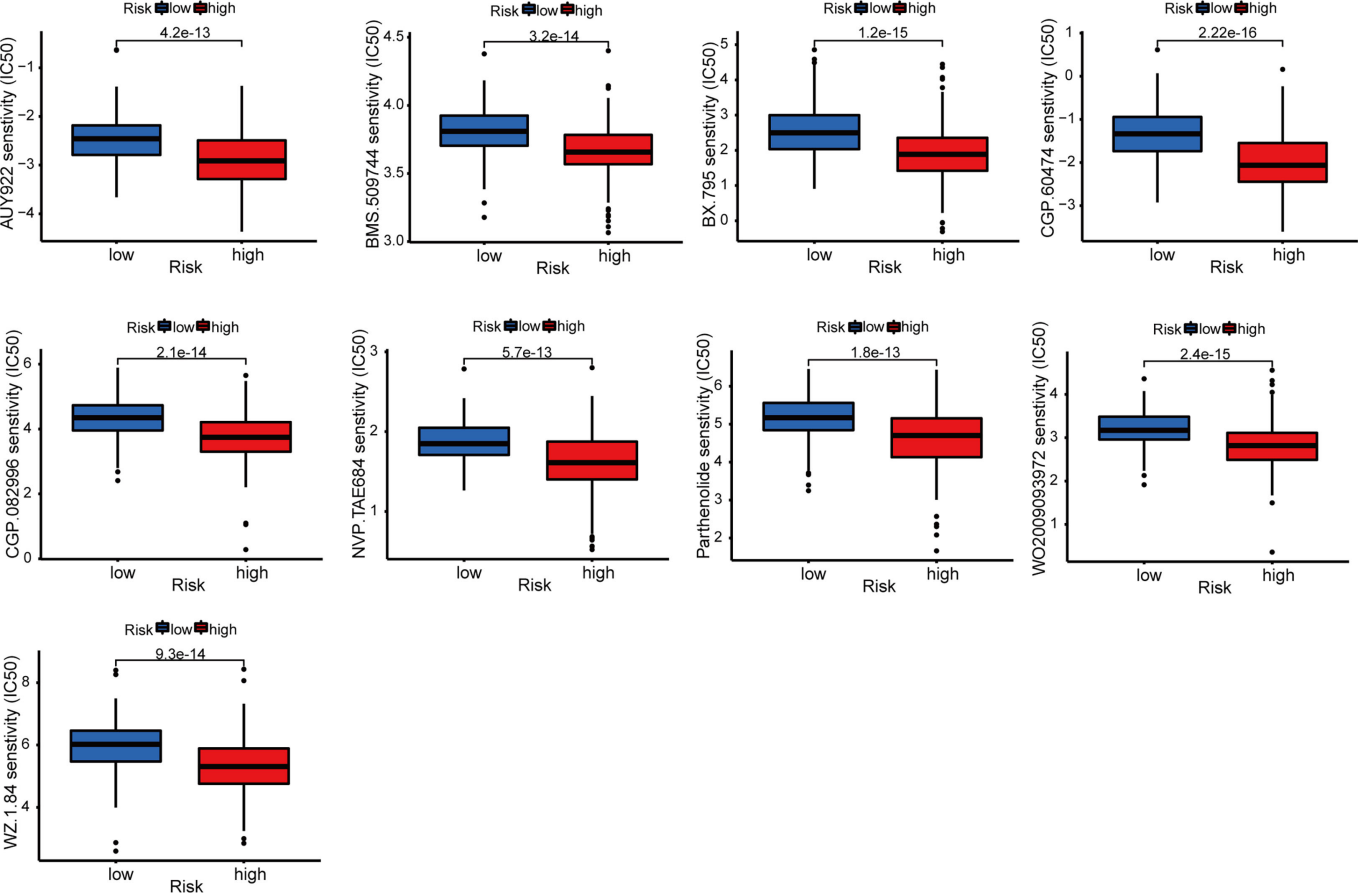
Immunotherapy response of risk groups. The IC_50_ values of 9 immunotherapeutic, chemical, or targeted agents used in the risk groups.

Immune cell bubble plots showed that more immune cells are related with HRS populations on different databases (**Figure 8A**). The correlation of immune cells calculated on different platforms indicated that the risk score was positively correlated with Neutrophil TIMER, Myeloid dendritic cell_TIMER, Monocyte_XCELL, Macrophage M1_QUANTISEQ, T cell CD8+_TIMER, and Macrophage M1_XCELL, Macrophage_XCELL (*P* < 0.05) (**Figure 8B**; **Supplementary Table 4**). ssGSEA revealed higher relative frequency of immune cells and better immune functions in the HRS group than in the LRS group (**Figures 8C, D**). The HRS group had higher immune cell scores, stromal cell scores, and ESTIMATE scores, indicating greater immune cell infiltration (**Figure 9A**). It was also found that both immune cell infiltration and ICI activation were higher in the HRS group (**Figure 9B**) (25). We can select appropriate checkpoint inhibitors for BLCA patients who can be grouped according to risk patterns. The IC_50_ values of nine immunotherapeutic, chemical, and targeted agents, such as AUY922 and BMS.509744, were lower in the HRS group, demonstrating that the HRS group responded better to these treatments (**Figure 10**).

### 3.7 Consensus clustering and precision therapy

**Figure 11:**
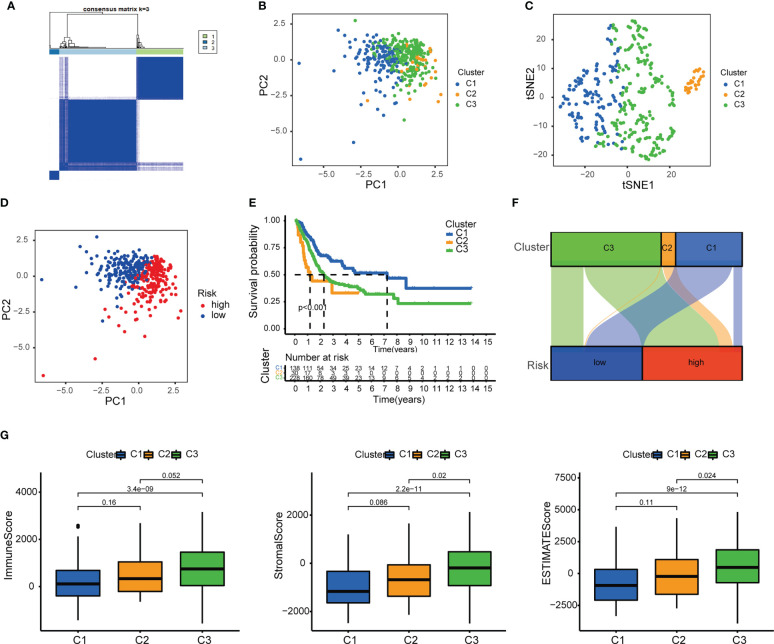
Differences between cold and hot tumors. **(A)** Patients were divided into three clusters by ConsensusClusterPlus. **(B)** PCA of clusters. **(C)** t-SNE of the three clusters. **(D)** PCA of risk groups. **(E)** Kaplan–Meier survival curves of each cluster. **(F)** The Sankey diagram of risk groups. **(G)** Comparison of immune scores among clusters 1–3.

**Figure 12:**
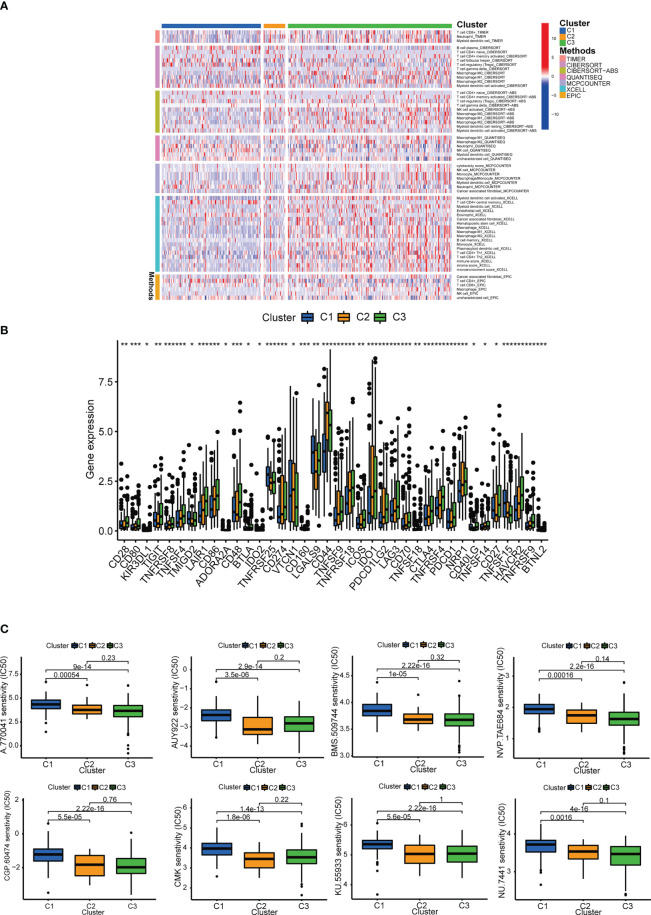
Prediction of immunotherapy efficacy. **(A)** Heatmap of immune cells in each cluster. **(B)** Differences in the expression of 18 immune checkpoints among the 3 clusters. **(C)** Immunotherapeutic agents that showed significantly different IC_50_ among the 3 clusters. *p<0.05, **p<0.01, and ***p<0.001.

The patients were grouped into 3 clusters according to inflammation-related lncRNAs expression (**Figure 11A**) (26). The three clusters were clearly distinguished by PCA (**Figure 11B**) and t-SNE analysis (**Figure 11C**), and the two risk groups were distinctly separated by PCA (**Figure 11D**). The OS rate was higher in cluster 1 than in clusters 2 and 3 (*P* < 0.001) (**Figure 11E**), and most BLCA patients in cluster 1 belonged to the LRS group, while most BLCA patients in clusters 2 and 3 belonged to the HRS group (**Figure 11F**). The immune and stromal cell scores of cluster 3 were the highest among the three clusters, and the immune score of cluster 2 was higher than that of cluster 1, which was consistent with the results of the Sankey diagram, and indicated a greater extent of immune cell infiltration in the HRS group. In addition, our data showed that TME varied among different clusters (**Figure 11G**), and immune cell infiltration was greater in clusters 2 and 3 (**Figure 12A**; **Supplementary Table 5**). The expression of most ICIs decreased as cluster number increased (cluster 1 < cluster 2 < cluster 3), including IDO1, CD274 (PD-L1), HAVCR2 (TIM3), and LAG3 (**Figure 12B**), which were highly effective therapeutic targets for hot tumors. We then classified clusters 2 and 3 as “hot” tumors and cluster 1 as a “cold” tumor based on their TME and responses to immunotherapy (27). It was found that clusters 2 and 3 (hot tumors) were highly associated with cell infiltration and more sensitive to immunotherapy. By comparing drug sensitivity, we discovered that 14 immunotherapeutic, chemical, or targeted agents, including AUY922, had lower IC_50_ values in clusters 2 and 3 (**Figure 12C**; **Supplementary Figure 2B**), which indicated that hot tumors responded better to these treatments.

## 4 Discussions

At present, BLCA is still a major global health concern, with a prevalence of about 500 to 50,000 new cases each year (28). BLCA is a highly differentiated cancer with a poor prognosis (29), and the 5-year OS rate for all BLCA stages is still less than 20% (30). Once the tumor reaches the locally advanced or metastatic stage, conventional chemotherapy treatments for BLCA are no longer effective (31). There is an urgent need for new diagnostic and therapeutic methods to assess the prognosis of bladder cancer and to provide guidance for individualized clinical treatment. Clinical biomarkers of urine in liquid biopsies have been shown to be useful in the diagnosis and prognosis of bladder cancer. Urine is easy to obtain, does not require special patient compliance, and has a simple composition, making it highly useful for clinical practice (32). In addition, ICIs have demonstrated to be effective treatments for BLCA and other malignant tumors. However, only a small number of patients have a lasting response to ICIs, and hence it is vital to identify more accurate response indicators to improve the efficacy of immunotherapy (33). Invasive tumors with high immune scores are known as “hot tumors”, whereas non-invasive tumors with low immune scores are known as “cold tumors”. For example, tumors with high expression of MHC class I and immune checkpoints are called “hot tumors”. However, ICIs are only effective for hot tumors and essentially ineffective for cold tumors (34). T cell targeted immunotherapy and other immunotherapeutic drugs can be used for the treatment of hot tumors. On the other hand, cold tumors, also known as immunosuppressive tumors, are difficult to be recognized by immune cells, resulting in fewer T cells infiltrating into and around the tumor (35, 36). CD8^+^ T cells are activated by tumor antigens and infiltrate the tumor. The application of anti-PD-1/PD-L1 antibodies can significantly enhance the efficacy of immunotherapy (34). In order to enhance anti-tumor immunity, immunotherapy alone is far from enough. Transforming cold tumors into hot tumors will be pivotal to improve the efficacy of immunotherapy (35).

In this study, we constructed a prognostic model using 7 inflammation-related lncRNAs. The new model classified patients into two groups, namely the HRS group and the LRS group, which were then subjected to Kaplan-Meier analysis, ROC analysis, GSEA analysis, and IC50 prediction. Our results showed that the risk group can serve as a guide for clinical evaluation and prognosis prediction, but cannot effectively distinguish hot tumors from cold tumors. We also observed that BCLA patients can be divided into different subtypes based on gene expression clustering. Each tumor subtype has a different TME, prognosis, and immune response (27). By dividing these lncRNAs into three groups through consensus clustering, we observed that cluster 1 had less CD8^+^ T cell infiltration and an immunosuppressive TME, while clusters 2 and 3 had greater CD8^+^ T infiltration, an increased immune score, and PD-L1, LAG3, and TIM3 expression. Therefore, we identified clusters 2 and 3 as “hot tumors” which are more susceptible to immunotherapy drugs (26, 35). It is known that immunotherapy such as anti-programmed death ligand (PD-L)1/PD-1 therapy is more effective for hot tumors. In order to achieve a better tumor response to immunotherapy, various studies have investigated the means of transforming cold tumors into hot tumors (37, 38). Our findings demonstrated that inflammation-related lncRNAs not only serve as prognostic markers that effectively differentiate between cold and hot tumors, but also provide a theoretical basis for individualized BCLA treatment.

Inflammation is an immune response to infection, trauma, or other stressors, and is crucial for maintaining overall health (39). Chronic inflammation can increase the risk of cancer and promote cancer growth by inducing tumorigenesis, progression, and metastasis (40). A growing number of studies have shown that tumorigenesis may be driven by an inflammatory TME, especially in gastric cancer (41), glioma (42), and colorectal cancer (43, 44). Therefore, inflammation may be a contributing factor to BCLA progression. We generated a 7 lncRNA-based risk marker to explore its correlation with BLCA. The functional roles of some of these selected lncRNAs in cancer progression have been reported in several studies. Jia et al. (45) found that LNCAROD is involved in tumor malignancy and chemoresistance, especially in a hypoxic microenvironment, by increasing PKM2 levels and ultimately aerobic glycolysis in cancer cells. Kuang et al. (46) reported that the lncRNA MAP3K14-AS1 has a prognostic marker for BLCA. Luo et al. (47) showed that NR2F1-AS1 can modulate cancer cell proliferation, invasion, migration, apoptosis, cell cycle, and glycolysis *via* various mechanisms, and is aberrantly expressed in numerous malignancies, which suggests that NR2F1-AS1 may be a potential therapeutic target and prognostic marker for cancer. He et al. (48) demonstrated that KCNQ1OT1 can recruit DNA methyltransferases to the EIF2B5 promoter to downregulate EIF2B5 expression and thereby promote ovarian cancer metastasis.

We calculated individual AUC values for ROC to obtain the most accurate model and the optimal cut-off point for distinguishing HRS and LRS BLCA patients. Kaplan-Meier analysis showed significantly shorter survival in the HRS group. Surprisingly, it was found that risk scores were related with BCLA staging. Furthermore, risk scores were significantly higher in high-grade BLCA patients than in low-grade BLCA patients, and were consistent with prognosis. As a result, we constructed a nomogram that integrates these characteristics to generate accurate predictions of clinical outcomes.

The GSEA of differentially expressed genes (DEGs) between the HRS and LRS groups showed that DEGs in the HRS group were significantly related with the chemokine signal pathway, leukocyte transendothelial migration, extracellular matrix (ECM) receptor signaling, and adherens junction, which are highly correlated with tumor invasion. Moreover, we also calculated the chemosensitivity of patients based on the IC_50_ value and screened for candidate small-molecule compounds.

Previous studies have shown that circulating tumor cells play an important role in tumor development and metastasis, and are closely related with survival of BLCA patients. Detection of circulating tumor cells can help assess the prognosis of BLCA patients and guide individualized clinical treatment (49).

Similarly, these inflammation-related lncRNAs can not only be used as prognostic predictors for BLCA prognosis but also provide guidance for clinical drug use, which was helpful for further experimental research and a better understanding of BLCA pathogenesis. However, this study also has several limitations. The risk assessment data of our lncRNAs originated from a public database, and there is a lack of more external transcriptome information to verify the role of these inflammation-related lncRNAs in BLCA. The specific molecular mechanism of inflammation-related lncRNAs in BLCA is not clear, and hence further studies are needed. On the other hand, heterogeneity among the normal and tumor samples may affect the accuracy of the data analysis. However, the construction of this model only requires tumor samples with clinical information, so it may not affect the accuracy of inflammation-related lncRNAs in predicting BLCA risk. The immune cell results were calculated using different platforms, which can be regarded as an external verification. Collectively, our findings may open new avenues for finding suitable treatment alternatives for BLCA patients, and our model is feasible for future exploration of BCLA pathogenesis and clinical guidance in BCLA treatment. Since both lncRNAs and inflammatory signaling pathways are involved in the progression of tumor cells (50), further understanding of their molecular mechanisms will improve treatment efficacy and reduce treatment-related toxicities in BCLA patients.

## 5 Conclusions

In summary, this study provides a valid model for prognosis prediction and clinical management of BLCA patients, thus providing a reference for the development of individualized tumor therapy. Risk scores can predict the sensitivity of patients to chemotherapy. Inflammation-related lncRNAs can predict prognosis by identifying hot and cold tumors, and provide therapeutic strategies, which will greatly improve the individualized therapy and patient prognosis. Understanding the mechanisms of inflammation-related lncRNAs in BLCA may provide new insights to treatment strategies for BCLA and other cancers.

## Data availability statement

The original contributions presented in the study are included in the article/**Supplementary Material**. Further inquiries can be directed to the corresponding authors.

## Ethics statement

Written informed consent was obtained from the individual(s) for the publication of any potentially identifiable images or data included in this article.

## Author contributions

PL designed the project and supervised the study. XX and LL contributed to the data analysis and original draft preparation. XX and CC conducted the experiments and revised the article. LL, XL, and MP reviewed and edited the manuscript. JY and WZ provided valuable suggestions for study. XW and HZ edited the language. All authors contributed to the article and approved the submitted version.
