# Supplementary material for: Identification of a novel defined inflammation-related long noncoding RNA signature contributes to predicting prognosis and distinction between the cold and hot tumors in bladder cancer
Source: Front Oncol. 2023 Mar 29;13:972558. doi: 10.3389/fonc.2023.972558 (PMC10090514; doi:10.3389/fonc.2023.972558)
Supplement: Supplementary file 1 [file DataSheet_1.pdf]

Supplementary Material

Supplementary Figures

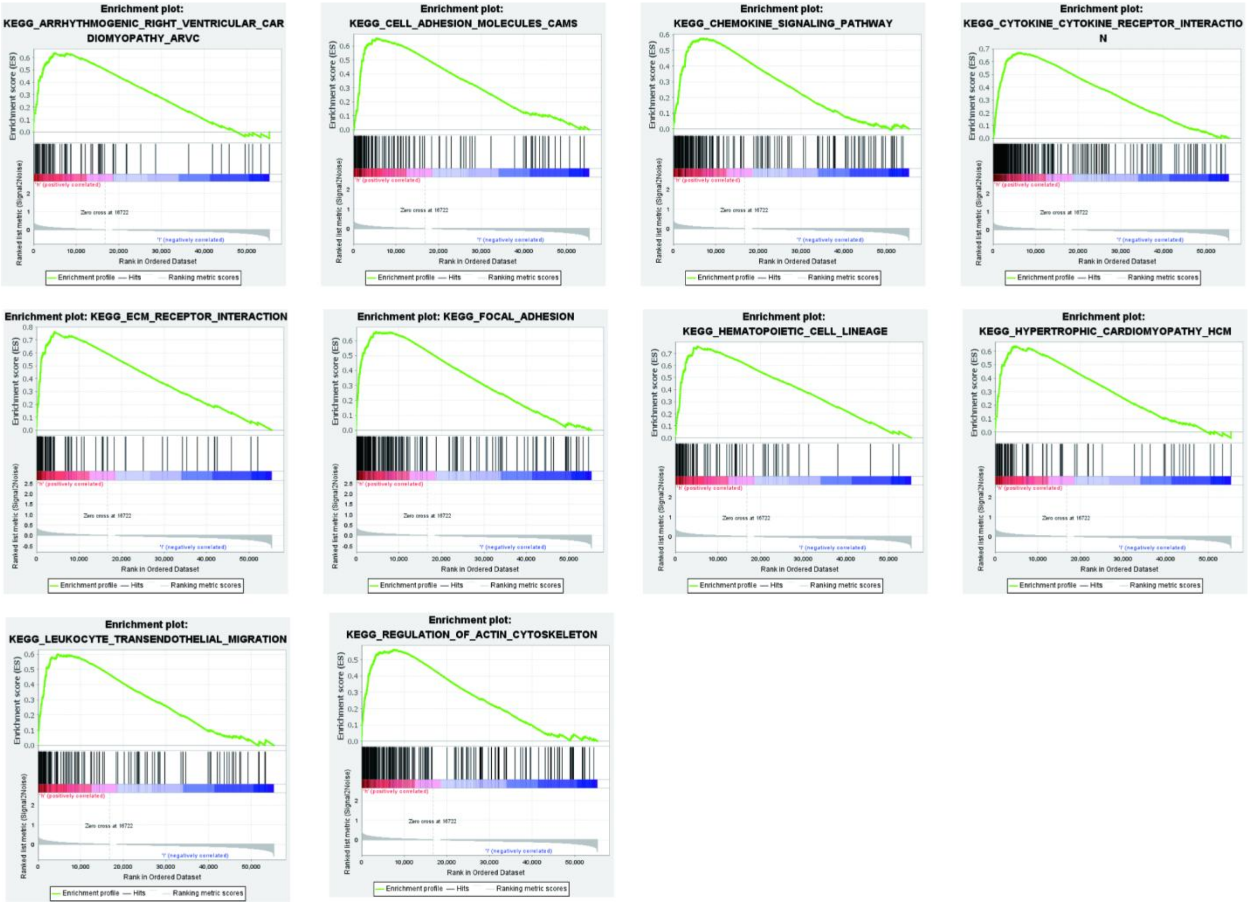

Supplementary Figure 1. The GSEA result of the high-risk group in cluster 2 and cluster 3.

A

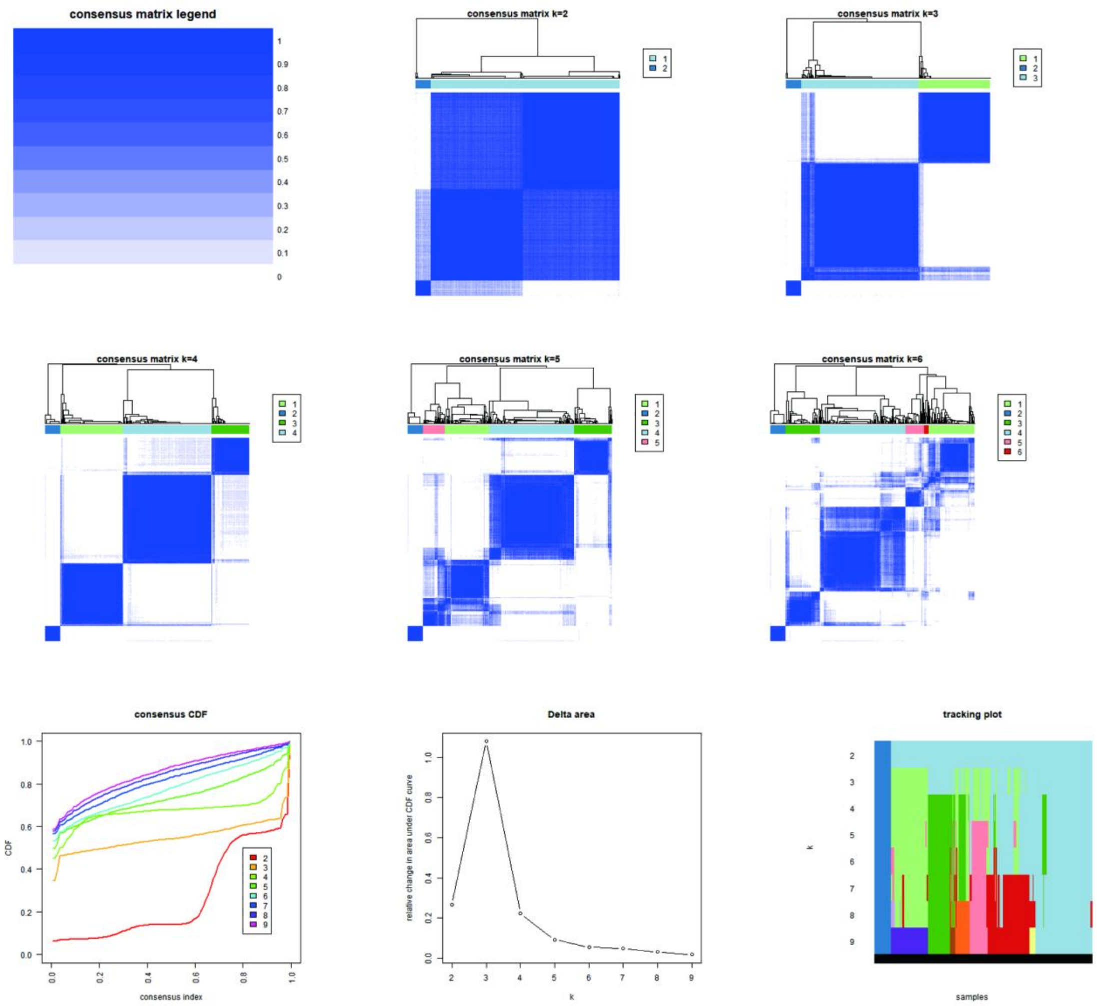
consensus matrix legend
consensus matrix k=2
consensus matrix k=3
consensus matrix k=4
consensus matrix k=5
consensus matrix k=6
consensus CDF
CDF
consensus index
Delta area
relative change in area under CDF curve
k
tracking plot
samples

B

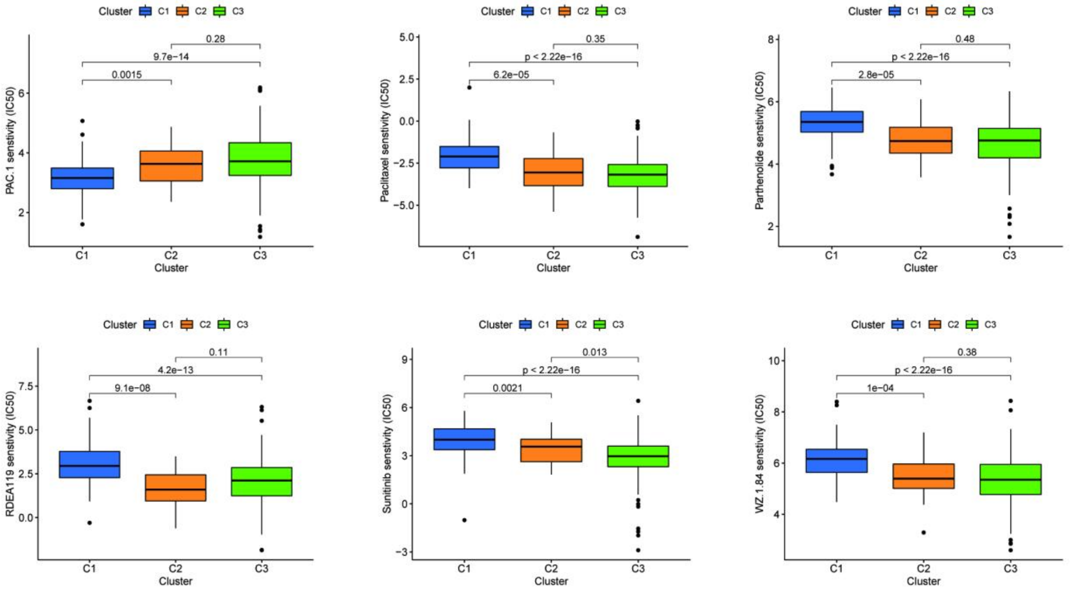
Cluster C1 C2 C3
PAC.1 sensitivity (IC50)
0.28
9.7e−14
0.0015
Paclitaxel sensitivity (IC50)
0.35
p < 2.22e−16
6.2e−05
Parthenolide sensitivity (IC50)
0.48
p < 2.22e−16
2.8e−05
RDEA119 sensitivity (IC50)
0.11
4.2e−13
9.1e−08
Sunitinib sensitivity (IC50)
0.013
p < 2.22e−16
0.0021
WZ.1.84 sensitivity (IC50)
0.38
p < 2.22e−16
1e−04
Cluster

Supplementary Figure 2. Consensus clustering analysis of inflammation-related lncRNAs and IC50 prediction in clusters. (A) The heatmap, cumulative distribution function (CDF) plot, and the consensus CDF plots. (B) 6 chemical or targeted drugs with different IC50.
