# Supplementary material for: Identification of a novel defined inflammation-related long noncoding RNA signature contributes to predicting prognosis and distinction between the cold and hot tumors in bladder cancer
Source: Front Oncol. 2023 Mar 29;13:972558. doi: 10.3389/fonc.2023.972558 (PMC10090514; doi:10.3389/fonc.2023.972558)
Supplement: Supplementary file 2 [file DataSheet_2.pdf]

Supplementary Material

Supplementary Tables

Supplementary Table 1. qRT-PCR primers.

Gene symbol	Forward primer (5’-3’)	Reverse primer (5’-3’)
AC068196.1	ACTGTCTGGCTATCCTGTCG	ACTATACCTTGAAGAAGATACCACA
LNCAROD	GTGCATGCTCCATCACATCA	TAAACTCTCCAGGGCTGCTTG
MAP3K14-AS1	GTTCTTCCTGCACAGTCGGT	TGGGAGCTCTTGAGTTGGTG
AC021321.1	AGTTCCAATGTTGCCATCTGC	CACATTGCTTTCCTGCCACTG
LINC02256	AGTCCCAGGCATAACTGAAGG	TGTCTTCTTTCTTCACTAGCCGA
NR2F1-AS1	CGCGAGGGCGTAAAAGTTTG	AGCGTGGCCATTTACTTCCA
KCNQ1OT1	GCTTGAGGACCAGCAACAAAG	GTCGCTTGCCCAACCAGTAT

Supplementary Table 2. 200 inflammation-related genes were obtained from the Molecular Signatures Database.

Gene
ABCA1
ABI1
ACVR1B
ACVR2A
ADM
ADORA2B
ADRM1
AHR
APLNR
AQP9

ATP2A2

ATP2B1

ATP2C1

AXL

BDKRB1

BEST1

BST2

BTG2

C3AR1

C5AR1

CALCRL

CCL17

CCL2

CCL20

CCL22

CCL24

CCL5

CCL7

CCR7

CCRL2

CD14

CD40

CD48

CD55

CD69

CD70

CD82

CDKN1A

CHST2

CLEC5A

CMKLR1

CSF1

CSF3

CSF3R

CX3CL1

CXCL10

CXCL11

CXCL6

CXCL9

CXCR6

CYBB

DCBLD2

EBI3

EDN1

EIF2AK2

EMP3

ADGRE1

EREG

F3

FFAR2

FPR1

FZD5

GABBR1

GCH1

GNA15

GNAI3

GP1BA

GPC3

GPR132

GPR183

HAS2

HBEGF

HIF1A

HPN

HRH1

ICAM1

ICAM4

ICOSLG

IFITM1

IFNAR1

IFNGR2

IL10

IL10RA

IL12B

IL15

IL15RA

IL18

IL18R1

IL18RAP

IL1A

IL1B

IL1R1

IL2RB

IL4R

IL6

IL7R

CXCL8

INHBA

IRAK2

IRF1

IRF7

ITGA5

ITGB3

ITGB8

KCNA3

KCNJ2

KCNMB2

KIF1B

KLF6

LAMP3

LCK

LCP2

LDLR

LIF

LPAR1

LTA

LY6E

LYN

MARCO

MEFV

MEP1A

MET

MMP14

MSR1

MXD1

MYC

NAMPT

NDP

NFKB1

NFKBIA

NLRP3

NMI

NMUR1

NOD2

NPFFR2

OLR1

OPRK1

OSM

OSMR

P2RX4

P2RX7

P2RY2

PCDH7

PDE4B

PDPN

PIK3R5

PLAUR

PROK2

PSEN1

PTAFR

PTGER2

PTGER4

PTGIR

PTPRE

PVR

RAF1

RASGRP1

RELA

RGS1

RGS16

RHOG

RIPK2

RNF144B

ROS1

RTP4

SCARF1

SCN1B

SELE

SELL

SELENOS

SEMA4D

SERPINE1

SGMS2

SLAMF1

SLC11A2

SLC1A2

SLC28A2

SLC31A1

SLC31A2

SLC4A4

SLC7A1

SLC7A2

SPHK1

SRI

STAB1

TACR1

TACR3

TAPBP

TIMP1

TLR1

TLR2
TLR3
TNFAIP6
TNFRSF1B
TNFRSF9
TNFSF10
TNFSF15
TNFSF9
TPBG
VIP

Supplementary Table 3. The network data of inflammation-related genes and lncRNAs.

Gene	LncRNA	Cor	*P*-value	Regulation
CCL20	LINC02015	0.471583873	2.59E-24	postive
EREG	LINC02015	0.528129837	4.10E-31	postive
ACVR2A	PDXDC2P-NPIPB14P	0.425114453	1.34E-19	postive
SPHK1	AL441992.1	0.434302401	1.79E-20	postive
CXCL10	KLHDC7B-DT	0.502886077	6.40E-28	postive
CXCL11	KLHDC7B-DT	0.492617305	1.07E-26	postive
IFITM1	KLHDC7B-DT	0.515387432	1.81E-29	postive

IL15	KLHDC7B-DT	0.463566702	1.90E-23	postive
IL15RA	KLHDC7B-DT	0.512866819	3.76E-29	postive
IL18RAP	KLHDC7B-DT	0.414534421	1.27E-18	postive
IRF1	KLHDC7B-DT	0.498884044	1.94E-27	postive
LAMP3	KLHDC7B-DT	0.420677654	3.48E-19	postive
NMI	KLHDC7B-DT	0.569637508	5.51E-37	postive
NOD2	KLHDC7B-DT	0.402108899	1.61E-17	postive
ABCA1	Z98885.3	0.719782505	2.68E-67	postive
FZD5	Z98885.3	0.55963609	1.71E-35	postive
KCNMB2	Z98885.3	0.440398005	4.54E-21	postive
KIF1B	Z98885.3	0.404523429	9.89E-18	postive
TACR1	Z98885.3	0.433141417	2.31E-20	postive
KCNMB2	HOXC-AS1	0.43092038	3.78E-20	postive
KCNMB2	CAMTA1-DT	0.482564201	1.55E-25	postive
ABCA1	AC026124.2	0.583407243	4.01E-39	postive
FZD5	AC026124.2	0.530544569	1.97E-31	postive
KCNMB2	AC026124.2	0.45068436	4.20E-22	postive
FZD5	AC010300.1	0.631622501	1.77E-47	postive
KCNMB2	AC010300.1	0.461897642	2.86E-23	postive
PTGIR	AP001434.1	0.422089456	2.57E-19	postive

TIMP1	AP001434.1	0.457369651	8.58E-23	postive
FZD5	MORF4L2-AS1	0.434348335	1.77E-20	postive
KCNMB2	AL353708.3	0.422909432	2.16E-19	postive
CSF3R	AC004471.1	0.418820333	5.16E-19	postive
ABCA1	DHDDS-AS1	0.682038934	5.89E-58	postive
KCNMB2	DHDDS-AS1	0.436875369	1.01E-20	postive
KIF1B	DHDDS-AS1	0.427892668	7.34E-20	postive
ACVR2A	GRHL3-AS1	0.450307222	4.59E-22	postive
AXL	HHIP-AS1	0.47549764	9.61E-25	postive
NFKB1	SNHG10	-0.400686094	2.13E-17	negative
CD48	LINC02361	0.434015886	1.91E-20	postive
CXCR6	LINC02361	0.468131761	6.16E-24	postive
GPR132	LINC02361	0.454341164	1.77E-22	postive
IL10RA	LINC02361	0.455054088	1.49E-22	postive
IL2RB	LINC02361	0.43005575	4.57E-20	postive
LCK	LINC02361	0.520018248	4.66E-30	postive
LCP2	LINC02361	0.430833285	3.86E-20	postive
LTA	LINC02361	0.502039514	8.11E-28	postive
TNFRSF1B	LINC02361	0.451711057	3.30E-22	postive
BDKRB1	GAPLINC	0.451249108	3.68E-22	postive

BEST1	GAPLINC	0.414118336	1.38E-18	postive
C3AR1	GAPLINC	0.532874585	9.61E-32	postive
C5AR1	GAPLINC	0.558968162	2.14E-35	postive
CD14	GAPLINC	0.526056854	7.69E-31	postive
CLEC5A	GAPLINC	0.42718753	8.56E-20	postive
CMKLR1	GAPLINC	0.513550544	3.09E-29	postive
CSF1	GAPLINC	0.558477693	2.52E-35	postive
EMP3	GAPLINC	0.601689332	4.00E-42	postive
FPR1	GAPLINC	0.550186314	3.94E-34	postive
NLRP3	GAPLINC	0.466934438	8.29E-24	postive
OSM	GAPLINC	0.449493318	5.56E-22	postive
PLAUR	GAPLINC	0.467719559	6.82E-24	postive
PTGER2	GAPLINC	0.403314404	1.26E-17	postive
RHOG	GAPLINC	0.415667382	1.00E-18	postive
SCN1B	GAPLINC	0.439072855	6.13E-21	postive
SPHK1	GAPLINC	0.426259739	1.05E-19	postive
TIMP1	GAPLINC	0.613711284	3.32E-44	postive
TNFAIP6	GAPLINC	0.444763161	1.67E-21	postive
CD48	AC090152.1	0.417221091	7.23E-19	postive
EBI3	AC090152.1	0.460415526	4.11E-23	postive

SLAMF1	AC090152.1	0.517418415	1.00E-29	postive
ABCA1	AC011933.3	0.603773779	1.77E-42	postive
KCNMB2	AC011933.3	0.402211103	1.57E-17	postive
ABCA1	AC092755.2	0.441665713	3.40E-21	postive
ABCA1	AC004771.1	0.507575926	1.71E-28	postive
KCNMB2	AC004771.1	0.416232258	8.90E-19	postive
FZD5	WARS2-AS1	0.433685089	2.05E-20	postive
ABCA1	AC018766.1	0.454661745	1.64E-22	postive
FZD5	AC005537.1	0.410444551	2.96E-18	postive
ABCA1	AC007390.1	0.576455626	4.95E-38	postive
FZD5	AC007390.1	0.414496905	1.28E-18	postive
ITGA5	AC068506.1	0.600798707	5.66E-42	postive
ABCA1	AC022001.3	0.650483047	3.65E-51	postive
ABCA1	AL161719.1	0.454067365	1.89E-22	postive
CYBB	AL161719.1	0.47155645	2.61E-24	postive
TLR1	AL161719.1	0.507059896	1.98E-28	postive
BEST1	LINC01852	0.493355835	8.80E-27	postive
CMKLR1	LINC01852	0.452709366	2.61E-22	postive
CSF1	LINC01852	0.434493103	1.71E-20	postive
EMP3	LINC01852	0.466529695	9.17E-24	postive

IL10RA	LINC01852	0.418420275	5.61E-19	postive
NMUR1	LINC01852	0.463373836	2.00E-23	postive
PTGER2	LINC01852	0.413950716	1.43E-18	postive
SCN1B	LINC01852	0.478636074	4.30E-25	postive
TIMP1	LINC01852	0.48580863	6.61E-26	postive
EMP3	FAM225A	0.591941663	1.68E-40	postive
SCN1B	FAM225A	0.484389815	9.61E-26	postive
TIMP1	FAM225A	0.412955722	1.76E-18	postive
ABCA1	WASHC5-AS1	0.637507536	1.34E-48	postive
FZD5	WASHC5-AS1	0.622995211	7.09E-46	postive
KCNMB2	WASHC5-AS1	0.449806884	5.17E-22	postive
SLC11A2	WASHC5-AS1	0.449457944	5.61E-22	postive
CXCR6	AC018926.2	0.460781004	3.76E-23	postive
RGS1	AC018926.2	0.41762471	6.64E-19	postive
ABCA1	AL133243.3	0.69107915	4.61E-60	postive
ABCA1	AC008543.3	0.490794811	1.75E-26	postive
FZD5	AC008543.3	0.580925654	9.90E-39	postive
KCNMB2	AC008543.3	0.450864333	4.03E-22	postive
SLC11A2	AC008543.3	0.452163361	2.97E-22	postive
ABCA1	GARS1-DT	0.537003221	2.67E-32	postive

FZD5	GARS1-DT	0.524660675	1.17E-30	postive
ABCA1	AC019186.1	0.414529498	1.27E-18	postive
FZD5	AC019186.1	0.428661758	6.21E-20	postive
NOD2	MYOSLID	0.44671053	1.06E-21	postive
ABCA1	AC025031.3	0.514487856	2.35E-29	postive
FZD5	AC025031.3	0.429158296	5.57E-20	postive
ABCA1	AC022973.4	0.747956784	2.42E-75	postive
FZD5	AC022973.4	0.546256387	1.41E-33	postive
KCNMB2	AC022973.4	0.439675225	5.34E-21	postive
TACR1	AC022973.4	0.462354251	2.56E-23	postive
C5AR1	LINC01638	0.447423419	9.01E-22	postive
FPR1	LINC01638	0.45675022	9.95E-23	postive
PTGIR	LINC01638	0.413731765	1.50E-18	postive
SCN1B	LINC01638	0.451296951	3.64E-22	postive
TIMP1	LINC01638	0.48158213	2.00E-25	postive
ABCA1	GSN-AS1	0.796347533	5.35E-92	postive
CYBB	GSN-AS1	0.482920306	1.41E-25	postive
FZD5	GSN-AS1	0.42708351	8.76E-20	postive
KIF1B	GSN-AS1	0.431980104	2.99E-20	postive
TACR1	GSN-AS1	0.464465824	1.53E-23	postive

TLR1	GSN-AS1	0.503200778	5.86E-28	postive
ABCA1	AL365436.2	0.500472793	1.25E-27	postive
CCL24	LINC01614	0.540128726	9.98E-33	postive
MMP14	LINC01614	0.525448751	9.23E-31	postive
NMUR1	LINC01614	0.44799343	7.89E-22	postive
ABCA1	AC129510.1	0.481106102	2.27E-25	postive
FZD5	Z93403.1	0.516671044	1.25E-29	postive
ABCA1	MCM3AP-AS1	0.527318218	5.25E-31	postive
KIF1B	MCM3AP-AS1	0.424153603	1.65E-19	postive
ITGA5	LINC02489	0.57206457	2.35E-37	postive
CALCRL	AP001189.1	0.49888327	1.94E-27	postive
ABCA1	AC105339.2	0.449674973	5.33E-22	postive
STAB1	AC019077.1	0.546982504	1.12E-33	postive
FZD5	AC098484.1	0.523510195	1.65E-30	postive
ABCA1	AC015853.1	0.519679102	5.15E-30	postive
ABCA1	AC026771.1	0.540675213	8.39E-33	postive
KCNMB2	AC026771.1	0.41055278	2.90E-18	postive
ABCA1	AC092802.2	0.566187423	1.82E-36	postive
FZD5	AC092802.2	0.644499585	5.75E-50	postive
KCNMB2	AC092802.2	0.458317182	6.83E-23	postive

SLC11A2	AC092802.2	0.457195722	8.94E-23	postive
NOD2	AC015660.1	0.444281484	1.87E-21	postive
FZD5	LINC01290	0.451307287	3.63E-22	postive
ABCA1	AC025031.1	0.575921797	5.99E-38	postive
CALCRL	AC025031.1	0.438815825	6.49E-21	postive
FZD5	AC025031.1	0.698669822	6.82E-62	postive
GPC3	AC025031.1	0.446109083	1.22E-21	postive
KCNMB2	AC025031.1	0.496698894	3.54E-27	postive
SLC11A2	AC025031.1	0.433343313	2.21E-20	postive
ABCA1	AL133415.1	0.613937991	3.02E-44	postive
BEST1	AL133415.1	0.412479109	1.95E-18	postive
CMKLR1	AL133415.1	0.491723407	1.37E-26	postive
CSF1	AL133415.1	0.427223132	8.50E-20	postive
CYBB	AL133415.1	0.699473552	4.33E-62	postive
IL10RA	AL133415.1	0.578225832	2.63E-38	postive
MEFV	AL133415.1	0.41278389	1.83E-18	postive
NLRP3	AL133415.1	0.579097006	1.92E-38	postive
PIK3R5	AL133415.1	0.692582319	2.02E-60	postive
SCARF1	AL133415.1	0.437308535	9.12E-21	postive
SCN1B	AL133415.1	0.423859789	1.76E-19	postive

STAB1	AL133415.1	0.865932253	4.89E-126	postive
TACR1	AL133415.1	0.670764192	1.97E-55	postive
TLR1	AL133415.1	0.598981893	1.14E-41	postive
TNFRSF1B	AL133415.1	0.421888058	2.69E-19	postive
BDKRB1	AC093627.6	0.438896514	6.38E-21	postive
KCNMB2	AC010247.2	0.628447161	6.98E-47	postive
KCNMB2	LINC02525	0.439794147	5.20E-21	postive
CXCR6	AL137186.1	0.411263908	2.50E-18	postive
PTAFR	AL137186.1	0.418903079	5.07E-19	postive
ITGA5	ACTA2-AS1	0.657356277	1.42E-52	postive
ABCA1	AC124283.3	0.652329192	1.54E-51	postive
ABCA1	AC019183.1	0.690764094	5.48E-60	postive
CALCRL	AC019183.1	0.460073824	4.46E-23	postive
CYBB	AC019183.1	0.436275153	1.15E-20	postive
FZD5	AC019183.1	0.57329972	1.52E-37	postive
GPC3	AC019183.1	0.404278009	1.04E-17	postive
TLR1	AC019183.1	0.465618314	1.15E-23	postive
ABCA1	RAP2C-AS1	0.54691904	1.14E-33	postive
KIF1B	RAP2C-AS1	0.502482456	7.17E-28	postive
ABCA1	AC073896.3	0.40257498	1.46E-17	postive

ABCA1	AF230666.1	0.543015747	3.99E-33	postive
FZD5	AF230666.1	0.556656956	4.64E-35	postive
ABCA1	UBOX5-AS1	0.508100487	1.47E-28	postive
F3	AC245041.1	0.425615249	1.20E-19	postive
ABCA1	AC087286.4	0.714609235	6.28E-66	postive
KIF1B	AC087286.4	0.404185557	1.06E-17	postive
TLR1	AC087286.4	0.407321106	5.61E-18	postive
CD70	AC113346.1	0.566328179	1.74E-36	postive
NOD2	AC113346.1	0.447897614	8.07E-22	postive
TNFSF9	AC113346.1	0.487911008	3.79E-26	postive
ABCA1	AC092756.1	0.58507754	2.17E-39	postive
LAMP3	AC116407.2	0.426526204	9.89E-20	postive
RTP4	AC116407.2	0.404848393	9.26E-18	postive
NOD2	AL031058.1	0.405457182	8.19E-18	postive
FZD5	AC009955.2	0.456485205	1.06E-22	postive
GPC3	AC009955.2	0.408795657	4.15E-18	postive
IL10RA	RRN3P2	0.400726361	2.12E-17	postive
MXD1	CERS3-AS1	0.445499331	1.41E-21	postive
ABCA1	DENND6A-AS1	0.640269702	3.89E-49	postive
ABCA1	PRR7-AS1	0.613459506	3.67E-44	postive

KIF1B	PRR7-AS1	0.401957887	1.66E-17	postive
FZD5	Z98200.1	0.402491357	1.49E-17	postive
FZD5	MIR29B2CHG	0.446977754	1.00E-21	postive
ABCA1	PABPC4-AS1	0.604443165	1.36E-42	postive
FZD5	PABPC4-AS1	0.557899472	3.06E-35	postive
SLC11A2	PABPC4-AS1	0.432914911	2.43E-20	postive
MET	AL590004.3	0.530832641	1.80E-31	postive
ACVR2A	AC005253.1	0.419877568	4.12E-19	postive
ABCA1	AL158163.2	0.446204897	1.20E-21	postive
ABCA1	AL929236.1	0.436115046	1.19E-20	postive
FZD5	AL929236.1	0.509179433	1.08E-28	postive
ABCA1	AL358216.1	0.588286163	6.62E-40	postive
FZD5	AL358216.1	0.448892495	6.40E-22	postive
KCNMB2	AL358216.1	0.400145955	2.38E-17	postive
ABCA1	LINC-PINT	0.727284229	2.43E-69	postive
BEST1	LINC-PINT	0.406678164	6.39E-18	postive
CYBB	LINC-PINT	0.495053992	5.55E-27	postive
TACR1	LINC-PINT	0.435192915	1.47E-20	postive
TLR1	LINC-PINT	0.513580565	3.06E-29	postive
C3AR1	LINC01050	0.45648303	1.06E-22	postive

CCL24	LINC01050	0.832888818	6.58E-108	postive
CMKLR1	LINC01050	0.403118859	1.31E-17	postive
ACVR2A	ZNF436-AS1	0.424061122	1.68E-19	postive
FZD5	ZNF436-AS1	0.469468791	4.41E-24	postive
PLAUR	ZNF436-AS1	-0.420036641	3.98E-19	negative
RHOG	ZNF436-AS1	-0.409140744	3.87E-18	negative
SLC11A2	ZNF436-AS1	0.419653242	4.32E-19	postive
ABCA1	AC091906.1	0.544606236	2.40E-33	postive
FZD5	AC091906.1	0.61489546	2.04E-44	postive
GPC3	AC091906.1	0.40357534	1.20E-17	postive
KCNMB2	AC091906.1	0.467387252	7.41E-24	postive
SLC11A2	AC091906.1	0.456225918	1.13E-22	postive
ABCA1	MAP3K5-AS1	0.583554778	3.80E-39	postive
FZD5	MAP3K5-AS1	0.536515619	3.10E-32	postive
KCNMB2	MAP3K5-AS1	0.408325659	4.57E-18	postive
MMP14	LINC01561	0.579948763	1.41E-38	postive
ABCA1	GTF3C2-AS1	0.555390839	7.08E-35	postive
FZD5	GTF3C2-AS1	0.585047668	2.19E-39	postive
KCNMB2	GTF3C2-AS1	0.436638919	1.06E-20	postive
SLC11A2	GTF3C2-AS1	0.461004349	3.56E-23	postive

FZD5	AC004233.1	0.507414436	1.79E-28	postive
GPC3	AC004233.1	0.400041821	2.43E-17	postive
KCNMB2	AC004233.1	0.417843042	6.34E-19	postive
SLC11A2	AC004233.1	0.407128869	5.83E-18	postive
PTGIR	AL445423.1	0.410358375	3.01E-18	postive
SCN1B	AL445423.1	0.400255536	2.32E-17	postive
HBEGF	MIR23AHG	0.405362762	8.35E-18	postive
GP1BA	AC005332.7	0.635802591	2.84E-48	postive
LTA	AC005332.7	0.43609155	1.20E-20	postive
ABCA1	AC007622.2	0.645683346	3.35E-50	postive
FZD5	AC007622.2	0.582373806	5.85E-39	postive
KCNMB2	AC007622.2	0.441060425	3.90E-21	postive
SLC11A2	AC007622.2	0.460624464	3.90E-23	postive
ABCA1	AC127024.3	0.639634321	5.17E-49	postive
FZD5	AC021491.4	0.692056323	2.70E-60	postive
GPC3	AC021491.4	0.507506297	1.74E-28	postive
KCNMB2	AC021491.4	0.421332656	3.02E-19	postive
SLC11A2	AC021491.4	0.442212102	3.00E-21	postive
ABCA1	AL080317.2	0.493376682	8.75E-27	postive
FZD5	AL080317.2	0.671840817	1.14E-55	postive

KCNMB2	AL080317.2	0.484273129	9.91E-26	postive
SLC11A2	AL080317.2	0.479483053	3.45E-25	postive
ABCA1	AC022211.1	0.490020123	2.16E-26	postive
IL4R	AC106739.1	0.54752234	9.37E-34	postive
ABCA1	AL442125.2	0.474807276	1.15E-24	postive
KIF1B	AL442125.2	0.421908844	2.67E-19	postive
MET	AL442125.2	0.475294785	1.01E-24	postive
FZD5	ZNF252P-AS1	0.508475558	1.32E-28	postive
ABCA1	LAMTOR5-AS1	0.615052319	1.92E-44	postive
CALCRL	LAMTOR5-AS1	0.421301601	3.04E-19	postive
FZD5	LAMTOR5-AS1	0.670216166	2.59E-55	postive
GPC3	LAMTOR5-AS1	0.455401513	1.37E-22	postive
KCNMB2	LAMTOR5-AS1	0.53348459	7.96E-32	postive
SLC11A2	LAMTOR5-AS1	0.472791919	1.91E-24	postive
ABCA1	AL137779.1	0.717299036	1.23E-66	postive
CYBB	AL137779.1	0.469310741	4.59E-24	postive
TLR1	AL137779.1	0.48452975	9.26E-26	postive
FZD5	AC011503.2	0.500230919	1.34E-27	postive
SLC11A2	AC011503.2	0.411943778	2.17E-18	postive
ABCA1	AC022272.1	0.621673774	1.24E-45	postive

FZD5	AC022272.1	0.643994373	7.24E-50	postive
KCNMB2	AC022272.1	0.491571866	1.42E-26	postive
SLC11A2	AC022272.1	0.43102435	3.70E-20	postive
ABCA1	AP000766.1	0.449459279	5.60E-22	postive
CALCRL	AP000766.1	0.401668095	1.75E-17	postive
FZD5	AP000766.1	0.567371529	1.21E-36	postive
KCNMB2	AP000766.1	0.420141147	3.90E-19	postive
SLC11A2	AP000766.1	0.444196453	1.90E-21	postive
FZD5	AL645608.1	0.493958693	7.47E-27	postive
ABCA1	Z82243.1	0.734136361	2.87E-71	postive
CYBB	Z82243.1	0.468648677	5.41E-24	postive
KIF1B	Z82243.1	0.443270708	2.35E-21	postive
STAB1	Z82243.1	0.429742011	4.90E-20	postive
TACR1	Z82243.1	0.47875155	4.17E-25	postive
TLR1	Z82243.1	0.465418638	1.21E-23	postive
FZD5	AC106820.5	0.484881088	8.45E-26	postive
KCNMB2	AC106820.5	0.500350573	1.30E-27	postive
KIF1B	AC106820.5	0.42568003	1.19E-19	postive
SLC11A2	AC106820.5	0.407889578	5.00E-18	postive
ABCA1	AC008635.1	0.598856044	1.20E-41	postive

ABCA1	AC020978.1	0.617439109	7.19E-45	postive
FZD5	AC020978.1	0.414995875	1.15E-18	postive
ABCA1	AC005519.1	0.411625636	2.32E-18	postive
ABCA1	AC110609.1	0.529425481	2.77E-31	postive
CYBB	AC110609.1	0.431333034	3.45E-20	postive
TLR1	AC110609.1	0.44862965	6.81E-22	postive
SLC4A4	PPP1R26-AS1	0.44602629	1.25E-21	postive
ABCA1	Z93930.3	0.447303261	9.27E-22	postive
FZD5	Z93930.3	0.441759924	3.33E-21	postive
KCNMB2	RNF139-AS1	0.402189369	1.58E-17	postive
FZD5	AC011978.1	0.509335603	1.04E-28	postive
ABCA1	AC004846.2	0.495631803	4.74E-27	postive
CYBB	AC004846.2	0.456129689	1.15E-22	postive
STAB1	AC004846.2	0.415290291	1.08E-18	postive
TLR1	AC004846.2	0.451836975	3.20E-22	postive
CD40	AL133346.1	0.558860489	2.22E-35	postive
CD48	AL133346.1	0.632397297	1.26E-47	postive
EBI3	AL133346.1	0.780783816	3.52E-86	postive
LCK	AL133346.1	0.405864681	7.54E-18	postive
PTGIR	AL133346.1	0.445884692	1.29E-21	postive

SLAMF1	AL133346.1	0.941613771	6.95E-197	postive
ABCA1	AC089999.2	0.547925619	8.22E-34	postive
FZD5	AC089999.2	0.450429138	4.46E-22	postive
CD48	LINC00892	0.568659677	7.75E-37	postive
GP1BA	LINC00892	0.653652512	8.25E-52	postive
IL10RA	LINC00892	0.485812504	6.61E-26	postive
KCNA3	LINC00892	0.43855353	6.89E-21	postive
LCK	LINC00892	0.651060435	2.79E-51	postive
LCP2	LINC00892	0.420901567	3.31E-19	postive
LTA	LINC00892	0.733687082	3.86E-71	postive
TNFRSF9	LINC00892	0.442279462	2.95E-21	postive
ABCA1	AL590369.1	0.451968548	3.11E-22	postive
BDKRB1	AL583785.1	0.508636422	1.27E-28	postive
C5AR1	AL583785.1	0.495834291	4.49E-27	postive
FPR1	AL583785.1	0.513341522	3.28E-29	postive
IL6	AL583785.1	0.419859677	4.14E-19	postive
PROK2	AL583785.1	0.424303218	1.60E-19	postive
TNFAIP6	AL583785.1	0.425852584	1.14E-19	postive
ABCA1	AC092611.1	0.4703952	3.50E-24	postive
FZD5	AC092611.1	0.590706776	2.68E-40	postive

GPC3	AC092611.1	0.421019637	3.23E-19	postive
SLC11A2	AC092611.1	0.472920905	1.85E-24	postive
CSF1	AC010503.4	-0.403551647	1.20E-17	negative
ITGA5	AC010503.4	-0.405427068	8.24E-18	negative
MMP14	AC010503.4	-0.426185654	1.06E-19	negative
OSMR	AC010503.4	-0.503756898	5.02E-28	negative
ABCA1	AL109923.1	0.599795454	8.35E-42	postive
FZD5	AL109923.1	0.570160827	4.59E-37	postive
KCNMB2	AL109923.1	0.455830943	1.24E-22	postive
ABCA1	AC121764.1	0.555587034	6.63E-35	postive
CALCRL	AC121764.1	0.447332065	9.21E-22	postive
FZD5	AC121764.1	0.598629504	1.31E-41	postive
GPC3	AC121764.1	0.480942524	2.37E-25	postive
SLC11A2	AC121764.1	0.401451187	1.83E-17	postive
ABCA1	TMEM202-AS1	0.414377689	1.31E-18	postive
ABCA1	AL445649.1	0.47153235	2.63E-24	postive
CYBB	AL445649.1	0.540047176	1.02E-32	postive
TLR1	AL445649.1	0.547698246	8.85E-34	postive
ABCA1	LINC02649	0.636314026	2.27E-48	postive
TLR1	LINC02649	0.40468269	9.58E-18	postive

FZD5	AC007255.1	0.502802636	6.55E-28	postive
GPC3	AC007255.1	0.400856082	2.06E-17	postive
SLC11A2	AC007255.1	0.409939392	3.28E-18	postive
EMP3	ACTN1-AS1	0.402486689	1.49E-17	postive
PTGIR	ACTN1-AS1	0.437252932	9.24E-21	postive
RHOG	ACTN1-AS1	0.405109906	8.79E-18	postive
SCN1B	ACTN1-AS1	0.437442563	8.85E-21	postive
SPHK1	ACTN1-AS1	0.421762154	2.76E-19	postive
TIMP1	ACTN1-AS1	0.51022468	8.04E-29	postive
ABCA1	AC067750.1	0.425848995	1.15E-19	postive
CALCRL	AC067750.1	0.418421535	5.61E-19	postive
FZD5	AC067750.1	0.567691144	1.08E-36	postive
GPC3	AC067750.1	0.40848366	4.43E-18	postive
KCNMB2	AC067750.1	0.510184613	8.14E-29	postive
SLC11A2	AC010615.2	0.412230042	2.05E-18	postive
FZD5	AC063943.1	0.548664987	6.46E-34	postive
GPC3	AC063943.1	0.547712565	8.81E-34	postive
KCNMB2	AC063943.1	0.450833242	4.06E-22	postive
SLC11A2	AC063943.1	0.406697248	6.37E-18	postive
APLNR	AP003071.4	0.403926895	1.12E-17	postive

IL6	AP003071.4	0.442114224	3.07E-21	postive
ITGA5	AP003071.4	0.639993537	4.41E-49	postive
TNFAIP6	AP003071.4	0.447677632	8.50E-22	postive
ABCA1	AC087286.1	0.78372822	3.04E-87	postive
CYBB	AC087286.1	0.488993567	2.84E-26	postive
KIF1B	AC087286.1	0.422675193	2.27E-19	postive
TACR1	AC087286.1	0.455047355	1.50E-22	postive
TLR1	AC087286.1	0.501726895	8.85E-28	postive
ABCA1	AC010618.3	0.401112342	1.96E-17	postive
ACVR2A	MCCC1-AS1	0.460815829	3.73E-23	postive
RHOG	MCCC1-AS1	-0.418884986	5.09E-19	negative
BEST1	BNC2-AS1	0.542110956	5.32E-33	postive
CD14	BNC2-AS1	0.442733281	2.66E-21	postive
CMKLR1	BNC2-AS1	0.446859572	1.03E-21	postive
CSF1	BNC2-AS1	0.445413912	1.44E-21	postive
CYBB	BNC2-AS1	0.444340607	1.84E-21	postive
EMP3	BNC2-AS1	0.524784964	1.13E-30	postive
ITGA5	BNC2-AS1	0.403742785	1.16E-17	postive
PTGER2	BNC2-AS1	0.404351301	1.02E-17	postive
SCN1B	BNC2-AS1	0.513523104	3.11E-29	postive

TIMP1	BNC2-AS1	0.507839721	1.59E-28	postive
ABCA1	AC022893.1	0.422999826	2.12E-19	postive
CALCRL	AC022893.1	0.454685841	1.63E-22	postive
FZD5	AC022893.1	0.436522391	1.09E-20	postive
ITGA5	AC022893.1	0.43701925	9.74E-21	postive
TACR1	AC022893.1	0.400222831	2.34E-17	postive
FZD5	AC004083.1	0.482615594	1.53E-25	postive
LY6E	C8orf31	0.624850405	3.24E-46	postive
ABCA1	AC010525.1	0.486017293	6.26E-26	postive
ABCA1	AC068533.3	0.644900109	4.79E-50	postive
FZD5	AC068533.3	0.487736814	3.97E-26	postive
KCNMB2	AC068533.3	0.404976656	9.02E-18	postive
ABCA1	AL513327.1	0.669466802	3.77E-55	postive
KIF1B	AL513327.1	0.424491419	1.54E-19	postive
ABCA1	AC087286.2	0.738413981	1.68E-72	postive
CYBB	AC087286.2	0.445906022	1.28E-21	postive
KIF1B	AC087286.2	0.429455607	5.22E-20	postive
TLR1	AC087286.2	0.475571258	9.43E-25	postive
KCNMB2	CEP83-DT	0.407866428	5.02E-18	postive
ABCA1	MIR2052HG	0.446526894	1.11E-21	postive

FZD5	MIR2052HG	0.67626087	1.20E-56	postive
GPC3	MIR2052HG	0.446424264	1.14E-21	postive
KCNMB2	MIR2052HG	0.512917209	3.71E-29	postive
SLC11A2	MIR2052HG	0.417442836	6.90E-19	postive
ITGA5	AF001548.1	0.58383278	3.43E-39	postive
PDE4B	MIR99AHG	0.424112754	1.67E-19	postive
CD48	AC007384.1	0.624973796	3.07E-46	postive
EBI3	AC007384.1	0.474299011	1.30E-24	postive
GPR183	AC007384.1	0.480607015	2.58E-25	postive
KCNA3	AC007384.1	0.449687122	5.31E-22	postive
LCK	AC007384.1	0.543345698	3.59E-33	postive
LTA	AC007384.1	0.504777564	3.77E-28	postive
PTGIR	AC007384.1	0.42229372	2.46E-19	postive
SLAMF1	AC007384.1	0.518242738	7.87E-30	postive
TNFRSF1B	AC007384.1	0.407312847	5.62E-18	postive
MET	AC004943.2	0.418654454	5.34E-19	postive
ABCA1	AC011461.1	0.471032118	2.98E-24	postive
STAB1	AC011461.1	0.456594462	1.03E-22	postive
TACR1	AC011461.1	0.424346314	1.58E-19	postive
CSF3R	AC011453.1	0.591592222	1.92E-40	postive

SLC7A2	AC011453.1	0.414089946	1.39E-18	postive
SPHK1	LINC01357	0.445609752	1.37E-21	postive
ACVR2A	AC011477.2	0.440432118	4.50E-21	postive
ABCA1	AC025031.4	0.495213325	5.31E-27	postive
FZD5	AC025031.4	0.689524972	1.08E-59	postive
GPC3	AC025031.4	0.409153085	3.86E-18	postive
KCNMB2	AC025031.4	0.516871781	1.18E-29	postive
SLC11A2	AC025031.4	0.441450223	3.57E-21	postive
FZD5	SNHG14	0.542989594	4.02E-33	postive
ABCA1	AL031666.3	0.552474704	1.86E-34	postive
FZD5	AL031666.3	0.642853155	1.21E-49	postive
KCNMB2	AL031666.3	0.502355317	7.43E-28	postive
SLC11A2	AL031666.3	0.433060776	2.36E-20	postive
ABCA1	RMRP	0.662012552	1.50E-53	postive
CALCRL	RMRP	0.417928695	6.23E-19	postive
GPC3	RMRP	0.403118474	1.31E-17	postive
ABCA1	AC234775.2	0.55860289	2.42E-35	postive
FZD5	AC234775.2	0.412693371	1.86E-18	postive
ABCA1	LAMC1-AS1	0.565435958	2.36E-36	postive
FZD5	LAMC1-AS1	0.42262102	2.30E-19	postive

FZD5	AC022784.6	0.672627586	7.66E-56	postive
GPC3	AC022784.6	0.438633334	6.77E-21	postive
KCNMB2	AC022784.6	0.48325295	1.30E-25	postive
SLC11A2	AC022784.6	0.410869987	2.71E-18	postive
CLEC5A	AC099066.2	0.419901051	4.10E-19	postive
CSF1	AC099066.2	0.445301797	1.47E-21	postive
EMP3	AC099066.2	0.466774103	8.63E-24	postive
MMP14	AC099066.2	0.526524869	6.67E-31	postive
OSMR	AC099066.2	0.413970996	1.43E-18	postive
PLAUR	AC099066.2	0.419800624	4.19E-19	postive
RHOG	AC099066.2	0.405350186	8.37E-18	postive
SPHK1	AC099066.2	0.480881876	2.40E-25	postive
TNFAIP6	AC099066.2	0.462817746	2.29E-23	postive
PROK2	MEG3	0.562145982	7.29E-36	postive
VIP	MEG3	0.858116273	2.44E-121	postive
BEST1	LINC01943	0.497676932	2.71E-27	postive
C3AR1	LINC01943	0.641924626	1.85E-49	postive
C5AR1	LINC01943	0.56603153	1.93E-36	postive
CD14	LINC01943	0.508295846	1.39E-28	postive
CLEC5A	LINC01943	0.408723018	4.21E-18	postive

CMKLR1	LINC01943	0.517906168	8.68E-30	postive
CSF1	LINC01943	0.42930773	5.39E-20	postive
CXCL9	LINC01943	0.489219922	2.67E-26	postive
CXCR6	LINC01943	0.641892486	1.88E-49	postive
CYBB	LINC01943	0.624718399	3.43E-46	postive
EMP3	LINC01943	0.469332079	4.56E-24	postive
FPR1	LINC01943	0.510002086	8.57E-29	postive
GPR132	LINC01943	0.534337484	6.11E-32	postive
GPR183	LINC01943	0.573785939	1.28E-37	postive
IL10RA	LINC01943	0.659579361	4.88E-53	postive
IL15	LINC01943	0.458939261	5.87E-23	postive
IL15RA	LINC01943	0.533192104	8.71E-32	postive
IL18RAP	LINC01943	0.565412705	2.38E-36	postive
IL2RB	LINC01943	0.420298439	3.77E-19	postive
IL7R	LINC01943	0.477315158	6.04E-25	postive
IRF1	LINC01943	0.434434709	1.74E-20	postive
KCNA3	LINC01943	0.505519989	3.06E-28	postive
LCK	LINC01943	0.455464353	1.35E-22	postive
LCP2	LINC01943	0.698577104	7.19E-62	postive
MSR1	LINC01943	0.513576447	3.07E-29	postive

NLRP3	LINC01943	0.522175241	2.46E-30	postive
OSM	LINC01943	0.527509808	4.95E-31	postive
P2RX7	LINC01943	0.553724896	1.23E-34	postive
PDE4B	LINC01943	0.465416812	1.21E-23	postive
PIK3R5	LINC01943	0.612493024	5.44E-44	postive
PLAUR	LINC01943	0.457444782	8.42E-23	postive
PTAFR	LINC01943	0.51952953	5.39E-30	postive
PTGIR	LINC01943	0.529814457	2.46E-31	postive
RGS1	LINC01943	0.675063996	2.21E-56	postive
RHOG	LINC01943	0.462078015	2.74E-23	postive
SCN1B	LINC01943	0.512497719	4.19E-29	postive
SPHK1	LINC01943	0.40875447	4.19E-18	postive
TAPBP	LINC01943	0.402970791	1.35E-17	postive
TIMP1	LINC01943	0.423249772	2.01E-19	postive
TLR1	LINC01943	0.584507201	2.68E-39	postive
TNFAIP6	LINC01943	0.415392713	1.06E-18	postive
TNFRSF1B	LINC01943	0.615806657	1.41E-44	postive
TNFRSF9	LINC01943	0.478329153	4.65E-25	postive
ABCA1	LINC01146	0.452883342	2.50E-22	postive
CALCRL	LINC01146	0.461791523	2.94E-23	postive

FZD5	LINC01146	0.688332377	2.05E-59	postive
GPC3	LINC01146	0.550238028	3.87E-34	postive
SLC11A2	LINC01146	0.406505955	6.62E-18	postive
KCNMB2	AL161729.4	0.414335846	1.32E-18	postive
ABCA1	AC073130.2	0.667728582	8.99E-55	postive
FZD5	AC073130.2	0.473990577	1.41E-24	postive
C3AR1	LINC02084	0.448359761	7.25E-22	postive
CD14	LINC02084	0.403371649	1.25E-17	postive
CD48	LINC02084	0.43821176	7.44E-21	postive
CXCL9	LINC02084	0.619988172	2.50E-45	postive
CXCR6	LINC02084	0.646125533	2.73E-50	postive
GPR132	LINC02084	0.445245772	1.49E-21	postive
IL10RA	LINC02084	0.516212577	1.43E-29	postive
IL15RA	LINC02084	0.497505598	2.84E-27	postive
IL18RAP	LINC02084	0.531753618	1.36E-31	postive
IL2RB	LINC02084	0.462336451	2.57E-23	postive
KCNA3	LINC02084	0.495515566	4.89E-27	postive
LCK	LINC02084	0.515536981	1.74E-29	postive
LCP2	LINC02084	0.54145769	6.55E-33	postive
LTA	LINC02084	0.426446315	1.01E-19	postive

P2RX7	LINC02084	0.417245875	7.19E-19	postive
PIK3R5	LINC02084	0.430141575	4.49E-20	postive
PTAFR	LINC02084	0.51481131	2.14E-29	postive
RGS1	LINC02084	0.488749783	3.03E-26	postive
TNFRSF1B	LINC02084	0.529369317	2.82E-31	postive
TNFRSF9	LINC02084	0.497183946	3.10E-27	postive
ABCA1	AC096992.2	0.419396741	4.56E-19	postive
TNFSF15	HOTTIP	0.459279013	5.41E-23	postive
FZD5	AL592043.1	0.517317803	1.03E-29	postive
GABBR1	AC025165.1	0.453425703	2.20E-22	postive
RAF1	AC124276.2	0.462961415	2.21E-23	postive
ABCA1	AC073046.1	0.526208796	7.34E-31	postive
FZD5	AC073046.1	0.499219618	1.77E-27	postive
IFITM1	PICSAR	0.507929127	1.55E-28	postive
PLAUR	PICSAR	0.421513165	2.91E-19	postive
SPHK1	PICSAR	0.506410042	2.38E-28	postive
GPC3	PHKA2-AS1	0.444499059	1.77E-21	postive
CD40	AC135507.1	0.408622154	4.30E-18	postive
CD48	AC135507.1	0.438778014	6.55E-21	postive
EBI3	AC135507.1	0.443571831	2.20E-21	postive

SLAMF1	AC135507.1	0.493992491	7.40E-27	postive
ABCA1	LINC01126	0.435084127	1.50E-20	postive
FZD5	LINC01126	0.55147019	2.59E-34	postive
KCNMB2	LINC01126	0.406226925	7.01E-18	postive
SLC11A2	LINC01126	0.406545953	6.57E-18	postive
ABCA1	AP005057.1	0.545256506	1.95E-33	postive
FZD5	AP005057.1	0.573685945	1.33E-37	postive
CALCRL	AC002451.1	0.48605431	6.20E-26	postive
FZD5	AC002451.1	0.71279913	1.86E-65	postive
GPC3	AC002451.1	0.677931672	5.04E-57	postive
SLC11A2	AC002451.1	0.463917567	1.75E-23	postive
ABCA1	AC011465.1	0.509262951	1.06E-28	postive
ABCA1	AL008718.3	0.603079268	2.32E-42	postive
FZD5	AL008718.3	0.479118213	3.79E-25	postive
EIF2AK2	AL445490.1	0.536431695	3.19E-32	postive
ABCA1	AC139887.2	0.583218175	4.30E-39	postive
FZD5	AC139887.2	0.550407386	3.66E-34	postive
KCNMB2	AC139887.2	0.43638029	1.12E-20	postive
KIF1B	AC139887.2	0.415097153	1.13E-18	postive
SLC11A2	AC139887.2	0.448557869	6.92E-22	postive

ABCA1	LINC00641	0.480697918	2.52E-25	postive
FZD5	LINC00641	0.409779961	3.39E-18	postive
KCNMB2	LINC00641	0.488749367	3.03E-26	postive
KIF1B	LINC00641	0.429366883	5.32E-20	postive
FZD5	LINC01675	0.689216179	1.27E-59	postive
GPC3	LINC01675	0.422978506	2.13E-19	postive
KCNMB2	LINC01675	0.480449423	2.69E-25	postive
SLC11A2	LINC01675	0.416198564	8.96E-19	postive
C5AR1	AL356417.2	0.494910432	5.77E-27	postive
FPR1	AL356417.2	0.454733678	1.61E-22	postive
PTGIR	AL356417.2	0.53046446	2.01E-31	postive
SCN1B	AL356417.2	0.414400585	1.30E-18	postive
TIMP1	AL356417.2	0.451384266	3.57E-22	postive
TNFAIP6	AL356417.2	0.479929455	3.08E-25	postive
ABCA1	LINC01409	0.583575453	3.77E-39	postive
FZD5	LINC01409	0.566544672	1.61E-36	postive
KCNMB2	LINC01409	0.445097468	1.55E-21	postive
SLC11A2	LINC01409	0.430140058	4.49E-20	postive
FZD5	LINC01376	0.42105498	3.21E-19	postive
ABCA1	PRC1-AS1	0.599738526	8.54E-42	postive

ABCA1	AC100821.2	0.517973522	8.51E-30	postive
FZD5	AC100821.2	0.537682782	2.15E-32	postive
ABCA1	AC067852.3	0.758696673	1.07E-78	postive
CYBB	AC067852.3	0.436860819	1.01E-20	postive
KIF1B	AC067852.3	0.506882434	2.08E-28	postive
STAB1	AC067852.3	0.438233131	7.41E-21	postive
TACR1	AC067852.3	0.492982306	9.73E-27	postive
TLR1	AC067852.3	0.440356887	4.58E-21	postive
ABCA1	AC090559.1	0.465513107	1.18E-23	postive
APLNR	AC090559.1	0.43247947	2.68E-20	postive
AXL	AC090559.1	0.440106809	4.85E-21	postive
BEST1	AC090559.1	0.634213717	5.71E-48	postive
C3AR1	AC090559.1	0.757428572	2.73E-78	postive
C5AR1	AC090559.1	0.617476649	7.08E-45	postive
CD14	AC090559.1	0.562760167	5.91E-36	postive
CMKLR1	AC090559.1	0.686520411	5.44E-59	postive
CSF1	AC090559.1	0.666482284	1.67E-54	postive
CXCL9	AC090559.1	0.515977947	1.53E-29	postive
CXCR6	AC090559.1	0.449469081	5.59E-22	postive
CYBB	AC090559.1	0.842512911	9.45E-113	postive

ADGRE1	AC090559.1	0.476962332	6.61E-25	postive
FPR1	AC090559.1	0.597934574	1.71E-41	postive
GPR132	AC090559.1	0.526971837	5.83E-31	postive
GPR183	AC090559.1	0.554014514	1.12E-34	postive
IL10	AC090559.1	0.406769263	6.28E-18	postive
IL10RA	AC090559.1	0.840579941	9.41E-112	postive
IL2RB	AC090559.1	0.461793808	2.94E-23	postive
IL7R	AC090559.1	0.508722912	1.23E-28	postive
ITGB3	AC090559.1	0.493301954	8.93E-27	postive
KCNA3	AC090559.1	0.598266234	1.51E-41	postive
LCK	AC090559.1	0.422207328	2.51E-19	postive
LCP2	AC090559.1	0.796742453	3.75E-92	postive
LPAR1	AC090559.1	0.433908417	1.95E-20	postive
LYN	AC090559.1	0.422108523	2.56E-19	postive
MEFV	AC090559.1	0.431211711	3.55E-20	postive
MSR1	AC090559.1	0.673668807	4.51E-56	postive
NLRP3	AC090559.1	0.663928327	5.88E-54	postive
OSM	AC090559.1	0.472789397	1.91E-24	postive
OSMR	AC090559.1	0.506945304	2.05E-28	postive
P2RX7	AC090559.1	0.753935291	3.46E-77	postive

PDE4B	AC090559.1	0.573625353	1.36E-37	postive
PIK3R5	AC090559.1	0.861380153	2.89E-123	postive
PTAFR	AC090559.1	0.520881561	3.61E-30	postive
PTGER2	AC090559.1	0.411651533	2.31E-18	postive
RGS1	AC090559.1	0.559075502	2.06E-35	postive
SCARF1	AC090559.1	0.489003766	2.83E-26	postive
SCN1B	AC090559.1	0.472612826	2.00E-24	postive
STAB1	AC090559.1	0.665652828	2.52E-54	postive
TACR1	AC090559.1	0.577577255	3.32E-38	postive
TLR1	AC090559.1	0.748265597	1.95E-75	postive
TLR2	AC090559.1	0.486335702	5.75E-26	postive
TNFAIP6	AC090559.1	0.45561972	1.30E-22	postive
TNFRSF1B	AC090559.1	0.63923007	6.20E-49	postive
TNFRSF9	AC090559.1	0.566560799	1.60E-36	postive
ABCA1	LINC00894	0.441664771	3.40E-21	postive
FZD5	LINC00894	0.443170677	2.41E-21	postive
FZD5	AL157400.4	0.441917458	3.21E-21	postive
CD40	AC017104.1	0.429325297	5.37E-20	postive
CD48	AC017104.1	0.684382899	1.70E-58	postive
EBI3	AC017104.1	0.607898102	3.45E-43	postive

GP1BA	AC017104.1	0.469103603	4.83E-24	postive
LCK	AC017104.1	0.598982787	1.14E-41	postive
LTA	AC017104.1	0.591780407	1.79E-40	postive
PTGIR	AC017104.1	0.503071554	6.08E-28	postive
SLAMF1	AC017104.1	0.623200158	6.51E-46	postive
ABCA1	AP000829.1	0.489177295	2.70E-26	postive
KCNMB2	LINC01571	0.658983505	6.50E-53	postive
EMP3	AC112715.1	0.427978156	7.21E-20	postive
SCN1B	AC112715.1	0.52346213	1.67E-30	postive
ABCA1	AC245884.10	0.515269327	1.88E-29	postive
FZD5	AC245884.10	0.550539234	3.51E-34	postive
KCNMB2	AC245884.10	0.458917945	5.90E-23	postive
STAB1	AC114489.1	0.404867725	9.23E-18	postive
KCNMB2	Z97989.1	0.665132812	3.25E-54	postive
BEST1	AC090825.1	0.475392891	9.87E-25	postive
C3AR1	AC090825.1	0.4223671	2.42E-19	postive
CD48	AC090825.1	0.451224304	3.70E-22	postive
CYBB	AC090825.1	0.426350764	1.03E-19	postive
GP1BA	AC090825.1	0.434548377	1.69E-20	postive
GPR183	AC090825.1	0.547762919	8.66E-34	postive

IL10RA	AC090825.1	0.565420931	2.38E-36	postive
ITGA5	AC090825.1	0.452911718	2.49E-22	postive
KCNA3	AC090825.1	0.522558724	2.19E-30	postive
LCK	AC090825.1	0.433648608	2.07E-20	postive
LCP2	AC090825.1	0.459507086	5.12E-23	postive
LTA	AC090825.1	0.511295404	5.92E-29	postive
P2RX7	AC090825.1	0.42072357	3.44E-19	postive
PDE4B	AC090825.1	0.50836501	1.37E-28	postive
PIK3R5	AC090825.1	0.515242888	1.89E-29	postive
RGS1	AC090825.1	0.427403355	8.17E-20	postive
SCN1B	AC090825.1	0.40799153	4.89E-18	postive
TLR1	AC090825.1	0.42507638	1.35E-19	postive
TNFRSF1B	AC090825.1	0.454039171	1.90E-22	postive
FZD5	AC024361.3	0.638932867	7.08E-49	postive
KCNMB2	AC024361.3	0.464053629	1.69E-23	postive
SLC11A2	AC024361.3	0.456051162	1.18E-22	postive
CALCRL	HID1-AS1	0.636798423	1.83E-48	postive
FZD5	HID1-AS1	0.586879147	1.12E-39	postive
GPC3	HID1-AS1	0.654629266	5.20E-52	postive
IL1R1	HID1-AS1	0.421557299	2.88E-19	postive

SCARF1	HID1-AS1	0.435540755	1.36E-20	postive
ITGA5	AC005180.2	0.659474092	5.13E-53	postive
ABCA1	AL138756.1	0.460118226	4.42E-23	postive
CALCRL	AL138756.1	0.415463397	1.05E-18	postive
FZD5	AL138756.1	0.684100472	1.98E-58	postive
GPC3	AL138756.1	0.5764463	4.97E-38	postive
SLC11A2	AL138756.1	0.5121209	4.67E-29	postive
ABCA1	AC090579.1	0.440025486	4.94E-21	postive
FZD5	AC090579.1	0.581968699	6.78E-39	postive
SLC11A2	AC090579.1	0.421069195	3.20E-19	postive
ABCA1	AC024075.3	0.426426286	1.01E-19	postive
FZD5	AC024075.3	0.430957992	3.75E-20	postive
SLC11A2	AC024075.3	0.41774237	6.48E-19	postive
P2RX4	LBX2-AS1	0.444071901	1.96E-21	postive
LY6E	LINC02643	0.512059555	4.75E-29	postive
ABCA1	AC015923.1	0.518258826	7.83E-30	postive
FZD5	AC015923.1	0.658752983	7.27E-53	postive
KCNMB2	AC015923.1	0.513783569	2.89E-29	postive
SLC11A2	AC015923.1	0.420176505	3.87E-19	postive
MEP1A	GATA6-AS1	0.578470481	2.41E-38	postive

ABCA1	AL110115.1	0.627897903	8.84E-47	postive
APLNR	AP001189.3	0.493826836	7.74E-27	postive
CALCRL	AP001189.3	0.518047311	8.33E-30	postive
IL1R1	AP001189.3	0.502240426	7.67E-28	postive
ITGA5	AP001189.3	0.468868761	5.12E-24	postive
PTGER2	AP001189.3	0.42717039	8.60E-20	postive
TNFAIP6	AP001189.3	0.476148841	8.14E-25	postive
ABCA1	AC068790.6	0.632556417	1.18E-47	postive
FZD5	AC068790.6	0.461163039	3.43E-23	postive
KCNMB2	AC068790.6	0.411809123	2.23E-18	postive
ABCA1	OPA1-AS1	0.693200702	1.44E-60	postive
FZD5	OPA1-AS1	0.488630861	3.13E-26	postive
KCNMB2	OPA1-AS1	0.409445214	3.63E-18	postive
ABCA1	AC093620.1	0.592109773	1.58E-40	postive
STAB1	AC093620.1	0.481840759	1.87E-25	postive
TACR1	AC093620.1	0.466937313	8.29E-24	postive
TLR1	AC093620.1	0.415825051	9.69E-19	postive
ABCA1	AL158212.3	0.438282791	7.32E-21	postive
KCNMB2	AL158212.3	0.493356528	8.80E-27	postive
KIF1B	AL158212.3	0.533523052	7.87E-32	postive

ABCA1	AC124312.2	0.491531664	1.44E-26	postive
CALCRL	AC124312.2	0.507733517	1.64E-28	postive
FZD5	AC124312.2	0.740111219	5.36E-73	postive
GPC3	AC124312.2	0.568739949	7.53E-37	postive
KCNMB2	AC124312.2	0.417050948	7.49E-19	postive
SLC11A2	AC124312.2	0.52238098	2.31E-30	postive
ABCA1	AC008443.2	0.455027768	1.50E-22	postive
CYBB	AC008443.2	0.412969477	1.76E-18	postive
KIF1B	AC008443.2	0.417779499	6.42E-19	postive
PIK3R5	AC008443.2	0.402191906	1.58E-17	postive
STAB1	AC008443.2	0.614782037	2.14E-44	postive
TACR1	AC008443.2	0.483494131	1.22E-25	postive
C3AR1	LINC00882	0.487018471	4.80E-26	postive
CCL24	LINC00882	0.496630963	3.61E-27	postive
CD14	LINC00882	0.421098902	3.18E-19	postive
CMKLR1	LINC00882	0.483124489	1.34E-25	postive
CSF1	LINC00882	0.458999297	5.79E-23	postive
STAB1	LINC00882	0.423344774	1.97E-19	postive
TLR1	LINC00882	0.420003332	4.01E-19	postive
ABCA1	LINC00630	0.640590116	3.37E-49	postive

EIF2AK2	LINC00630	0.489507281	2.48E-26	postive
FZD5	LINC00630	0.497193443	3.09E-27	postive
KIF1B	LINC00630	0.512565525	4.11E-29	postive
SLC11A2	LINC00630	0.458705697	6.22E-23	postive
ABCA1	AC024270.3	0.593020614	1.12E-40	postive
KCNMB2	AC024270.3	0.401125127	1.95E-17	postive
ABCA1	AC107081.1	0.435405645	1.40E-20	postive
CD55	AC104435.2	0.434845738	1.58E-20	postive
P2RX4	AC104435.2	0.410915995	2.69E-18	postive
CCL24	LINC01705	0.420968452	3.27E-19	postive
MMP14	LINC01705	0.504886108	3.66E-28	postive
PLAUR	LINC01705	0.404483904	9.97E-18	postive
SPHK1	LINC01705	0.416055186	9.23E-19	postive
ABCA1	PTPRK-AS1	0.5265863	6.55E-31	postive
FZD5	AL138921.1	0.437888672	8.00E-21	postive
MMP14	LINC01929	0.434531336	1.70E-20	postive
APLNR	BX322562.1	0.459737964	4.84E-23	postive
EMP3	BX322562.1	0.426432735	1.01E-19	postive
ITGA5	BX322562.1	0.423999335	1.71E-19	postive
MMP14	BX322562.1	0.401555986	1.79E-17	postive

PTGIR	BX322562.1	0.435816148	1.28E-20	postive
SCN1B	BX322562.1	0.507444647	1.78E-28	postive
TIMP1	BX322562.1	0.456587052	1.03E-22	postive
TNFAIP6	BX322562.1	0.42213213	2.55E-19	postive
MET	AC034223.2	0.410787335	2.76E-18	postive
CCL24	AC112721.2	0.86175706	1.72E-123	postive
CMKLR1	AC112721.2	0.406980246	6.01E-18	postive
FZD5	AC009955.4	0.424501562	1.53E-19	postive
ABCA1	AP006621.2	0.422363619	2.43E-19	postive
SELL	LINC02527	0.597162839	2.31E-41	postive
PTPRE	AC004034.1	0.509304668	1.05E-28	postive
FZD5	AL135905.1	0.420717257	3.45E-19	postive
GPC3	AL135905.1	0.401849802	1.69E-17	postive
ABCA1	AC019080.5	0.545688191	1.69E-33	postive
CYBB	AC019080.5	0.419330634	4.63E-19	postive
TLR1	AC019080.5	0.432889744	2.45E-20	postive
ABCA1	AC105036.3	0.513920294	2.78E-29	postive
ABCA1	LINC02109	0.401847912	1.69E-17	postive
CALCRL	LINC02109	0.440333702	4.60E-21	postive
FZD5	LINC02109	0.731368731	1.75E-70	postive

GPC3	LINC02109	0.52137743	3.12E-30	postive
KCNMB2	LINC02109	0.474769572	1.16E-24	postive
SLC11A2	LINC02109	0.442650535	2.71E-21	postive
ACVR2A	HDAC2-AS2	0.467573955	7.07E-24	postive
EBI3	AC004832.6	0.42822179	6.84E-20	postive
SLAMF1	AC004832.6	0.522696808	2.10E-30	postive
ABCA1	EGOT	0.440201904	4.74E-21	postive
ABCA1	AC012645.2	0.403727825	1.16E-17	postive
FZD5	AC012645.2	0.422950417	2.14E-19	postive
SLC11A2	AC012645.2	0.405980764	7.37E-18	postive
ABCA1	AGAP1-IT1	0.616965091	8.74E-45	postive
CYBB	AGAP1-IT1	0.419767615	4.22E-19	postive
STAB1	AGAP1-IT1	0.4300807	4.55E-20	postive
TACR1	AGAP1-IT1	0.477038339	6.48E-25	postive
ABCA1	AC008115.3	0.527603411	4.82E-31	postive
CD48	CEROX1	0.419454924	4.51E-19	postive
GP1BA	CEROX1	0.425260688	1.30E-19	postive
LCK	CEROX1	0.406496441	6.63E-18	postive
LTA	CEROX1	0.513621616	3.03E-29	postive
PDE4B	CEROX1	0.419591792	4.38E-19	postive

TNFRSF9	CEROX1	0.437601051	8.54E-21	postive
ABCA1	NPTN-IT1	0.61743621	7.20E-45	postive
KIF1B	NPTN-IT1	0.411806561	2.24E-18	postive
FZD5	AL121574.1	0.565139858	2.62E-36	postive
GPC3	AL121574.1	0.549082202	5.64E-34	postive
SLC11A2	AL121574.1	0.463985217	1.72E-23	postive
ITGA5	AP003071.3	0.592153146	1.55E-40	postive
ABCA1	AC004884.2	0.674147439	3.53E-56	postive
FZD5	AC004884.2	0.481921222	1.83E-25	postive
KCNMB2	AC004884.2	0.411271629	2.50E-18	postive
CYBB	LINC01915	0.536064783	3.57E-32	postive
ADGRE1	LINC01915	0.548997179	5.80E-34	postive
PIK3R5	LINC01915	0.49823727	2.32E-27	postive
STAB1	LINC01915	0.628883558	5.79E-47	postive
TACR1	LINC01915	0.676073817	1.32E-56	postive
TLR1	LINC01915	0.463175532	2.10E-23	postive
VIP	LINC01915	0.485986009	6.31E-26	postive
ACVR2A	AL590652.1	0.434336513	1.77E-20	postive
RHOG	AL590652.1	-0.432183994	2.86E-20	negative
ABCA1	AC020978.7	0.535025715	4.94E-32	postive

ABCA1	AC009704.2	0.58390963	3.33E-39	postive
FZD5	AC009704.2	0.41085326	2.72E-18	postive
ABCA1	AC006460.1	0.477323421	6.02E-25	postive
AHR	AC006460.1	0.430137347	4.49E-20	postive
ABCA1	AC073529.1	0.468370123	5.80E-24	postive
FZD5	AC073529.1	0.420406767	3.68E-19	postive
KIF1B	AC073529.1	0.400590546	2.17E-17	postive
SLC11A2	AC073529.1	0.412461061	1.95E-18	postive
CALCRL	AC005828.4	0.410814489	2.74E-18	postive
FZD5	AC005828.4	0.696078403	2.92E-61	postive
GPC3	AC005828.4	0.490966935	1.68E-26	postive
KCNMB2	AC005828.4	0.482531624	1.56E-25	postive
SLC11A2	AC005828.4	0.44533062	1.46E-21	postive
ABCA1	AL445309.1	0.554409241	9.80E-35	postive
FZD5	AL445309.1	0.561962171	7.76E-36	postive
KCNMB2	AL445309.1	0.470762783	3.19E-24	postive
SLC11A2	AL445309.1	0.423388712	1.95E-19	postive
C5AR1	LINC02884	0.401097197	1.97E-17	postive
FPR1	LINC02884	0.426255634	1.05E-19	postive
SCN1B	LINC02884	0.436376433	1.12E-20	postive

BEST1	AC008972.2	0.406327911	6.87E-18	postive
C3AR1	AC008972.2	0.478697075	4.23E-25	postive
CD14	AC008972.2	0.409090222	3.91E-18	postive
CMKLR1	AC008972.2	0.446415854	1.14E-21	postive
CYBB	AC008972.2	0.408754565	4.19E-18	postive
IL10RA	AC008972.2	0.46471923	1.43E-23	postive
LCP2	AC008972.2	0.427943992	7.26E-20	postive
PIK3R5	AC008972.2	0.447731867	8.39E-22	postive
OSMR	DANCR	-0.400674689	2.14E-17	negative
MEP1A	AC124798.1	0.492200111	1.20E-26	postive
ABCA1	AC124312.3	0.48012753	2.92E-25	postive
CALCRL	AC124312.3	0.594942951	5.39E-41	postive
FZD5	AC124312.3	0.751493685	1.99E-76	postive
GPC3	AC124312.3	0.671651627	1.26E-55	postive
SLC11A2	AC124312.3	0.529675557	2.56E-31	postive
ABCA1	FTX	0.708210651	2.82E-64	postive
FZD5	FTX	0.470273435	3.61E-24	postive
KIF1B	FTX	0.413228415	1.67E-18	postive
ABCA1	AC015849.4	0.74539287	1.45E-74	postive
FZD5	AC015849.4	0.421972037	2.64E-19	postive

KIF1B	AC015849.4	0.402770219	1.41E-17	postive
TACR1	AC015849.4	0.462949911	2.21E-23	postive
ABCA1	AC018410.1	0.593333477	9.94E-41	postive
ABCA1	DIP2C-AS1	0.575725052	6.43E-38	postive
FZD5	DIP2C-AS1	0.461716425	2.99E-23	postive
KCNMB2	DIP2C-AS1	0.400234592	2.33E-17	postive
ABCA1	AC007292.2	0.412112049	2.10E-18	postive
ABCA1	PHC2-AS1	0.466698376	8.79E-24	postive
FZD5	PHC2-AS1	0.402457074	1.50E-17	postive
KCNMB2	PHC2-AS1	0.464997247	1.34E-23	postive
SLC4A4	AC079466.1	0.411649961	2.31E-18	postive
ABCA1	AL353804.2	0.725862172	5.99E-69	postive
FZD5	AL353804.2	0.466093684	1.02E-23	postive
TACR1	AL353804.2	0.400975709	2.01E-17	postive
TLR1	AL353804.2	0.408001862	4.88E-18	postive
NOD2	LINC01527	0.438672465	6.71E-21	postive
ABCA1	LRRC8C-DT	0.574959324	8.45E-38	postive
CYBB	LRRC8C-DT	0.484673598	8.92E-26	postive
IL10RA	LRRC8C-DT	0.44757044	8.71E-22	postive
KCNMB2	LRRC8C-DT	0.432560824	2.63E-20	postive

PIK3R5	LRRC8C-DT	0.517647685	9.37E-30	postive
STAB1	LRRC8C-DT	0.538519825	1.66E-32	postive
TACR1	LRRC8C-DT	0.552275681	1.98E-34	postive
TLR1	LRRC8C-DT	0.455550095	1.33E-22	postive
ABCA1	AC211476.2	0.59009176	3.37E-40	postive
FZD5	AC211476.2	0.416482559	8.44E-19	postive
KCNMB2	AC211476.2	0.429948038	4.68E-20	postive
ABCA1	AC129507.1	0.643725738	8.18E-50	postive
BEST1	AC129507.1	0.435463986	1.38E-20	postive
CALCRL	AC129507.1	0.437884891	8.01E-21	postive
CYBB	AC129507.1	0.484281879	9.89E-26	postive
FZD5	AC129507.1	0.470133007	3.73E-24	postive
IL10RA	AC129507.1	0.45627304	1.12E-22	postive
PIK3R5	AC129507.1	0.50765721	1.67E-28	postive
SCARF1	AC129507.1	0.448250115	7.44E-22	postive
STAB1	AC129507.1	0.548432915	6.97E-34	postive
TACR1	AC129507.1	0.668737312	5.43E-55	postive
TLR1	AC129507.1	0.423665691	1.83E-19	postive
IL18	LINC02762	0.4149524	1.16E-18	postive
ABCA1	AC008731.1	0.663523979	7.17E-54	postive

AHR	AC008731.1	0.413630253	1.53E-18	postive
KIF1B	AC008731.1	0.401409146	1.85E-17	postive
ABCA1	LINC00456	0.55714138	3.95E-35	postive
CALCRL	LINC00456	0.537974102	1.97E-32	postive
FZD5	LINC00456	0.707844554	3.50E-64	postive
GPC3	LINC00456	0.604536339	1.31E-42	postive
SLC11A2	LINC00456	0.566499961	1.64E-36	postive
ABCA1	LINC01876	0.456221518	1.13E-22	postive
FZD5	LINC01876	0.614990384	1.97E-44	postive
KCNMB2	LINC01876	0.447035801	9.87E-22	postive
SLC11A2	LINC01876	0.471206979	2.85E-24	postive
AXL	VIM-AS1	0.40749828	5.41E-18	postive
C3AR1	VIM-AS1	0.496150803	4.11E-27	postive
CSF1	VIM-AS1	0.42704083	8.84E-20	postive
EMP3	VIM-AS1	0.423084636	2.08E-19	postive
GPR132	VIM-AS1	0.501613724	9.13E-28	postive
GPR183	VIM-AS1	0.407484604	5.43E-18	postive
IL10RA	VIM-AS1	0.47259887	2.01E-24	postive
IL15	VIM-AS1	0.414497474	1.28E-18	postive
IL15RA	VIM-AS1	0.420221868	3.83E-19	postive

IL18RAP	VIM-AS1	0.42834072	6.66E-20	postive
LCP2	VIM-AS1	0.527996575	4.27E-31	postive
NLRP3	VIM-AS1	0.429188737	5.53E-20	postive
P2RX7	VIM-AS1	0.416109388	9.13E-19	postive
PDE4B	VIM-AS1	0.433429281	2.17E-20	postive
PIK3R5	VIM-AS1	0.456996347	9.38E-23	postive
PLAUR	VIM-AS1	0.42653093	9.88E-20	postive
PTAFR	VIM-AS1	0.424262444	1.61E-19	postive
RGS1	VIM-AS1	0.441236825	3.75E-21	postive
RHOG	VIM-AS1	0.414882121	1.18E-18	postive
TNFRSF1B	VIM-AS1	0.415097219	1.13E-18	postive
TNFRSF9	VIM-AS1	0.446503157	1.12E-21	postive
ABCA1	AC104564.5	0.650732924	3.25E-51	postive
ABCA1	AC007319.1	0.574397815	1.03E-37	postive
TLR1	AC007319.1	0.525325854	9.58E-31	postive
ABCA1	KLF7-IT1	0.741144283	2.66E-73	postive
CYBB	KLF7-IT1	0.501502071	9.42E-28	postive
KIF1B	KLF7-IT1	0.428114011	7.00E-20	postive
TACR1	KLF7-IT1	0.403539635	1.21E-17	postive
TLR1	KLF7-IT1	0.535514572	4.24E-32	postive

ABCA1	TGFB2-AS1	0.522942525	1.96E-30	postive
CYBB	TGFB2-AS1	0.61506751	1.91E-44	postive
IL10RA	TGFB2-AS1	0.462801721	2.30E-23	postive
MEFV	TGFB2-AS1	0.42302475	2.11E-19	postive
NLRP3	TGFB2-AS1	0.517151777	1.08E-29	postive
PIK3R5	TGFB2-AS1	0.592068383	1.60E-40	postive
STAB1	TGFB2-AS1	0.740925693	3.09E-73	postive
TACR1	TGFB2-AS1	0.595576261	4.23E-41	postive
TLR1	TGFB2-AS1	0.558339556	2.64E-35	postive
KCNMB2	AL445228.2	0.496333204	3.91E-27	postive
ABCA1	AL513327.2	0.596736966	2.72E-41	postive
FZD5	AL513327.2	0.472251757	2.19E-24	postive
SLC11A2	AL513327.2	0.430069072	4.56E-20	postive
ABCA1	AC006270.1	0.495475881	4.95E-27	postive
FZD5	AC006270.1	0.666300758	1.83E-54	postive
KCNMB2	AC006270.1	0.512890036	3.74E-29	postive
SLC11A2	AC006270.1	0.422106596	2.56E-19	postive
FZD5	LINC02820	0.429914409	4.72E-20	postive
OSMR	IQCH-AS1	-0.426308853	1.04E-19	negative
PLAUR	IQCH-AS1	-0.44686302	1.03E-21	negative

RHOG	IQCH-AS1	-0.444446443	1.80E-21	negative
SPHK1	IQCH-AS1	-0.434780713	1.61E-20	negative
ABCA1	AC103769.1	0.561563941	8.88E-36	postive
FZD5	AC103769.1	0.422855252	2.18E-19	postive
FZD5	AC104126.1	0.405770518	7.69E-18	postive
ABCA1	AL023755.1	0.429084868	5.66E-20	postive
ABCA1	AC097641.2	0.447725999	8.40E-22	postive
FZD5	AC097641.2	0.592050206	1.61E-40	postive
GPC3	AC097641.2	0.421114742	3.17E-19	postive
KCNMB2	AC097641.2	0.417899197	6.26E-19	postive
SLC11A2	AC097641.2	0.460434645	4.09E-23	postive
AXL	AC131097.3	0.420498418	3.61E-19	postive
BEST1	AC131097.3	0.553651286	1.26E-34	postive
C3AR1	AC131097.3	0.471217625	2.84E-24	postive
C5AR1	AC131097.3	0.443487338	2.24E-21	postive
CCL2	AC131097.3	0.453893375	1.97E-22	postive
CMKLR1	AC131097.3	0.447708741	8.44E-22	postive
CSF1	AC131097.3	0.407693373	5.20E-18	postive
CYBB	AC131097.3	0.475130723	1.06E-24	postive
ADGRE1	AC131097.3	0.420997247	3.25E-19	postive

IL10RA	AC131097.3	0.561148629	1.02E-35	postive
ITGA5	AC131097.3	0.441376641	3.63E-21	postive
ITGB3	AC131097.3	0.498591981	2.11E-27	postive
LCP2	AC131097.3	0.459086662	5.67E-23	postive
MSR1	AC131097.3	0.417245633	7.19E-19	postive
NLRP3	AC131097.3	0.491144104	1.60E-26	postive
P2RX7	AC131097.3	0.520614435	3.91E-30	postive
PDE4B	AC131097.3	0.449549732	5.49E-22	postive
PIK3R5	AC131097.3	0.58005065	1.36E-38	postive
SCARF1	AC131097.3	0.469672424	4.19E-24	postive
SCN1B	AC131097.3	0.478814489	4.10E-25	postive
STAB1	AC131097.3	0.45560405	1.31E-22	postive
TACR1	AC131097.3	0.413933879	1.44E-18	postive
TLR1	AC131097.3	0.401088676	1.97E-17	postive
TNFRSF1B	AC131097.3	0.466942588	8.28E-24	postive
TNFRSF9	AC131097.3	0.406322567	6.87E-18	postive
ABCA1	NECTIN4-AS1	0.430139947	4.49E-20	postive
FZD5	AC108704.1	0.438189218	7.48E-21	postive
KCNMB2	AC108704.1	0.504305825	4.30E-28	postive
NMUR1	AC008050.1	0.509457498	1.00E-28	postive

STAB1	AC008050.1	0.408740797	4.20E-18	postive
TACR1	AC008050.1	0.415065767	1.14E-18	postive
ABCA1	AC005391.1	0.588611545	5.86E-40	postive
FZD5	AC005391.1	0.507944361	1.54E-28	postive
KCNMB2	AC005391.1	0.412021222	2.14E-18	postive
SLC11A2	AC005391.1	0.404795675	9.36E-18	postive
GABBR1	AL662797.1	0.406318108	6.88E-18	postive
ABCA1	LIPC-AS1	0.426212062	1.06E-19	postive
FZD5	LIPC-AS1	0.692367501	2.28E-60	postive
GPC3	LIPC-AS1	0.411681818	2.29E-18	postive
KCNMB2	LIPC-AS1	0.501581158	9.21E-28	postive
SLC11A2	LIPC-AS1	0.418111738	5.99E-19	postive
FZD5	AC007881.3	0.458291773	6.87E-23	postive
GPC3	AC007881.3	0.403160941	1.30E-17	postive
ABCA1	BMS1P4	0.508548205	1.30E-28	postive
FZD5	BMS1P4	0.479089668	3.82E-25	postive
ABCA1	AP005432.1	0.440832988	4.11E-21	postive
FZD5	AP005432.1	0.673455036	5.03E-56	postive
KCNMB2	AP005432.1	0.513643971	3.01E-29	postive
SLC11A2	AP005432.1	0.414992057	1.15E-18	postive

APLNR	PCAT19	0.63819725	9.83E-49	postive
C5AR1	PCAT19	0.433782125	2.01E-20	postive
CALCRL	PCAT19	0.455998664	1.19E-22	postive
CCL2	PCAT19	0.461482592	3.17E-23	postive
FPR1	PCAT19	0.463498919	1.94E-23	postive
GPR183	PCAT19	0.402872216	1.38E-17	postive
IL6	PCAT19	0.468157466	6.12E-24	postive
ITGA5	PCAT19	0.438435887	7.08E-21	postive
PDE4B	PCAT19	0.439112413	6.07E-21	postive
PTGER2	PCAT19	0.489416437	2.54E-26	postive
PTGIR	PCAT19	0.51134198	5.84E-29	postive
SCARF1	PCAT19	0.538499875	1.67E-32	postive
TIMP1	PCAT19	0.438575678	6.86E-21	postive
EMP3	AC018529.1	0.45270517	2.61E-22	postive
NMUR1	AC018529.1	0.775654797	2.29E-84	postive
TIMP1	AC018529.1	0.422735165	2.24E-19	postive
ABCA1	AC087289.1	0.434295459	1.79E-20	postive
FZD5	AC087289.1	0.472303551	2.16E-24	postive
SLC11A2	AC087289.1	0.408828073	4.12E-18	postive
FZD5	FGF12-AS3	0.400220265	2.34E-17	postive

FZD5	ARHGEF2-AS2	0.527836726	4.49E-31	postive
KCNMB2	ARHGEF2-AS2	0.417499506	6.82E-19	postive
ABCA1	AP001628.1	0.482483335	1.58E-25	postive
FZD5	AP001628.1	0.606238607	6.68E-43	postive
KCNMB2	AP001628.1	0.493553696	8.34E-27	postive
SLC11A2	AP001628.1	0.43571688	1.30E-20	postive
PTPRE	LINC01411	0.504466195	4.11E-28	postive
FPR1	DIRC1	0.436943226	9.90E-21	postive
MMP14	DIRC1	0.46995466	3.91E-24	postive
TNFAIP6	DIRC1	0.581260341	8.77E-39	postive
OSMR	AL390294.1	-0.401042257	1.99E-17	negative
ABCA1	NARF-IT1	0.413678511	1.52E-18	postive
ABCA1	AL731563.3	0.430871399	3.82E-20	postive
ABCA1	AC005479.1	0.529296447	2.88E-31	postive
KIF1B	AC005479.1	0.406505636	6.62E-18	postive
FZD5	AC016571.1	0.535753659	3.94E-32	postive
ABCA1	AC104041.1	0.427427125	8.13E-20	postive
FZD5	AC104041.1	0.41557509	1.02E-18	postive
ABCA1	AC063919.1	0.412893972	1.78E-18	postive
CALCRL	AC063919.1	0.58936227	4.43E-40	postive

FZD5	AC063919.1	0.71130055	4.55E-65	postive
GPC3	AC063919.1	0.916608216	3.85E-166	postive
SLC11A2	AC063919.1	0.496323147	3.93E-27	postive
BEST1	LINC01352	0.4018169	1.70E-17	postive
CALCRL	LINC01352	0.429712048	4.93E-20	postive
GP1BA	LINC01352	0.411714425	2.28E-18	postive
IL1R1	LINC01352	0.430759071	3.92E-20	postive
ABCA1	AC107419.1	0.46546204	1.19E-23	postive
CALCRL	AC107419.1	0.452444748	2.78E-22	postive
FZD5	AC107419.1	0.735932644	8.78E-72	postive
GPC3	AC107419.1	0.510722139	6.98E-29	postive
KCNMB2	AC107419.1	0.471033566	2.98E-24	postive
SLC11A2	AC107419.1	0.45972918	4.85E-23	postive
SCARF1	AC010976.2	0.405785122	7.66E-18	postive
SELE	AC010976.2	0.431841582	3.09E-20	postive
FZD5	AC023024.1	0.554965688	8.15E-35	postive
GPC3	AC023024.1	0.418727764	5.26E-19	postive
SLC11A2	AC023024.1	0.484158962	1.02E-25	postive
SLC31A1	AL023284.4	-0.408825298	4.13E-18	negative
FZD5	LINC02615	0.445963935	1.26E-21	postive

ABCA1	BACH1-IT2	0.726166949	4.94E-69	postive
CYBB	BACH1-IT2	0.402902918	1.37E-17	postive
KIF1B	BACH1-IT2	0.410775581	2.77E-18	postive
TLR1	BACH1-IT2	0.420280574	3.78E-19	postive
ABCA1	RNF216-IT1	0.711802479	3.38E-65	postive
CYBB	RNF216-IT1	0.436472032	1.10E-20	postive
TLR1	RNF216-IT1	0.460367361	4.16E-23	postive
ABCA1	AC124069.1	0.476469261	7.50E-25	postive
FZD5	AC124069.1	0.656841437	1.82E-52	postive
KCNMB2	AC124069.1	0.492619084	1.07E-26	postive
SLC11A2	AC124069.1	0.423149982	2.05E-19	postive
APLNR	AL136084.2	0.691004156	4.80E-60	postive
C3AR1	AL136084.2	0.427874089	7.37E-20	postive
C5AR1	AL136084.2	0.406121483	7.16E-18	postive
CALCRL	AL136084.2	0.598460007	1.40E-41	postive
CCL24	AL136084.2	0.444616539	1.73E-21	postive
FPR1	AL136084.2	0.441061214	3.90E-21	postive
ITGA5	AL136084.2	0.582612615	5.36E-39	postive
PDE4B	AL136084.2	0.424075686	1.68E-19	postive
PIK3R5	AL136084.2	0.440473405	4.46E-21	postive

PTGIR	AL136084.2	0.420378584	3.71E-19	postive
SCARF1	AL136084.2	0.545964939	1.55E-33	postive
ABCA1	AL031775.2	0.437752997	8.25E-21	postive
GABBR1	AC011472.1	0.416275695	8.82E-19	postive
ABCA1	RORA-AS1	0.682776425	3.99E-58	postive
FZD5	RORA-AS1	0.487807966	3.89E-26	postive
SLC11A2	RORA-AS1	0.407117698	5.85E-18	postive
ABCA1	AL359878.1	0.504288218	4.32E-28	postive
FZD5	AL359878.1	0.692764664	1.83E-60	postive
GPC3	AL359878.1	0.409552599	3.56E-18	postive
KCNMB2	AL359878.1	0.489050258	2.80E-26	postive
SLC11A2	AL359878.1	0.466656568	8.88E-24	postive
ABCA1	AC019254.1	0.438301262	7.29E-21	postive
CYBB	AC019254.1	0.482987504	1.39E-25	postive
IL10RA	AC019254.1	0.408433572	4.47E-18	postive
NLRP3	AC019254.1	0.427609079	7.81E-20	postive
PIK3R5	AC019254.1	0.489837137	2.27E-26	postive
STAB1	AC019254.1	0.613507649	3.60E-44	postive
TACR1	AC019254.1	0.469574597	4.30E-24	postive
ABCA1	Z94721.1	0.578248579	2.61E-38	postive

FZD5	Z94721.1	0.453946605	1.94E-22	postive
TACR1	Z94721.1	0.402981972	1.35E-17	postive
ABCA1	AC018616.1	0.432255952	2.82E-20	postive
CALCRL	AC018616.1	0.483771238	1.13E-25	postive
FZD5	AC018616.1	0.747517906	3.30E-75	postive
GPC3	AC018616.1	0.625320255	2.65E-46	postive
KCNMB2	AC018616.1	0.457889017	7.57E-23	postive
SLC11A2	AC018616.1	0.464046646	1.69E-23	postive
ABCA1	AC024075.1	0.609835795	1.59E-43	postive
KIF1B	AC024075.1	0.496392833	3.85E-27	postive
STAB1	AC024075.1	0.443696593	2.13E-21	postive
TACR1	AC024075.1	0.475962062	8.54E-25	postive
ABCA1	AL512506.1	0.58166863	7.56E-39	postive
CALCRL	AL512506.1	0.420965163	3.27E-19	postive
FZD5	AL512506.1	0.678158313	4.48E-57	postive
GPC3	AL512506.1	0.406308481	6.89E-18	postive
KCNMB2	AL512506.1	0.475231268	1.03E-24	postive
SLC11A2	AL512506.1	0.44652061	1.11E-21	postive
ABCA1	AC105429.1	0.538826615	1.50E-32	postive
KCNMB2	TTLL1-AS1	0.527725337	4.64E-31	postive

ABCA1	JARID2-AS1	0.586363471	1.35E-39	postive
ABCA1	AP002907.1	0.526353328	7.03E-31	postive
KCNMB2	MUC20-OT1	0.466898578	8.37E-24	postive
ABCA1	AC093484.4	0.499206009	1.78E-27	postive
FZD5	AL162595.1	0.407296004	5.64E-18	postive
ABCA1	AC011773.1	0.456707744	1.01E-22	postive
ACVR2A	AL031282.2	0.440246852	4.69E-21	postive
RHOG	AL031282.2	-0.402496837	1.49E-17	negative
SLC11A2	AL031282.2	0.406326748	6.87E-18	postive
ABCA1	AC138956.2	0.571437983	2.93E-37	postive
FZD5	AC138956.2	0.478143079	4.88E-25	postive
SLC11A2	AC138956.2	0.404282309	1.04E-17	postive
ITGA5	AL139220.2	0.696852079	1.89E-61	postive
ITGA5	AC036108.3	0.576277833	5.28E-38	postive
FZD5	RPL37A-DT	0.497784349	2.63E-27	postive
ABCA1	AC093157.2	0.578894328	2.06E-38	postive
ABCA1	RBMS3-AS3	0.573506725	1.41E-37	postive
BEST1	RBMS3-AS3	0.429150003	5.58E-20	postive
CYBB	RBMS3-AS3	0.70567909	1.24E-63	postive
ADGRE1	RBMS3-AS3	0.432458252	2.69E-20	postive

IL10RA	RBMS3-AS3	0.467587082	7.05E-24	postive
LPAR1	RBMS3-AS3	0.453861462	1.98E-22	postive
NAMPT	RBMS3-AS3	0.418931183	5.04E-19	postive
PIK3R5	RBMS3-AS3	0.516515513	1.30E-29	postive
STAB1	RBMS3-AS3	0.55949171	1.79E-35	postive
TACR1	RBMS3-AS3	0.580286553	1.25E-38	postive
TLR1	RBMS3-AS3	0.708317217	2.65E-64	postive
ABCA1	EIPR1-IT1	0.495438081	5.00E-27	postive
KIF1B	EIPR1-IT1	0.421477733	2.93E-19	postive
ACVR1B	DHRS4-AS1	0.436188161	1.17E-20	postive
ACVR2A	DHRS4-AS1	0.446395136	1.14E-21	postive
ABCA1	SSBP3-AS1	0.553488714	1.33E-34	postive
KIF1B	SSBP3-AS1	0.411375781	2.44E-18	postive
ACVR2A	AC003070.1	0.434953761	1.55E-20	postive
ABCA1	AL139383.1	0.589732196	3.86E-40	postive
CYBB	AL139383.1	0.595967654	3.65E-41	postive
STAB1	AL139383.1	0.471258859	2.81E-24	postive
TACR1	AL139383.1	0.405923979	7.45E-18	postive
TLR1	AL139383.1	0.620559927	1.97E-45	postive
FZD5	FBXL19-AS1	0.40589878	7.49E-18	postive

ABCA1	AP005899.1	0.608877451	2.33E-43	postive
EIF2AK2	AP005899.1	0.435062863	1.51E-20	postive
ABCA1	AL139099.2	0.424567329	1.51E-19	postive
ABCA1	AC004943.1	0.607822495	3.56E-43	postive
ABCA1	AC006001.2	0.424204462	1.63E-19	postive
FZD5	AC006001.2	0.550162983	3.97E-34	postive
ABCA1	AC026202.2	0.563114985	5.24E-36	postive
ABCA1	SGMS1-AS1	0.647610027	1.38E-50	postive
CALCRL	SGMS1-AS1	0.456570159	1.04E-22	postive
FZD5	SGMS1-AS1	0.589310699	4.52E-40	postive
GPC3	SGMS1-AS1	0.47419363	1.34E-24	postive
SLC11A2	SGMS1-AS1	0.401537383	1.80E-17	postive
TACR1	SGMS1-AS1	0.427255403	8.44E-20	postive
TLR1	SGMS1-AS1	0.409211186	3.81E-18	postive
ABCA1	LINC02569	0.401209145	1.92E-17	postive
CALCRL	LINC02569	0.476396233	7.64E-25	postive
FZD5	LINC02569	0.620068324	2.42E-45	postive
GPC3	LINC02569	0.63995229	4.49E-49	postive
SLC11A2	LINC02569	0.497153845	3.13E-27	postive
ABCA1	AC079336.2	0.503762006	5.01E-28	postive

FZD5	AC253576.2	0.509724083	9.28E-29	postive
KCNMB2	AC253576.2	0.56403468	3.82E-36	postive
KCNJ2	KCNJ2-AS1	0.766296301	3.54E-81	postive
ABCA1	AL358334.3	0.416543407	8.33E-19	postive
ABCA1	AL117329.1	0.6111033	9.54E-44	postive
FZD5	AL117329.1	0.574293567	1.07E-37	postive
KCNMB2	AL117329.1	0.45032361	4.58E-22	postive
SLC11A2	AL117329.1	0.442397826	2.87E-21	postive
CD14	MYG1-AS1	0.430345298	4.29E-20	postive
CMKLR1	MYG1-AS1	0.406848424	6.18E-18	postive
CSF1	MYG1-AS1	0.454101025	1.87E-22	postive
EMP3	MYG1-AS1	0.484222702	1.00E-25	postive
TIMP1	MYG1-AS1	0.548977063	5.84E-34	postive
MSR1	AC092818.1	0.541390296	6.69E-33	postive
CD70	DUXAP8	0.41581574	9.71E-19	postive
ROS1	DUXAP8	0.514915086	2.08E-29	postive
ABCA1	AC022893.3	0.647897465	1.21E-50	postive
CALCRL	AC022893.3	0.466447592	9.36E-24	postive
FZD5	AC022893.3	0.600535173	6.27E-42	postive
KCNMB2	AC022893.3	0.437320134	9.10E-21	postive

SLC11A2	AC022893.3	0.410280616	3.06E-18	postive
ADORA2B	LINC00941	0.427584822	7.85E-20	postive
HRH1	LINC00941	0.42582317	1.15E-19	postive
OSMR	LINC00941	0.417043684	7.50E-19	postive
PLAUR	LINC00941	0.407293209	5.64E-18	postive
SPHK1	LINC00941	0.434080857	1.88E-20	postive
TPBG	LINC00941	0.401606232	1.78E-17	postive
ABCA1	AC234772.1	0.639457782	5.60E-49	postive
CYBB	AC234772.1	0.634339616	5.41E-48	postive
STAB1	AC234772.1	0.451263516	3.67E-22	postive
TLR1	AC234772.1	0.65674811	1.90E-52	postive
BTG2	LINC01353	0.405462773	8.18E-18	postive
CD40	LINC01353	0.437253623	9.24E-21	postive
CD48	LINC01353	0.444165493	1.92E-21	postive
EBI3	LINC01353	0.453971971	1.93E-22	postive
SLAMF1	LINC01353	0.516601388	1.27E-29	postive
CD82	C10orf55	0.471393752	2.72E-24	postive
IL4R	C10orf55	0.417401772	6.96E-19	postive
MMP14	C10orf55	0.460766785	3.77E-23	postive
ABCA1	AC009041.3	0.511395991	5.75E-29	postive

KIF1B	AC009041.3	0.43352319	2.13E-20	postive
ITGA5	AC027449.1	0.549451206	5.00E-34	postive
FZD5	PCAT1	0.531212608	1.60E-31	postive
KCNMB2	PCAT1	0.443391191	2.29E-21	postive
ABCA1	AP001625.2	0.429943902	4.69E-20	postive
FZD5	AP001625.2	0.49044459	1.93E-26	postive
SLC11A2	AP001625.2	0.446465219	1.13E-21	postive
GP1BA	EPS15-AS1	0.45805879	7.27E-23	postive
ADRM1	SNHG17	0.400025964	2.43E-17	postive
ABCA1	LINC01572	0.566097423	1.88E-36	postive
FZD5	LINC01572	0.506359751	2.41E-28	postive
KCNMB2	LINC01572	0.411203479	2.53E-18	postive
ITGA5	ADAMTS9-AS1	0.580256972	1.26E-38	postive
ABCA1	YEATS2-AS1	0.531411338	1.51E-31	postive
ABCA1	KDM4A-AS1	0.425628431	1.20E-19	postive
FZD5	KDM4A-AS1	0.542624284	4.52E-33	postive
GPC3	KDM4A-AS1	0.401411829	1.85E-17	postive
KCNMB2	AL391807.1	0.606298646	6.52E-43	postive
BDKRB1	IL6-AS1	0.525388816	9.40E-31	postive
BEST1	IL6-AS1	0.451135185	3.78E-22	postive

C3AR1	IL6-AS1	0.414114173	1.39E-18	postive
C5AR1	IL6-AS1	0.56753829	1.14E-36	postive
CCL2	IL6-AS1	0.515157905	1.94E-29	postive
CSF1	IL6-AS1	0.408429394	4.48E-18	postive
EMP3	IL6-AS1	0.439680391	5.34E-21	postive
FPR1	IL6-AS1	0.616109762	1.24E-44	postive
GPR183	IL6-AS1	0.501258306	1.01E-27	postive
IL10RA	IL6-AS1	0.426716435	9.49E-20	postive
IL18RAP	IL6-AS1	0.405721532	7.76E-18	postive
IL6	IL6-AS1	0.593120246	1.08E-40	postive
IL7R	IL6-AS1	0.411954145	2.17E-18	postive
LIF	IL6-AS1	0.460785984	3.75E-23	postive
LTA	IL6-AS1	0.40952699	3.57E-18	postive
NLRP3	IL6-AS1	0.464025579	1.70E-23	postive
PDE4B	IL6-AS1	0.493036002	9.59E-27	postive
PTGER2	IL6-AS1	0.417474075	6.85E-19	postive
SCN1B	IL6-AS1	0.401863531	1.69E-17	postive
TIMP1	IL6-AS1	0.434965972	1.54E-20	postive
TNFAIP6	IL6-AS1	0.464793746	1.41E-23	postive
TNFRSF1B	IL6-AS1	0.433529127	2.12E-20	postive

ABCA1	AC012358.2	0.548627949	6.54E-34	postive
CYBB	AC012358.2	0.449567769	5.46E-22	postive
STAB1	AC012358.2	0.413302273	1.64E-18	postive
TLR1	AC012358.2	0.518795316	6.69E-30	postive
MET	LINC01393	0.441145511	3.83E-21	postive
IFITM1	LINC02574	0.453943907	1.95E-22	postive
IRF7	LINC02574	0.516603506	1.27E-29	postive
LAMP3	LINC02574	0.4088918	4.07E-18	postive
P2RX4	ADCY6-DT	0.404649993	9.64E-18	postive
CD48	LINC01781	0.563433814	4.70E-36	postive
GP1BA	LINC01781	0.620191082	2.30E-45	postive
GPR183	LINC01781	0.516391432	1.35E-29	postive
IL10RA	LINC01781	0.420255631	3.80E-19	postive
KCNA3	LINC01781	0.536083366	3.55E-32	postive
LCK	LINC01781	0.521201818	3.28E-30	postive
LTA	LINC01781	0.658162811	9.65E-53	postive
PDE4B	LINC01781	0.436801005	1.02E-20	postive
EIF2AK2	SNHG9	-0.419126344	4.83E-19	negative
ABCA1	AP003900.1	0.621619008	1.26E-45	postive
CYBB	AP003900.1	0.714243918	7.82E-66	postive

IL10RA	AP003900.1	0.429649091	5.00E-20	postive
PIK3R5	AP003900.1	0.478159686	4.86E-25	postive
STAB1	AP003900.1	0.58595123	1.57E-39	postive
TACR1	AP003900.1	0.46083594	3.71E-23	postive
TLR1	AP003900.1	0.705505578	1.37E-63	postive
ABCA1	AC004466.3	0.507877919	1.57E-28	postive
KIF1B	SNHG16	0.448494897	7.02E-22	postive
BST2	LINC02195	0.405230128	8.57E-18	postive
CXCL10	LINC02195	0.557354112	3.67E-35	postive
CXCL11	LINC02195	0.559995217	1.51E-35	postive
CXCL9	LINC02195	0.481435713	2.08E-25	postive
CXCR6	LINC02195	0.66390358	5.95E-54	postive
IFITM1	LINC02195	0.408442821	4.46E-18	postive
IL15	LINC02195	0.48501598	8.15E-26	postive
IL15RA	LINC02195	0.591448977	2.02E-40	postive
IL18RAP	LINC02195	0.727202392	2.56E-69	postive
IL2RB	LINC02195	0.429945017	4.69E-20	postive
IRF1	LINC02195	0.578147548	2.70E-38	postive
LCK	LINC02195	0.430787781	3.89E-20	postive
LCP2	LINC02195	0.463371958	2.00E-23	postive

NMI	LINC02195	0.482644012	1.52E-25	postive
PTAFR	LINC02195	0.478660846	4.27E-25	postive
RGS1	LINC02195	0.489532474	2.46E-26	postive
TAPBP	LINC02195	0.534961393	5.04E-32	postive
FZD5	AC010201.2	0.467770567	6.74E-24	postive
ABCA1	AC091729.2	0.550015505	4.16E-34	postive
FZD5	AC091729.2	0.405811667	7.62E-18	postive
FZD5	AC107027.3	0.464399129	1.55E-23	postive
ABCA1	AC058791.1	0.746319429	7.61E-75	postive
CYBB	AC058791.1	0.462320522	2.58E-23	postive
FZD5	AC058791.1	0.407710252	5.18E-18	postive
KIF1B	AC058791.1	0.40860934	4.31E-18	postive
TACR1	AC058791.1	0.411725006	2.27E-18	postive
TLR1	AC058791.1	0.484686252	8.89E-26	postive
ABCA1	FAM13A-AS1	0.558139124	2.82E-35	postive
FZD5	FAM13A-AS1	0.479135077	3.78E-25	postive
FZD5	MORC2-AS1	0.6573095	1.45E-52	postive
KCNMB2	MORC2-AS1	0.45421619	1.82E-22	postive
SLC11A2	MORC2-AS1	0.412341904	2.00E-18	postive
SGMS2	AL671710.1	-0.431861602	3.07E-20	negative

CCL24	AC090125.1	0.733573692	4.16E-71	postive
CCL7	AC083967.1	0.753899813	3.55E-77	postive
ABCA1	KCNIP2-AS1	0.525063314	1.04E-30	postive
FZD5	KCNIP2-AS1	0.443070584	2.46E-21	postive
KCNMB2	KCNIP2-AS1	0.500427401	1.27E-27	postive
TACR1	KCNIP2-AS1	0.427570057	7.88E-20	postive
ABCA1	AL035530.2	0.477838568	5.28E-25	postive
TLR1	AL035530.2	0.4273124	8.33E-20	postive
APLNR	LINC01936	0.484011484	1.06E-25	postive
ITGA5	LINC01936	0.435071457	1.51E-20	postive
PTGIR	LINC01936	0.520014874	4.67E-30	postive
SCN1B	LINC01936	0.413285554	1.65E-18	postive
TIMP1	LINC01936	0.401254682	1.91E-17	postive
FZD5	LINC02163	0.495996087	4.29E-27	postive
KCNMB2	LINC02163	0.429453224	5.22E-20	postive
C3AR1	LINC01615	0.424345078	1.58E-19	postive
CCL24	LINC01615	0.591935862	1.69E-40	postive
NOD2	LINC01615	0.427819948	7.46E-20	postive
PLAUR	LINC01615	0.427106375	8.72E-20	postive
SPHK1	LINC01615	0.430014376	4.62E-20	postive

FZD5	AC007128.1	0.559651088	1.70E-35	postive
SLC11A2	AC007128.1	0.477470702	5.80E-25	postive
ABCA1	TBL1XR1-AS1	0.640485195	3.53E-49	postive
FZD5	TBL1XR1-AS1	0.522569647	2.19E-30	postive
KCNMB2	TBL1XR1-AS1	0.440944665	4.01E-21	postive
ABCA1	WAKMAR2	0.440738566	4.20E-21	postive
CYBB	WAKMAR2	0.402294745	1.55E-17	postive
IL10RA	WAKMAR2	0.406849242	6.18E-18	postive
KCNA3	WAKMAR2	0.418236272	5.83E-19	postive
LCP2	WAKMAR2	0.423497432	1.90E-19	postive
P2RX7	WAKMAR2	0.417176055	7.30E-19	postive
PIK3R5	WAKMAR2	0.404344762	1.03E-17	postive
TLR1	WAKMAR2	0.45654763	1.04E-22	postive
ABCA1	AC023794.1	0.513451714	3.18E-29	postive
FZD5	AC023794.1	0.622219321	9.83E-46	postive
KCNMB2	AC023794.1	0.509492435	9.92E-29	postive
SLC11A2	AC023794.1	0.422744273	2.24E-19	postive
FZD5	LNX1-AS2	0.581110704	9.26E-39	postive
GPC3	LNX1-AS2	0.468429046	5.72E-24	postive
SLC11A2	LNX1-AS2	0.457387928	8.54E-23	postive

ABCA1	AC023825.2	0.700657916	2.21E-62	postive
BEST1	AC023825.2	0.438877676	6.40E-21	postive
CYBB	AC023825.2	0.706007367	1.02E-63	postive
IL10RA	AC023825.2	0.502259502	7.63E-28	postive
LCP2	AC023825.2	0.408955867	4.02E-18	postive
MSR1	AC023825.2	0.415158297	1.11E-18	postive
P2RX7	AC023825.2	0.466103409	1.02E-23	postive
PIK3R5	AC023825.2	0.524266714	1.32E-30	postive
STAB1	AC023825.2	0.52464868	1.17E-30	postive
TACR1	AC023825.2	0.455496152	1.34E-22	postive
TLR1	AC023825.2	0.705557189	1.33E-63	postive
ABCA1	AC005954.1	0.436050074	1.21E-20	postive
ABCA1	AL359921.1	0.460838405	3.71E-23	postive
ABCA1	AP006545.1	0.42248677	2.36E-19	postive
ABCA1	PSPC1-AS2	0.447617352	8.62E-22	postive
FZD5	PSPC1-AS2	0.460834431	3.71E-23	postive
SLC11A2	PSPC1-AS2	0.403370856	1.25E-17	postive
FZD5	AC009961.1	0.41086489	2.72E-18	postive
ABCA1	AC008781.2	0.444580915	1.74E-21	postive
FZD5	AC008781.2	0.662663209	1.09E-53	postive

KCNMB2	AC008781.2	0.48578296	6.66E-26	postive
ABCA1	AL031666.2	0.574653078	9.42E-38	postive
FZD5	AL031666.2	0.599039386	1.12E-41	postive
KCNMB2	AL031666.2	0.475885757	8.71E-25	postive
ABCA1	AC069277.1	0.477337055	6.00E-25	postive
CYBB	AC069277.1	0.535993122	3.65E-32	postive
TLR1	AC069277.1	0.557385185	3.64E-35	postive
ACVR2A	AC008764.2	0.419826223	4.17E-19	postive
ABCA1	FENDRR	0.457840969	7.66E-23	postive
BEST1	FENDRR	0.448381961	7.21E-22	postive
CYBB	FENDRR	0.599902627	8.01E-42	postive
IL10RA	FENDRR	0.48270245	1.50E-25	postive
ITGA5	FENDRR	0.43140078	3.40E-20	postive
LPAR1	FENDRR	0.425108038	1.34E-19	postive
PIK3R5	FENDRR	0.529357053	2.83E-31	postive
STAB1	FENDRR	0.599280267	1.02E-41	postive
TACR1	FENDRR	0.681421485	8.16E-58	postive
TLR1	FENDRR	0.55828638	2.69E-35	postive
MEP1A	LINC01843	0.551765673	2.35E-34	postive
FZD5	AL158834.2	0.509315221	1.04E-28	postive

ABCA1	AL731577.2	0.479403146	3.53E-25	postive
KIF1B	AL731577.2	0.617530517	6.93E-45	postive
ABCA1	AL136221.1	0.400702326	2.13E-17	postive
FZD5	AL136221.1	0.459078988	5.68E-23	postive
SLC11A2	AL136221.1	0.408253658	4.64E-18	postive
APLNR	LINC02104	0.405805688	7.63E-18	postive
AXL	LINC02104	0.536853402	2.79E-32	postive
C3AR1	LINC02104	0.480778791	2.47E-25	postive
CMKLR1	LINC02104	0.444289645	1.86E-21	postive
CSF1	LINC02104	0.458462466	6.59E-23	postive
EMP3	LINC02104	0.458378161	6.73E-23	postive
FPR1	LINC02104	0.491651756	1.39E-26	postive
MMP14	LINC02104	0.440067399	4.89E-21	postive
PTGIR	LINC02104	0.401727092	1.73E-17	postive
SCN1B	LINC02104	0.436304687	1.14E-20	postive
SPHK1	LINC02104	0.417251433	7.18E-19	postive
TIMP1	LINC02104	0.489156214	2.72E-26	postive
TNFAIP6	LINC02104	0.413693227	1.51E-18	postive
ABCA1	COL4A2-AS1	0.719586394	3.02E-67	postive
CYBB	COL4A2-AS1	0.52767785	4.71E-31	postive

IL10RA	COL4A2-AS1	0.407569491	5.33E-18	postive
NLRP3	COL4A2-AS1	0.414181196	1.37E-18	postive
PIK3R5	COL4A2-AS1	0.52018596	4.44E-30	postive
SCARF1	COL4A2-AS1	0.495864626	4.45E-27	postive
STAB1	COL4A2-AS1	0.707946623	3.30E-64	postive
TACR1	COL4A2-AS1	0.684021055	2.07E-58	postive
TLR1	COL4A2-AS1	0.449327884	5.78E-22	postive
IL1R1	SLC2A9-AS1	0.408988012	3.99E-18	postive
ABCA1	AC105137.2	0.4379379	7.92E-21	postive
GABBR1	LINC01992	0.416025216	9.29E-19	postive
IRF7	NRIR	0.52381047	1.51E-30	postive
LAMP3	NRIR	0.472446233	2.09E-24	postive
RTP4	NRIR	0.413830732	1.47E-18	postive
FZD5	AC004477.2	0.477603166	5.61E-25	postive
KCNMB2	AC004477.2	0.494918636	5.76E-27	postive
CD82	CYTOR	0.406735318	6.32E-18	postive
EMP3	CYTOR	0.589196979	4.71E-40	postive
MMP14	CYTOR	0.409902378	3.31E-18	postive
PLAUR	CYTOR	0.485426342	7.32E-26	postive
RHOG	CYTOR	0.419811341	4.18E-19	postive

SCN1B	CYTOR	0.568237329	8.97E-37	postive
SPHK1	CYTOR	0.465360681	1.22E-23	postive
TNFRSF1B	CYTOR	0.443680618	2.14E-21	postive
OSMR	GATA3-AS1	-0.410809029	2.75E-18	negative
SGMS2	GATA3-AS1	-0.40757896	5.32E-18	negative
IL18	LINC01836	0.447014941	9.91E-22	postive
ABCA1	AC020978.2	0.771143162	8.26E-83	postive
CYBB	AC020978.2	0.510706508	7.01E-29	postive
PIK3R5	AC020978.2	0.400047689	2.42E-17	postive
STAB1	AC020978.2	0.603683406	1.83E-42	postive
TACR1	AC020978.2	0.604396606	1.38E-42	postive
TLR1	AC020978.2	0.476486866	7.47E-25	postive
KCNMB2	MIS18A-AS1	0.491370231	1.50E-26	postive
ABCA1	SRI-AS1	0.525692803	8.58E-31	postive
CALCRL	SRI-AS1	0.439894836	5.08E-21	postive
FZD5	SRI-AS1	0.708368985	2.57E-64	postive
GPC3	SRI-AS1	0.475658796	9.23E-25	postive
KCNMB2	SRI-AS1	0.474057204	1.39E-24	postive
SLC11A2	SRI-AS1	0.474657529	1.19E-24	postive
KCNMB2	AL359881.1	0.436033553	1.21E-20	postive

DCBLD2	HLA-F-AS1	0.536268038	3.35E-32	postive
KIF1B	COX10-AS1	0.455651141	1.29E-22	postive
BEST1	LINC00861	0.436256765	1.16E-20	postive
C3AR1	LINC00861	0.409415262	3.66E-18	postive
CD48	LINC00861	0.661451027	1.97E-53	postive
CXCL9	LINC00861	0.422399581	2.41E-19	postive
CXCR6	LINC00861	0.486028783	6.24E-26	postive
GP1BA	LINC00861	0.562591386	6.26E-36	postive
GPR132	LINC00861	0.559866587	1.58E-35	postive
GPR183	LINC00861	0.615909696	1.35E-44	postive
IL10RA	LINC00861	0.672148914	9.76E-56	postive
IL18RAP	LINC00861	0.412834839	1.81E-18	postive
IL2RB	LINC00861	0.469267752	4.64E-24	postive
IL7R	LINC00861	0.518740102	6.80E-30	postive
KCNA3	LINC00861	0.755771133	9.15E-78	postive
LCK	LINC00861	0.694896183	5.63E-61	postive
LCP2	LINC00861	0.580180148	1.30E-38	postive
LTA	LINC00861	0.762104463	8.50E-80	postive
P2RX7	LINC00861	0.472243507	2.20E-24	postive
PDE4B	LINC00861	0.548968798	5.86E-34	postive

PIK3R5	LINC00861	0.551593718	2.48E-34	postive
RASGRP1	LINC00861	0.451396792	3.55E-22	postive
RGS1	LINC00861	0.504405615	4.18E-28	postive
SEMA4D	LINC00861	0.401945613	1.66E-17	postive
TNFRSF1B	LINC00861	0.562747846	5.94E-36	postive
TNFRSF9	LINC00861	0.513866324	2.82E-29	postive
CCRL2	CCR5AS	0.706705213	6.81E-64	postive
SLC28A2	AC093001.1	0.646525988	2.27E-50	postive
ABCA1	AC092279.1	0.509865334	8.91E-29	postive
FZD5	AC092279.1	0.70070668	2.15E-62	postive
GPC3	AC092279.1	0.460220992	4.31E-23	postive
KCNMB2	AC092279.1	0.491742064	1.36E-26	postive
SLC11A2	AC092279.1	0.471300103	2.79E-24	postive
ABCA1	AC005104.1	0.546258562	1.41E-33	postive
FZD5	AC005104.1	0.408503974	4.41E-18	postive
ABCA1	CDC42-IT1	0.632891925	1.02E-47	postive
FZD5	CDC42-IT1	0.569228106	6.35E-37	postive
KCNMB2	CDC42-IT1	0.461806124	2.93E-23	postive
SLC11A2	CDC42-IT1	0.412517079	1.93E-18	postive
CD48	LINC02202	0.618342865	4.95E-45	postive

EBI3	LINC02202	0.586792214	1.15E-39	postive
GPR183	LINC02202	0.416243391	8.88E-19	postive
ITGA5	LINC02202	0.404443368	1.01E-17	postive
LCK	LINC02202	0.464035992	1.70E-23	postive
LTA	LINC02202	0.408258011	4.63E-18	postive
PTGIR	LINC02202	0.581533278	7.94E-39	postive
SLAMF1	LINC02202	0.68723226	3.71E-59	postive
ABCA1	AC006160.1	0.639397657	5.75E-49	postive
CYBB	AC006160.1	0.487919795	3.78E-26	postive
TLR1	AC006160.1	0.522714781	2.09E-30	postive
PTPRE	AL158166.1	0.800721674	1.00E-93	postive
SELL	SOCS2-AS1	0.728781896	9.31E-70	postive
ABCA1	AL049780.1	0.411067574	2.60E-18	postive
ABCA1	AC011466.1	0.563459957	4.66E-36	postive
FZD5	AC011466.1	0.478399251	4.57E-25	postive
ABCA1	AC025031.2	0.802815382	1.44E-94	postive
CYBB	AC025031.2	0.418968698	5.00E-19	postive
KIF1B	AC025031.2	0.432350682	2.76E-20	postive
STAB1	AC025031.2	0.410565324	2.89E-18	postive
TACR1	AC025031.2	0.431327	3.46E-20	postive

TLR1	AC025031.2	0.405637543	7.90E-18	postive
KCNMB2	AC093249.6	0.401693246	1.75E-17	postive
C3AR1	AC243960.3	0.40168283	1.75E-17	postive
C5AR1	AC243960.3	0.410814969	2.74E-18	postive
CD48	AC243960.3	0.683694381	2.46E-58	postive
CYBB	AC243960.3	0.459097853	5.65E-23	postive
EBI3	AC243960.3	0.572762684	1.84E-37	postive
GP1BA	AC243960.3	0.514366029	2.44E-29	postive
GPR132	AC243960.3	0.413856199	1.46E-18	postive
GPR183	AC243960.3	0.564154328	3.67E-36	postive
IL10RA	AC243960.3	0.583429697	3.98E-39	postive
KCNA3	AC243960.3	0.497766333	2.64E-27	postive
LCK	AC243960.3	0.661413963	2.01E-53	postive
LCP2	AC243960.3	0.549923721	4.29E-34	postive
LTA	AC243960.3	0.635594781	3.11E-48	postive
PIK3R5	AC243960.3	0.552603078	1.78E-34	postive
PTGIR	AC243960.3	0.464416662	1.54E-23	postive
RASGRP1	AC243960.3	0.421987896	2.63E-19	postive
SLAMF1	AC243960.3	0.535753749	3.94E-32	postive
TNFRSF1B	AC243960.3	0.487952712	3.75E-26	postive

TNFRSF9	AC243960.3	0.425792648	1.16E-19	postive
ABCA1	AC006064.2	0.59632799	3.18E-41	postive
FZD5	AC006064.2	0.502983793	6.23E-28	postive
SLC11A2	AC006064.2	0.402206674	1.58E-17	postive
FZD5	AC025178.1	0.491752841	1.36E-26	postive
ABCA1	XXYLT1-AS2	0.45404102	1.90E-22	postive
GP1BA	XXYLT1-AS2	0.465569933	1.16E-23	postive
IL10RA	XXYLT1-AS2	0.420250294	3.81E-19	postive
KCNA3	XXYLT1-AS2	0.450365103	4.53E-22	postive
LTA	XXYLT1-AS2	0.43905317	6.15E-21	postive
CCL20	AL358394.1	0.440863271	4.08E-21	postive
EREG	AL358394.1	0.574834974	8.83E-38	postive
HIF1A	AL358394.1	0.458367107	6.74E-23	postive
ABCA1	AL031710.2	0.539622061	1.17E-32	postive
FZD5	AL031710.2	0.471732918	2.50E-24	postive
KCNMB2	AL031710.2	0.42235532	2.43E-19	postive
FZD5	C1orf220	0.403762553	1.15E-17	postive
ACVR2A	AC008124.1	0.517711802	9.19E-30	postive
PLAUR	AC008124.1	-0.439584567	5.46E-21	negative
RHOG	AC008124.1	-0.404143643	1.07E-17	negative

CALCRL	LINC01414	0.44810183	7.70E-22	postive
FZD5	LINC01414	0.708760869	2.04E-64	postive
GPC3	LINC01414	0.600319138	6.82E-42	postive
KCNMB2	LINC01414	0.42010238	3.93E-19	postive
SLC11A2	LINC01414	0.444069687	1.96E-21	postive
FZD5	AC006206.2	0.403495755	1.22E-17	postive
SCN1B	AC010457.1	0.402013128	1.64E-17	postive
KIF1B	AC120114.1	0.480728474	2.50E-25	postive
ABCA1	AC011815.1	0.471305762	2.78E-24	postive
ABCA1	AC005740.4	0.500568561	1.22E-27	postive
CYBB	AC005740.4	0.459535846	5.09E-23	postive
TLR1	AC005740.4	0.465445465	1.20E-23	postive
CD48	LINC01215	0.664937374	3.58E-54	postive
EBI3	LINC01215	0.448553208	6.93E-22	postive
GP1BA	LINC01215	0.596588877	2.87E-41	postive
GPR183	LINC01215	0.474263505	1.32E-24	postive
KCNA3	LINC01215	0.42953746	5.13E-20	postive
LCK	LINC01215	0.646252643	2.58E-50	postive
LTA	LINC01215	0.690475266	6.41E-60	postive
SLAMF1	LINC01215	0.432713039	2.55E-20	postive

KCNMB2	AC092127.2	0.833130967	5.02E-108	postive
ABCA1	LINC02156	0.452818633	2.54E-22	postive
FZD5	LINC02156	0.519068236	6.17E-30	postive
KCNMB2	LINC02156	0.410920284	2.68E-18	postive
ABCA1	AC009088.1	0.472292753	2.17E-24	postive
KCNMB2	AC009088.1	0.448435158	7.12E-22	postive
ABCA1	AC010973.1	0.67156635	1.31E-55	postive
KIF1B	AC010973.1	0.44700497	9.94E-22	postive
STAB1	AC010973.1	0.552051818	2.14E-34	postive
TACR1	AC010973.1	0.517505474	9.77E-30	postive
GPC3	AC015908.3	0.458557238	6.44E-23	postive
ABCA1	GAS8-AS1	0.491015821	1.65E-26	postive
ABCA1	AC016405.1	0.606905931	5.12E-43	postive
FZD5	AC016405.1	0.572547362	1.98E-37	postive
KCNMB2	AC016405.1	0.473007359	1.81E-24	postive
SLC11A2	AC016405.1	0.401875327	1.68E-17	postive
PROK2	MEG9	0.525839129	8.21E-31	postive
VIP	MEG9	0.656495541	2.14E-52	postive
ADM	AC080023.1	0.430105913	4.52E-20	postive
NAMPT	AC080023.1	0.474011164	1.40E-24	postive

ABCA1	AC025280.1	0.599806859	8.31E-42	postive
CYBB	AC025280.1	0.413941408	1.44E-18	postive
TLR1	AC025280.1	0.43634849	1.13E-20	postive
ACVR2A	AP000866.1	0.425867436	1.14E-19	postive
FZD5	AP000866.1	0.422450849	2.38E-19	postive
SLC11A2	AP000866.1	0.419060362	4.90E-19	postive
BEST1	AL365361.1	0.430291784	4.34E-20	postive
C3AR1	AL365361.1	0.450059693	4.87E-22	postive
C5AR1	AL365361.1	0.463358051	2.00E-23	postive
CD48	AL365361.1	0.686388777	5.84E-59	postive
CXCL9	AL365361.1	0.516980293	1.14E-29	postive
CXCR6	AL365361.1	0.567267156	1.26E-36	postive
CYBB	AL365361.1	0.442374357	2.89E-21	postive
GP1BA	AL365361.1	0.590114789	3.34E-40	postive
GPR132	AL365361.1	0.604242748	1.47E-42	postive
GPR183	AL365361.1	0.705411191	1.45E-63	postive
IL10RA	AL365361.1	0.730266892	3.58E-70	postive
IL18RAP	AL365361.1	0.483969344	1.07E-25	postive
IL2RB	AL365361.1	0.53177738	1.35E-31	postive
IL7R	AL365361.1	0.565295106	2.48E-36	postive

KCNA3	AL365361.1	0.938596267	1.63E-192	postive
LCK	AL365361.1	0.702978604	5.88E-63	postive
LCP2	AL365361.1	0.654672473	5.09E-52	postive
LTA	AL365361.1	0.743421563	5.63E-74	postive
P2RX7	AL365361.1	0.53800887	1.95E-32	postive
PDE4B	AL365361.1	0.656723506	1.92E-52	postive
PIK3R5	AL365361.1	0.605631387	8.49E-43	postive
PTAFR	AL365361.1	0.444666675	1.71E-21	postive
RASGRP1	AL365361.1	0.482144438	1.73E-25	postive
RGS1	AL365361.1	0.600099879	7.42E-42	postive
SEMA4D	AL365361.1	0.44018235	4.76E-21	postive
TLR1	AL365361.1	0.410360702	3.01E-18	postive
TNFRSF1B	AL365361.1	0.615903754	1.35E-44	postive
TNFRSF9	AL365361.1	0.614890815	2.05E-44	postive
ABCA1	AL590729.1	0.641924511	1.85E-49	postive
FZD5	AL590729.1	0.558442488	2.55E-35	postive
KCNMB2	AL590729.1	0.428442555	6.51E-20	postive
SLC11A2	AL590729.1	0.442270444	2.96E-21	postive
ABCA1	AL162734.1	0.497213568	3.08E-27	postive
SCARF1	AC026369.2	0.429896181	4.74E-20	postive

ABCA1	AP001001.1	0.569366304	6.06E-37	postive
FZD5	AP001001.1	0.411444221	2.41E-18	postive
ABCA1	AL731566.2	0.569809541	5.19E-37	postive
FZD5	AL731566.2	0.512400405	4.31E-29	postive
SLC11A2	AL731566.2	0.426635749	9.66E-20	postive
ABCA1	AP001972.1	0.420606532	3.53E-19	postive
STAB1	AP001972.1	0.540435484	9.06E-33	postive
TACR1	AP001972.1	0.495647906	4.72E-27	postive
CD48	AL132642.1	0.504040697	4.64E-28	postive
CYBB	AL132642.1	0.401771262	1.72E-17	postive
EBI3	AL132642.1	0.555315994	7.25E-35	postive
SLAMF1	AL132642.1	0.637851204	1.15E-48	postive
TLR1	AL132642.1	0.407898331	4.99E-18	postive
KCNMB2	NFYC-AS1	0.401437879	1.84E-17	postive
FZD5	AL158835.2	0.403604648	1.19E-17	postive
KCNMB2	AL158835.2	0.418058298	6.06E-19	postive
NMUR1	LINC01397	0.634043421	6.16E-48	postive
ABCA1	AC069549.1	0.694846722	5.79E-61	postive
CYBB	AC069549.1	0.632276528	1.33E-47	postive
PIK3R5	AC069549.1	0.433017847	2.38E-20	postive

STAB1	AC069549.1	0.497852666	2.58E-27	postive
TACR1	AC069549.1	0.453017836	2.42E-22	postive
TLR1	AC069549.1	0.63931617	5.97E-49	postive
ABCA1	AC010422.4	0.625780035	2.18E-46	postive
FZD5	AC010422.4	0.467907431	6.51E-24	postive
KCNMB2	AC010422.4	0.400441059	2.24E-17	postive
ABCA1	AC104170.1	0.75134485	2.21E-76	postive
CYBB	AC104170.1	0.464918613	1.37E-23	postive
STAB1	AC104170.1	0.409963385	3.27E-18	postive
TACR1	AC104170.1	0.454662132	1.64E-22	postive
TLR1	AC104170.1	0.452323867	2.86E-22	postive
MEP1A	PTPRD-AS1	0.54226514	5.07E-33	postive
ABCA1	AC079313.1	0.4636476	1.87E-23	postive
ITGA5	AC079313.1	0.492071693	1.24E-26	postive
ABCA1	MED4-AS1	0.513412607	3.22E-29	postive
FZD5	MED4-AS1	0.566400544	1.70E-36	postive
KCNMB2	MED4-AS1	0.417545557	6.75E-19	postive
SLC11A2	MED4-AS1	0.408267576	4.63E-18	postive
FZD5	AL031716.1	0.438221836	7.43E-21	postive
FZD5	LINC02159	0.443964418	2.01E-21	postive

GPC3	LINC02159	0.532383929	1.12E-31	postive
ABCA1	TRAF3IP2-AS1	0.55565178	6.49E-35	postive
FZD5	TRAF3IP2-AS1	0.400009644	2.44E-17	postive
KCNMB2	TRAF3IP2-AS1	0.553726417	1.23E-34	postive
KIF1B	TRAF3IP2-AS1	0.450517334	4.37E-22	postive
FFAR2	AC002511.2	0.548789304	6.21E-34	postive
ABCA1	AL353804.1	0.710724865	6.41E-65	postive
CYBB	AL353804.1	0.415410613	1.06E-18	postive
FZD5	AL353804.1	0.430315892	4.32E-20	postive
KIF1B	AL353804.1	0.430667056	4.00E-20	postive
TLR1	AL353804.1	0.456542	1.05E-22	postive
FZD5	AC068196.1	0.412788933	1.82E-18	postive
ABCA1	AP001469.1	0.567542202	1.14E-36	postive
KIF1B	LINC00654	0.410190006	3.12E-18	postive
VIP	LINC00654	0.529425231	2.77E-31	postive
ABCA1	FO680682.1	0.720579827	1.64E-67	postive
CYBB	FO680682.1	0.41419307	1.36E-18	postive
FZD5	FO680682.1	0.468067	6.26E-24	postive
KIF1B	FO680682.1	0.400472752	2.23E-17	postive
TLR1	FO680682.1	0.447342895	9.19E-22	postive

ABCA1	AC134407.1	0.693865956	9.97E-61	postive
FZD5	AC134407.1	0.521774798	2.77E-30	postive
KCNMB2	AC134407.1	0.418658411	5.34E-19	postive
TACR1	AC134407.1	0.440121566	4.83E-21	postive
AXL	NKILA	0.494371311	6.68E-27	postive
MET	NKILA	0.465062587	1.32E-23	postive
MMP14	NKILA	0.474211383	1.33E-24	postive
PLAUR	NKILA	0.458983888	5.81E-23	postive
SERPINE1	NKILA	0.401553337	1.79E-17	postive
SPHK1	NKILA	0.427115961	8.70E-20	postive
ABCA1	AL021368.2	0.467233439	7.70E-24	postive
FZD5	AL021368.2	0.469663151	4.20E-24	postive
KCNMB2	AL021368.2	0.403872883	1.13E-17	postive
SLC11A2	AL021368.2	0.402998878	1.34E-17	postive
ITGA5	AC002398.2	0.604755398	1.20E-42	postive
CCL24	AC112721.1	0.821089554	2.24E-102	postive
CMKLR1	AC112721.1	0.417694349	6.54E-19	postive
AXL	LINC01119	0.411409351	2.43E-18	postive
BDKRB1	LINC01119	0.461572928	3.10E-23	postive
BEST1	LINC01119	0.455029181	1.50E-22	postive

C5AR1	LINC01119	0.510592691	7.24E-29	postive
CCL2	LINC01119	0.439941137	5.03E-21	postive
FPR1	LINC01119	0.506872676	2.09E-28	postive
IL6	LINC01119	0.402508335	1.48E-17	postive
ITGA5	LINC01119	0.422755265	2.23E-19	postive
LIF	LINC01119	0.404655733	9.63E-18	postive
NLRP3	LINC01119	0.444070897	1.96E-21	postive
SCN1B	LINC01119	0.426671304	9.58E-20	postive
TIMP1	LINC01119	0.458625574	6.34E-23	postive
TNFAIP6	LINC01119	0.481628275	1.98E-25	postive
ABCA1	LSAMP-AS1	0.532686305	1.02E-31	postive
CYBB	LSAMP-AS1	0.607383867	4.24E-43	postive
STAB1	LSAMP-AS1	0.449164232	6.01E-22	postive
TLR1	LSAMP-AS1	0.668594745	5.84E-55	postive
CXCL9	LYPLAL1-DT	0.524814488	1.12E-30	postive
ITGB3	LYPLAL1-DT	0.500331124	1.30E-27	postive
MSR1	LYPLAL1-DT	0.402876086	1.38E-17	postive
P2RX7	LYPLAL1-DT	0.429513506	5.15E-20	postive
ROS1	LYPLAL1-DT	0.539629223	1.17E-32	postive
TNFRSF9	LYPLAL1-DT	0.567782095	1.05E-36	postive

KCNMB2	AL121929.2	0.597060452	2.40E-41	postive
ABCA1	AL139041.1	0.401714456	1.74E-17	postive
FZD5	AL139041.1	0.593462766	9.46E-41	postive
KCNMB2	AL139041.1	0.413779611	1.48E-18	postive
ABCA1	FMR1-IT1	0.483707607	1.15E-25	postive
SLC11A2	FMR1-IT1	0.411582265	2.34E-18	postive
KCNMB2	AC110285.2	0.439671338	5.35E-21	postive
ABCA1	LINC01775	0.441238618	3.75E-21	postive
CYBB	LINC01775	0.42153337	2.90E-19	postive
KIF1B	LINC01775	0.486010484	6.27E-26	postive
PIK3R5	LINC01775	0.470326049	3.56E-24	postive
STAB1	LINC01775	0.546772048	1.19E-33	postive
TACR1	LINC01775	0.409376654	3.69E-18	postive
ABCA1	AC253536.6	0.423664814	1.83E-19	postive
ABCA1	RNASEH2B-AS1	0.502700212	6.74E-28	postive
CALCRL	RNASEH2B-AS1	0.418080451	6.03E-19	postive
FZD5	RNASEH2B-AS1	0.690295811	7.07E-60	postive
GPC3	RNASEH2B-AS1	0.430594289	4.06E-20	postive
KCNMB2	RNASEH2B-AS1	0.487373803	4.37E-26	postive
SLC11A2	RNASEH2B-AS1	0.436440053	1.11E-20	postive

ABCA1	AC020663.3	0.40322075	1.29E-17	postive
ABCA1	AC009318.3	0.434046572	1.89E-20	postive
CALCRL	AC009318.3	0.424291313	1.60E-19	postive
FZD5	AC009318.3	0.689575172	1.05E-59	postive
GPC3	AC009318.3	0.462704568	2.35E-23	postive
KCNMB2	AC009318.3	0.471779893	2.47E-24	postive
SLC11A2	AC009318.3	0.4394622	5.61E-21	postive
SGMS2	AC009065.8	-0.414423433	1.30E-18	negative
ABCA1	LINC01194	0.431544731	3.30E-20	postive
FZD5	LINC01194	0.475749008	9.02E-25	postive
FZD5	AC009121.2	0.427783758	7.52E-20	postive
CXCR6	U62317.3	0.434812135	1.60E-20	postive
IFITM1	U62317.3	0.40734773	5.58E-18	postive
IL15RA	U62317.3	0.5402607	9.57E-33	postive
IL18RAP	U62317.3	0.491563346	1.43E-26	postive
IRF1	U62317.3	0.515666753	1.67E-29	postive
NMI	U62317.3	0.51062958	7.16E-29	postive
RGS1	U62317.3	0.401346667	1.87E-17	postive
TAPBP	U62317.3	0.5072796	1.86E-28	postive
FZD5	AL160314.2	0.59647782	3.00E-41	postive

GPC3	AL160314.2	0.520325993	4.26E-30	postive
KCNMB2	AL160314.2	0.438991679	6.24E-21	postive
SLC11A2	AL160314.2	0.400473018	2.23E-17	postive
ABCA1	AC027243.2	0.431057296	3.67E-20	postive
FZD5	AC027243.2	0.56618147	1.83E-36	postive
KCNMB2	AC027243.2	0.42979048	4.85E-20	postive
ABCA1	AC095055.1	0.414790525	1.20E-18	postive
ABCA1	AL157838.1	0.563857831	4.06E-36	postive
FZD5	AL157838.1	0.479976861	3.04E-25	postive
SLC11A2	AL157838.1	0.464427949	1.54E-23	postive
CD48	TSPOAP1-AS1	0.633299751	8.53E-48	postive
EBI3	TSPOAP1-AS1	0.419228645	4.73E-19	postive
GP1BA	TSPOAP1-AS1	0.63079614	2.53E-47	postive
GPR183	TSPOAP1-AS1	0.470443241	3.45E-24	postive
IL10RA	TSPOAP1-AS1	0.558298944	2.68E-35	postive
KCNA3	TSPOAP1-AS1	0.551437738	2.61E-34	postive
LCK	TSPOAP1-AS1	0.661013747	2.44E-53	postive
LCP2	TSPOAP1-AS1	0.462676042	2.37E-23	postive
LTA	TSPOAP1-AS1	0.698985817	5.71E-62	postive
P2RX7	TSPOAP1-AS1	0.414892833	1.18E-18	postive

PDE4B	TSPOAP1-AS1	0.453329806	2.25E-22	postive
PIK3R5	TSPOAP1-AS1	0.483726992	1.14E-25	postive
SEMA4D	TSPOAP1-AS1	0.405207262	8.61E-18	postive
TNFRSF1B	TSPOAP1-AS1	0.436138566	1.19E-20	postive
TNFRSF9	TSPOAP1-AS1	0.543309343	3.63E-33	postive
IL15	LINC02100	0.4424512	2.84E-21	postive
IRF1	LINC02100	0.455851121	1.23E-22	postive
LCP2	LINC02100	0.434302391	1.79E-20	postive
NMI	LINC02100	0.409036436	3.95E-18	postive
OSMR	LINC02100	0.405510238	8.10E-18	postive
P2RX7	LINC02100	0.411456214	2.40E-18	postive
FZD5	AC024940.5	0.547899081	8.29E-34	postive
KCNMB2	AC024940.5	0.559472827	1.80E-35	postive
ABCA1	PRR34	0.531074724	1.67E-31	postive
BEST1	PRR34	0.484932098	8.33E-26	postive
CYBB	PRR34	0.432137684	2.89E-20	postive
PIK3R5	PRR34	0.421775843	2.75E-19	postive
TLR1	PRR34	0.439146292	6.03E-21	postive
ABCA1	AC024267.3	0.455174656	1.45E-22	postive
FZD5	AC024267.3	0.506972528	2.03E-28	postive

CYBB	AL138995.1	0.487169506	4.61E-26	postive
IL10RA	AL138995.1	0.475448057	9.74E-25	postive
NLRP3	AL138995.1	0.424450001	1.55E-19	postive
PIK3R5	AL138995.1	0.557867473	3.09E-35	postive
SCARF1	AL138995.1	0.416071312	9.20E-19	postive
SCN1B	AL138995.1	0.403388855	1.24E-17	postive
STAB1	AL138995.1	0.689215916	1.27E-59	postive
TACR1	AL138995.1	0.670626637	2.11E-55	postive
TLR1	AL138995.1	0.401529254	1.80E-17	postive
ABCA1	AC012358.1	0.522291225	2.38E-30	postive
ABCA1	WWTR1-IT1	0.719308084	3.59E-67	postive
CYBB	WWTR1-IT1	0.691566316	3.53E-60	postive
IL10RA	WWTR1-IT1	0.407887166	5.00E-18	postive
PIK3R5	WWTR1-IT1	0.458200779	7.02E-23	postive
STAB1	WWTR1-IT1	0.561225813	9.96E-36	postive
TACR1	WWTR1-IT1	0.495157459	5.39E-27	postive
TLR1	WWTR1-IT1	0.691495879	3.67E-60	postive
NAMPT	LINC01697	0.445625649	1.37E-21	postive
TACR1	LINC01697	0.435839767	1.27E-20	postive
ABCA1	AC018521.6	0.488453907	3.28E-26	postive

CALCRL	AC018521.6	0.44902053	6.21E-22	postive
FZD5	AC018521.6	0.638914724	7.14E-49	postive
GPC3	AC018521.6	0.503340861	5.64E-28	postive
KCNMB2	AC018521.6	0.452416707	2.79E-22	postive
SLC11A2	AC018521.6	0.501074351	1.06E-27	postive
FZD5	AC073534.2	0.652015357	1.78E-51	postive
KCNMB2	AC073534.2	0.493354174	8.80E-27	postive
ABCA1	AC100823.1	0.530652665	1.90E-31	postive
CALCRL	AC100823.1	0.442770032	2.64E-21	postive
FZD5	AC100823.1	0.646334049	2.48E-50	postive
GPC3	AC100823.1	0.435504974	1.37E-20	postive
KCNMB2	AC100823.1	0.425418125	1.26E-19	postive
ABCA1	AC005070.3	0.678290429	4.19E-57	postive
FZD5	AC005070.3	0.426906138	9.10E-20	postive
ITGA5	AC005180.1	0.651378191	2.40E-51	postive
MYC	EMSLR	0.62190801	1.12E-45	postive
ABCA1	AC092794.1	0.519934132	4.78E-30	postive
FZD5	AC092794.1	0.404017943	1.09E-17	postive
ABCA1	MIR222HG	0.417813747	6.38E-19	postive
CYBB	MIR222HG	0.479901001	3.10E-25	postive

LPAR1	MIR222HG	0.415979587	9.38E-19	postive
NLRP3	MIR222HG	0.415251469	1.09E-18	postive
TLR1	MIR222HG	0.505039226	3.50E-28	postive
ABCA1	AL117350.1	0.479644388	3.31E-25	postive
AXL	MIR4435-2HG	0.415724349	9.89E-19	postive
C3AR1	MIR4435-2HG	0.440328259	4.61E-21	postive
CD14	MIR4435-2HG	0.476345345	7.74E-25	postive
CD82	MIR4435-2HG	0.402268422	1.56E-17	postive
CMKLR1	MIR4435-2HG	0.478794477	4.13E-25	postive
CSF1	MIR4435-2HG	0.445540784	1.40E-21	postive
EMP3	MIR4435-2HG	0.638706532	7.83E-49	postive
FPR1	MIR4435-2HG	0.422415731	2.40E-19	postive
MMP14	MIR4435-2HG	0.473192778	1.73E-24	postive
PLAUR	MIR4435-2HG	0.528709608	3.44E-31	postive
RHOG	MIR4435-2HG	0.463440016	1.96E-23	postive
SCN1B	MIR4435-2HG	0.563306514	4.91E-36	postive
SPHK1	MIR4435-2HG	0.563080471	5.30E-36	postive
TIMP1	MIR4435-2HG	0.457220089	8.89E-23	postive
ITGA5	AC079313.2	0.712626588	2.06E-65	postive
ABCA1	C21orf62-AS1	0.449162109	6.01E-22	postive

FZD5	C21orf62-AS1	0.577037082	4.02E-38	postive
GPC3	C21orf62-AS1	0.422356625	2.43E-19	postive
IFNAR1	C21orf62-AS1	0.402505513	1.48E-17	postive
KCNMB2	C21orf62-AS1	0.462252496	2.63E-23	postive
SLC11A2	C21orf62-AS1	0.502652856	6.83E-28	postive
ABCA1	AC009268.2	0.451528552	3.45E-22	postive
ABCA1	AC005730.3	0.676318357	1.16E-56	postive
CYBB	AC005730.3	0.402123749	1.60E-17	postive
TLR1	AC005730.3	0.426517851	9.91E-20	postive
FZD5	NDUFB2-AS1	0.501180929	1.03E-27	postive
GPC3	NDUFB2-AS1	0.467716839	6.83E-24	postive
SLC11A2	NDUFB2-AS1	0.401857982	1.69E-17	postive
SCN1B	Z97200.1	0.414328103	1.32E-18	postive
STAB1	AC005332.1	0.454083791	1.88E-22	postive
BEST1	RASSF8-AS1	0.436467749	1.10E-20	postive
C3AR1	RASSF8-AS1	0.404864213	9.23E-18	postive
CMKLR1	RASSF8-AS1	0.422652575	2.28E-19	postive
CSF1	RASSF8-AS1	0.458292573	6.87E-23	postive
EMP3	RASSF8-AS1	0.458549827	6.45E-23	postive
NMUR1	RASSF8-AS1	0.403652863	1.18E-17	postive

PTGER2	RASSF8-AS1	0.418352524	5.69E-19	postive
TIMP1	RASSF8-AS1	0.492883289	1.00E-26	postive
GABBR1	AC087623.1	0.405131616	8.75E-18	postive
KCNJ2	AC087623.1	0.431562372	3.28E-20	postive
MMP14	AC080038.1	0.437720729	8.31E-21	postive
PTGER2	AC080038.1	0.416574609	8.28E-19	postive
SCN1B	AC080038.1	0.416447419	8.50E-19	postive
TIMP1	AC080038.1	0.439799753	5.20E-21	postive
ABCA1	AC015849.3	0.518950913	6.39E-30	postive
ABCA1	AC007785.3	0.414890067	1.18E-18	postive
FZD5	AC007785.3	0.456094146	1.16E-22	postive
ABCA1	AC004637.1	0.655258241	3.86E-52	postive
KIF1B	AC004637.1	0.401315902	1.88E-17	postive
CALCRL	AC016924.1	0.433323926	2.22E-20	postive
FZD5	AC016924.1	0.521132559	3.35E-30	postive
GPC3	AC016924.1	0.711786066	3.41E-65	postive
FZD5	FARP1-AS1	0.506323165	2.44E-28	postive
GPC3	FARP1-AS1	0.482472575	1.59E-25	postive
ABCA1	N4BP2L2-IT2	0.558429529	2.56E-35	postive
FZD5	N4BP2L2-IT2	0.622068751	1.05E-45	postive

KCNMB2	N4BP2L2-IT2	0.482583372	1.54E-25	postive
SLC11A2	N4BP2L2-IT2	0.419769704	4.22E-19	postive
KCNMB2	AC004263.1	0.401105934	1.96E-17	postive
CD40	LINC00173	0.409171097	3.85E-18	postive
CD48	LINC00173	0.524792817	1.12E-30	postive
EBI3	LINC00173	0.560106047	1.46E-35	postive
SLAMF1	LINC00173	0.604827646	1.17E-42	postive
ABCA1	ADAMTSL4-AS2	0.425348502	1.28E-19	postive
ABCA1	AC009754.1	0.554667077	9.00E-35	postive
FZD5	AC009754.1	0.482926857	1.41E-25	postive
KCNMB2	AC009754.1	0.423036717	2.10E-19	postive
MEP1A	AC091179.1	0.926648798	3.72E-177	postive
APLNR	AC147067.2	0.488546841	3.20E-26	postive
CCL2	AC147067.2	0.49575716	4.58E-27	postive
CSF3	AC147067.2	0.404857326	9.25E-18	postive
SCARF1	AC147067.2	0.449262151	5.87E-22	postive
SELE	AC147067.2	0.51389358	2.80E-29	postive
AXL	AL596244.1	0.604914047	1.13E-42	postive
F3	AL596244.1	0.427090316	8.75E-20	postive
HRH1	AL596244.1	0.406057359	7.25E-18	postive

IFITM1	AL596244.1	0.410701221	2.81E-18	postive
LAMP3	AL596244.1	0.406151618	7.11E-18	postive
KIF1B	AC091057.1	0.422539687	2.34E-19	postive
ABCA1	AL391095.3	0.42218506	2.52E-19	postive
CCL24	AL157895.1	0.450576248	4.31E-22	postive
CMKLR1	AL157895.1	0.40305076	1.33E-17	postive
EMP3	AL157895.1	0.42956121	5.10E-20	postive
NMUR1	AL157895.1	0.771391346	6.79E-83	postive
FZD5	AC007128.2	0.519511969	5.41E-30	postive
SLC11A2	AC007128.2	0.472694436	1.96E-24	postive
CD14	AC104051.2	0.409257425	3.78E-18	postive
CSF1	AC104051.2	0.423961872	1.72E-19	postive
CYBB	AC104051.2	0.454101817	1.87E-22	postive
ADGRE1	AC104051.2	0.661862813	1.61E-53	postive
PIK3R5	AC104051.2	0.461764692	2.96E-23	postive
STAB1	AC104051.2	0.609772628	1.63E-43	postive
TACR1	AC104051.2	0.706316763	8.54E-64	postive
ABCA1	AF117829.1	0.543813909	3.09E-33	postive
ABCA1	AC068790.2	0.627424236	1.08E-46	postive
FZD5	AC068790.2	0.529158397	3.00E-31	postive

KCNMB2	AC068790.2	0.447726215	8.40E-22	postive
SLC11A2	AC068790.2	0.408549722	4.37E-18	postive
FZD5	AL358072.1	0.409053309	3.94E-18	postive
BEST1	MAGI2-AS3	0.404126762	1.07E-17	postive
ITGA5	MAGI2-AS3	0.448760239	6.60E-22	postive
ABCA1	GSTCD-AS1	0.647730652	1.31E-50	postive
FZD5	GSTCD-AS1	0.46071032	3.82E-23	postive
ABCA1	AL161891.1	0.449835618	5.13E-22	postive
KIF1B	AL161891.1	0.493331604	8.85E-27	postive
ABCA1	AC008114.1	0.569995198	4.86E-37	postive
FZD5	AC008114.1	0.506614778	2.25E-28	postive
SLC11A2	AC008114.1	0.41770048	6.53E-19	postive
KIF1B	AC145207.5	0.4194766	4.49E-19	postive
ABCA1	AC106037.2	0.658214339	9.41E-53	postive
FZD5	AC106037.2	0.59968986	8.70E-42	postive
GPC3	AC106037.2	0.426408086	1.01E-19	postive
KCNMB2	AC106037.2	0.415775436	9.79E-19	postive
SLC11A2	AC106037.2	0.43202936	2.96E-20	postive
TACR1	AC106037.2	0.405459342	8.19E-18	postive
ABCA1	AC011466.3	0.414609358	1.25E-18	postive

SPHK1	ZNF114-AS1	0.41716071	7.32E-19	postive
SLC28A2	LNCAROD	0.73019807	3.74E-70	postive
ABCA1	AP001793.1	0.499710402	1.55E-27	postive
ABCA1	SPAG5-AS1	0.473790288	1.48E-24	postive
FZD5	SPAG5-AS1	0.595138685	5.00E-41	postive
KCNMB2	SPAG5-AS1	0.456669517	1.01E-22	postive
SLC11A2	SPAG5-AS1	0.455689935	1.28E-22	postive
ABCA1	AC005522.1	0.669176697	4.36E-55	postive
FZD5	AC005522.1	0.552633565	1.76E-34	postive
KCNMB2	AC005522.1	0.456578674	1.04E-22	postive
TACR1	AC005522.1	0.419636268	4.34E-19	postive
ABCA1	AC079921.2	0.637090306	1.61E-48	postive
BEST1	AC079921.2	0.482208775	1.70E-25	postive
C3AR1	AC079921.2	0.41895873	5.01E-19	postive
CMKLR1	AC079921.2	0.466988132	8.18E-24	postive
CSF1	AC079921.2	0.481315096	2.15E-25	postive
CYBB	AC079921.2	0.771107655	8.49E-83	postive
IL10RA	AC079921.2	0.597665702	1.90E-41	postive
LCP2	AC079921.2	0.510331668	7.80E-29	postive
LPAR1	AC079921.2	0.420778119	3.40E-19	postive

MSR1	AC079921.2	0.447486334	8.88E-22	postive
NLRP3	AC079921.2	0.49037586	1.96E-26	postive
OSMR	AC079921.2	0.471965052	2.36E-24	postive
P2RX7	AC079921.2	0.550960627	3.06E-34	postive
PDE4B	AC079921.2	0.408560447	4.36E-18	postive
PIK3R5	AC079921.2	0.65102527	2.83E-51	postive
STAB1	AC079921.2	0.666596208	1.58E-54	postive
TACR1	AC079921.2	0.549495162	4.93E-34	postive
TLR1	AC079921.2	0.79324544	8.47E-91	postive
TLR2	AC079921.2	0.426586873	9.76E-20	postive
CSF1	THCAT158	0.446893714	1.02E-21	postive
EMP3	THCAT158	0.474596503	1.21E-24	postive
ITGA5	THCAT158	0.425308013	1.29E-19	postive
ITGB3	THCAT158	0.613858695	3.12E-44	postive
NMUR1	THCAT158	0.619335045	3.28E-45	postive
TIMP1	THCAT158	0.484224152	1.00E-25	postive
ABCA1	AL022311.1	0.569242048	6.32E-37	postive
FZD5	AL022311.1	0.419452095	4.51E-19	postive
PTPRE	AL163953.1	0.700550252	2.35E-62	postive
SLC7A1	AL163953.1	0.455181315	1.45E-22	postive

MARCO	AC244090.2	0.40149187	1.82E-17	postive
ABCA1	AC009137.2	0.423641688	1.84E-19	postive
ABCA1	C1RL-AS1	0.417466605	6.86E-19	postive
ABCA1	LINC02577	0.405981481	7.36E-18	postive
ADORA2B	LINC02577	0.4019469	1.66E-17	postive
CYBB	LINC02577	0.51913886	6.04E-30	postive
OSMR	LINC02577	0.468866165	5.13E-24	postive
P2RX7	LINC02577	0.403106035	1.32E-17	postive
STAB1	LINC02577	0.426837957	9.24E-20	postive
TLR1	LINC02577	0.526751408	6.23E-31	postive
ABCA1	AC040169.3	0.51650989	1.31E-29	postive
KCNMB2	STAM-AS1	0.413705049	1.51E-18	postive
KCNMB2	AC106820.3	0.587518927	8.80E-40	postive
ABCA1	AC004837.2	0.628934676	5.66E-47	postive
FZD5	AC004837.2	0.509659908	9.45E-29	postive
KIF1B	AC004837.2	0.402937754	1.36E-17	postive
ABCA1	SBF2-AS1	0.416520602	8.37E-19	postive
CYBB	SBF2-AS1	0.465901979	1.07E-23	postive
STAB1	SBF2-AS1	0.447463919	8.93E-22	postive
TACR1	SBF2-AS1	0.48488875	8.43E-26	postive

TLR1	SBF2-AS1	0.482879372	1.43E-25	postive
ABCA1	ZNF775-AS1	0.436246134	1.16E-20	postive
FZD5	ZNF775-AS1	0.463222777	2.07E-23	postive
KCNMB2	ZNF775-AS1	0.54711535	1.07E-33	postive
ABCA1	STARD13-AS	0.719208532	3.81E-67	postive
CYBB	STARD13-AS	0.633384856	8.21E-48	postive
STAB1	STARD13-AS	0.455968911	1.20E-22	postive
TACR1	STARD13-AS	0.447260808	9.36E-22	postive
TLR1	STARD13-AS	0.653730387	7.95E-52	postive
C3AR1	MED14OS	0.400139414	2.38E-17	postive
CMKLR1	MED14OS	0.417488997	6.83E-19	postive
IL10RA	MED14OS	0.421529696	2.90E-19	postive
PIK3R5	MED14OS	0.459086034	5.67E-23	postive
STAB1	MED14OS	0.427448726	8.09E-20	postive
ABCA1	AC138932.4	0.56514392	2.61E-36	postive
FZD5	AC138932.4	0.400201683	2.35E-17	postive
ABCA1	AC002128.1	0.423801759	1.78E-19	postive
FZD5	AC002128.1	0.495072592	5.52E-27	postive
KCNMB2	AC002128.1	0.412887452	1.79E-18	postive
SLC11A2	AC002128.1	0.430376264	4.26E-20	postive

ABCA1	AC006480.2	0.527691839	4.69E-31	postive
ABCA1	AC127024.2	0.568376138	8.55E-37	postive
FZD5	AC084781.1	0.502632994	6.87E-28	postive
ABCA1	CRTC3-AS1	0.403159739	1.30E-17	postive
FZD5	CRTC3-AS1	0.543861435	3.04E-33	postive
KCNMB2	CRTC3-AS1	0.409284031	3.76E-18	postive
SLC11A2	CRTC3-AS1	0.486522571	5.48E-26	postive
ABCA1	AP003171.1	0.581487641	8.07E-39	postive
CYBB	AP003171.1	0.579183366	1.86E-38	postive
TLR1	AP003171.1	0.608793624	2.41E-43	postive
ABCA1	AC027097.2	0.458024705	7.33E-23	postive
BEST1	AC027097.2	0.410482355	2.94E-18	postive
GP1BA	AC027097.2	0.560367836	1.33E-35	postive
GPR183	AC027097.2	0.437348599	9.04E-21	postive
IL10RA	AC027097.2	0.421266378	3.07E-19	postive
KCNA3	AC027097.2	0.444371341	1.83E-21	postive
KCNMB2	AC027097.2	0.416625304	8.19E-19	postive
LTA	AC027097.2	0.433105898	2.33E-20	postive
KCNMB2	AC127024.5	0.400615876	2.16E-17	postive
ACVR2A	MAP3K14-AS1	0.45648119	1.06E-22	postive

ABCA1	HMGA2-AS1	0.621460363	1.35E-45	postive
BEST1	HMGA2-AS1	0.413426191	1.60E-18	postive
CYBB	HMGA2-AS1	0.667122707	1.22E-54	postive
P2RX7	HMGA2-AS1	0.406096863	7.19E-18	postive
PIK3R5	HMGA2-AS1	0.444025355	1.98E-21	postive
STAB1	HMGA2-AS1	0.500492877	1.25E-27	postive
TLR1	HMGA2-AS1	0.68948605	1.10E-59	postive
ABCA1	AC022154.1	0.61158446	7.86E-44	postive
KIF1B	AC022154.1	0.424424956	1.56E-19	postive
STAB1	AC022154.1	0.483921832	1.09E-25	postive
TACR1	AC022154.1	0.496219059	4.04E-27	postive
CD55	AC006213.2	0.414019352	1.41E-18	postive
FZD5	AC027801.1	0.430541781	4.11E-20	postive
ABCA1	AC010132.4	0.48780658	3.90E-26	postive
FZD5	AC010132.4	0.554685394	8.95E-35	postive
KCNMB2	AC010132.4	0.458539063	6.47E-23	postive
SLC11A2	AC010132.4	0.407731699	5.16E-18	postive
ABCA1	AC008121.2	0.401403582	1.85E-17	postive
FZD5	AC008121.2	0.642933419	1.17E-49	postive
KCNMB2	AC008121.2	0.519078919	6.15E-30	postive

SLC11A2	AC008121.2	0.478313551	4.67E-25	postive
KCNMB2	AL442128.2	0.426755985	9.41E-20	postive
ABCA1	AC091982.1	0.476617845	7.22E-25	postive
ABCA1	ODF2-AS1	0.586743929	1.17E-39	postive
FZD5	ODF2-AS1	0.41572268	9.90E-19	postive
ACVR2A	AATBC	0.41088936	2.70E-18	postive
AHR	AATBC	0.412367973	1.99E-18	postive
ABCA1	AL031670.1	0.583397428	4.02E-39	postive
EIF2AK2	AL031670.1	0.431010215	3.71E-20	postive
KIF1B	AL031670.1	0.421839379	2.71E-19	postive
ABCA1	AL121989.1	0.705093464	1.74E-63	postive
FZD5	AL121989.1	0.47419187	1.34E-24	postive
KCNMB2	AL121989.1	0.400807084	2.08E-17	postive
ABCA1	DCUN1D2-AS	0.483833141	1.11E-25	postive
KIF1B	DCUN1D2-AS	0.406018303	7.31E-18	postive
ABCA1	AP001178.1	0.438763578	6.57E-21	postive
FZD5	AP001178.1	0.557583338	3.40E-35	postive
GPC3	AP001178.1	0.414902201	1.18E-18	postive
KCNMB2	AP001178.1	0.401064129	1.98E-17	postive
SLC11A2	AP001178.1	0.42280676	2.21E-19	postive

KCNMB2	FGF14-AS2	0.479104148	3.81E-25	postive
ABCA1	DLEU1	0.560017699	1.50E-35	postive
FZD5	DLEU1	0.644181576	6.64E-50	postive
KCNMB2	DLEU1	0.490709787	1.79E-26	postive
SLC11A2	DLEU1	0.432223664	2.84E-20	postive
FZD5	AC009686.2	0.423262549	2.00E-19	postive
GPC3	AC009686.2	0.420536554	3.58E-19	postive
KCNMB2	AC009686.2	0.409554195	3.55E-18	postive
FZD5	LINC01545	0.50216454	7.83E-28	postive
CD48	AL121820.1	0.400538725	2.20E-17	postive
ABCA1	AC097359.2	0.486337194	5.75E-26	postive
FZD5	AL356481.1	0.45038831	4.51E-22	postive
CXCR6	AC083862.1	0.429809482	4.83E-20	postive
ABCA1	AL450384.1	0.489885633	2.24E-26	postive
FZD5	AL450384.1	0.679431136	2.31E-57	postive
GPC3	AL450384.1	0.401495915	1.82E-17	postive
KCNMB2	AL450384.1	0.488426934	3.30E-26	postive
SLC11A2	AL450384.1	0.425241483	1.31E-19	postive
ABCA1	AC011510.1	0.71921958	3.79E-67	postive
BEST1	AC011510.1	0.421958446	2.65E-19	postive

CYBB	AC011510.1	0.653119999	1.06E-51	postive
PIK3R5	AC011510.1	0.439145588	6.03E-21	postive
STAB1	AC011510.1	0.497091699	3.18E-27	postive
TACR1	AC011510.1	0.480548487	2.62E-25	postive
TLR1	AC011510.1	0.674887583	2.42E-56	postive
KCNMB2	LINC01134	0.436067648	1.21E-20	postive
PROK2	LINC01134	0.460925197	3.63E-23	postive
VIP	LINC01134	0.963829457	1.02E-238	postive
ABCA1	ATP1B3-AS1	0.566859298	1.45E-36	postive
ABCA1	AP001469.2	0.578619119	2.28E-38	postive
KIF1B	AP001469.2	0.433147631	2.31E-20	postive
KIF1B	ZNF8-ERVK3-1	0.529207975	2.96E-31	postive
ABCA1	AC004832.5	0.633513308	7.77E-48	postive
FZD5	AC004832.5	0.546338456	1.37E-33	postive
KCNMB2	AC004832.5	0.401786911	1.71E-17	postive
ABCA1	GRK5-IT1	0.777392573	5.64E-85	postive
CYBB	GRK5-IT1	0.577994747	2.85E-38	postive
KIF1B	GRK5-IT1	0.416459873	8.48E-19	postive
STAB1	GRK5-IT1	0.446085226	1.23E-21	postive
TACR1	GRK5-IT1	0.478579119	4.36E-25	postive

TLR1	GRK5-IT1	0.591118081	2.29E-40	postive
ABCA1	AL139021.2	0.413528636	1.56E-18	postive
FZD5	AL139021.2	0.557574	3.41E-35	postive
SLC11A2	AL139021.2	0.457954829	7.45E-23	postive
APLNR	AC093278.2	0.624675634	3.49E-46	postive
BEST1	AC093278.2	0.413828971	1.47E-18	postive
CALCRL	AC093278.2	0.69552889	3.96E-61	postive
PIK3R5	AC093278.2	0.417729609	6.49E-19	postive
PTGER2	AC093278.2	0.408464486	4.44E-18	postive
PTGIR	AC093278.2	0.415526542	1.03E-18	postive
SCARF1	AC093278.2	0.672616372	7.70E-56	postive
CYBB	LINC01203	0.432292658	2.79E-20	postive
TLR1	LINC01203	0.435724658	1.30E-20	postive
ABCA1	AL162724.2	0.707323851	4.75E-64	postive
BEST1	AL162724.2	0.455724572	1.27E-22	postive
CYBB	AL162724.2	0.711178825	4.89E-65	postive
IL10RA	AL162724.2	0.477419875	5.88E-25	postive
P2RX7	AL162724.2	0.424711266	1.46E-19	postive
PIK3R5	AL162724.2	0.520395276	4.17E-30	postive
STAB1	AL162724.2	0.592752517	1.24E-40	postive

TACR1	AL162724.2	0.52730865	5.27E-31	postive
TLR1	AL162724.2	0.710223475	8.62E-65	postive
ABCA1	NARF-AS1	0.418002274	6.13E-19	postive
SLC11A2	UBL7-AS1	0.401699634	1.74E-17	postive
KCNMB2	AL139286.1	0.502214338	7.72E-28	postive
ABCA1	AC087301.1	0.453432938	2.20E-22	postive
ABCA1	AC004951.1	0.598778617	1.24E-41	postive
FZD5	AC004951.1	0.419753535	4.23E-19	postive
BTG2	AL133355.1	0.434250169	1.81E-20	postive
FZD5	DTX2P1-UPK3BP1-PMS2P11	0.411873211	2.20E-18	postive
ABCA1	AC078852.1	0.623688327	5.30E-46	postive
FZD5	AC078852.1	0.584491339	2.69E-39	postive
KCNMB2	AC078852.1	0.48631551	5.79E-26	postive
FZD5	AL445231.1	0.523920656	1.46E-30	postive
SLC11A2	AL445231.1	0.405015994	8.95E-18	postive
ACVR2A	ZNF32-AS2	0.415348591	1.07E-18	postive
RHOG	ZNF32-AS2	-0.400542016	2.20E-17	negative
PTPRE	LINC01405	0.52718572	5.46E-31	postive
SLC7A1	LINC01405	0.42229434	2.46E-19	postive
ABCA1	AC066613.1	0.636903928	1.75E-48	postive

ABCA1	AC012085.2	0.452254893	2.90E-22	postive
FZD5	AC012085.2	0.434822231	1.59E-20	postive
ABCA1	MIR302CHG	0.550720052	3.31E-34	postive
FZD5	MIR302CHG	0.455255628	1.42E-22	postive
SLC11A2	MIR302CHG	0.401063254	1.98E-17	postive
ABCA1	AP001033.2	0.404355134	1.02E-17	postive
ABCA1	ANKRD44-IT1	0.668713342	5.50E-55	postive
CYBB	ANKRD44-IT1	0.645785643	3.19E-50	postive
STAB1	ANKRD44-IT1	0.450463073	4.43E-22	postive
TACR1	ANKRD44-IT1	0.413203153	1.67E-18	postive
TLR1	ANKRD44-IT1	0.667886433	8.31E-55	postive
ACVR2A	AC010761.4	0.426466544	1.00E-19	postive
ABCA1	AC108463.2	0.729893692	4.55E-70	postive
AXL	AC108463.2	0.40004129	2.43E-17	postive
BEST1	AC108463.2	0.449398079	5.69E-22	postive
CALCRL	AC108463.2	0.533878318	7.05E-32	postive
CMKLR1	AC108463.2	0.432935621	2.42E-20	postive
CYBB	AC108463.2	0.559698778	1.67E-35	postive
IL10RA	AC108463.2	0.430420157	4.22E-20	postive
ITGB3	AC108463.2	0.400163985	2.37E-17	postive

NLRP3	AC108463.2	0.405922648	7.45E-18	postive
PIK3R5	AC108463.2	0.494138519	7.12E-27	postive
SCARF1	AC108463.2	0.489264109	2.64E-26	postive
STAB1	AC108463.2	0.557288043	3.76E-35	postive
TACR1	AC108463.2	0.545771805	1.65E-33	postive
TLR1	AC108463.2	0.530231127	2.16E-31	postive
ABCA1	AC024267.5	0.736263748	7.05E-72	postive
FZD5	AC024267.5	0.507332309	1.83E-28	postive
KCNMB2	AC024267.5	0.401130242	1.95E-17	postive
TACR1	AC024267.5	0.426657592	9.61E-20	postive
ABCA1	AC005899.7	0.462576063	2.43E-23	postive
FZD5	AC005899.7	0.463886329	1.76E-23	postive
KCNMB2	AC005899.7	0.442214317	3.00E-21	postive
ABCA1	AC009087.1	0.402713829	1.42E-17	postive
BEST1	LINC00426	0.416124712	9.10E-19	postive
C3AR1	LINC00426	0.458856075	5.99E-23	postive
C5AR1	LINC00426	0.428331662	6.67E-20	postive
CD40	LINC00426	0.401243433	1.91E-17	postive
CD48	LINC00426	0.830903702	6.01E-107	postive
CXCL9	LINC00426	0.533849504	7.11E-32	postive

CXCR6	LINC00426	0.594886901	5.51E-41	postive
CYBB	LINC00426	0.486445682	5.59E-26	postive
EBI3	LINC00426	0.548139879	7.67E-34	postive
GP1BA	LINC00426	0.707760595	3.68E-64	postive
GPR132	LINC00426	0.609404016	1.89E-43	postive
GPR183	LINC00426	0.656142734	2.53E-52	postive
IL10RA	LINC00426	0.745260362	1.59E-74	postive
IL18RAP	LINC00426	0.523257655	1.78E-30	postive
IL2RB	LINC00426	0.534007118	6.77E-32	postive
IL7R	LINC00426	0.499889114	1.47E-27	postive
KCNA3	LINC00426	0.724268497	1.64E-68	postive
LCK	LINC00426	0.919189402	7.79E-169	postive
LCP2	LINC00426	0.707723664	3.76E-64	postive
LTA	LINC00426	0.885974685	1.41E-139	postive
P2RX7	LINC00426	0.536627396	3.00E-32	postive
PDE4B	LINC00426	0.543053488	3.94E-33	postive
PIK3R5	LINC00426	0.631003416	2.32E-47	postive
PTAFR	LINC00426	0.452193433	2.95E-22	postive
PTGIR	LINC00426	0.40635021	6.83E-18	postive
RASGRP1	LINC00426	0.51047076	7.50E-29	postive

RGS1	LINC00426	0.615005133	1.96E-44	postive
SEMA4D	LINC00426	0.458938826	5.88E-23	postive
SLAMF1	LINC00426	0.482779088	1.47E-25	postive
TNFRSF1B	LINC00426	0.607409608	4.19E-43	postive
TNFRSF9	LINC00426	0.675065956	2.21E-56	postive
ABCA1	ADAMTSL4-AS1	0.753313338	5.41E-77	postive
CYBB	ADAMTSL4-AS1	0.41711961	7.38E-19	postive
KIF1B	ADAMTSL4-AS1	0.412251921	2.04E-18	postive
STAB1	ADAMTSL4-AS1	0.419619016	4.35E-19	postive
TACR1	ADAMTSL4-AS1	0.471468492	2.67E-24	postive
ABCA1	AC211433.1	0.614058734	2.88E-44	postive
FZD5	AC211433.1	0.543494097	3.42E-33	postive
KCNMB2	AC211433.1	0.454981012	1.52E-22	postive
ABCA1	AC092171.1	0.702302186	8.67E-63	postive
FZD5	AC092171.1	0.432288904	2.80E-20	postive
KIF1B	AC092171.1	0.406834112	6.19E-18	postive
TLR1	AC092171.1	0.406499925	6.63E-18	postive
FZD5	AC068768.1	0.407822167	5.07E-18	postive
ABCA1	AC104984.2	0.704419831	2.57E-63	postive
KCNMB2	BX284668.5	0.468834318	5.17E-24	postive

KCNMB2	IPO9-AS1	0.565903228	2.01E-36	postive
SGMS2	AP001412.1	-0.402624926	1.45E-17	negative
CD48	RABGAP1L-DT	0.405112987	8.78E-18	postive
EBI3	RABGAP1L-DT	0.420709283	3.45E-19	postive
SLAMF1	RABGAP1L-DT	0.489578725	2.43E-26	postive
FFAR2	LNC-LBCS	0.530924076	1.75E-31	postive
ABCA1	AC020891.3	0.548434806	6.97E-34	postive
ABCA1	AC145423.2	0.467673268	6.90E-24	postive
ABCA1	AC068790.5	0.71631812	2.23E-66	postive
FZD5	AC068790.5	0.468619172	5.45E-24	postive
TACR1	AC068790.5	0.415821661	9.70E-19	postive
FZD5	AC010175.1	0.45269454	2.62E-22	postive
OSMR	AC020663.2	-0.414379621	1.31E-18	negative
ABCA1	AC011442.1	0.5421585	5.24E-33	postive
FZD5	AC011442.1	0.497526315	2.82E-27	postive
SLC11A2	AC011442.1	0.46738456	7.42E-24	postive
KCNMB2	SAP30-DT	0.437128941	9.50E-21	postive
ABCA1	SDCBP2-AS1	0.693560098	1.18E-60	postive
KIF1B	SDCBP2-AS1	0.409707903	3.44E-18	postive
TLR1	SDCBP2-AS1	0.432010182	2.97E-20	postive

IL15	AL157394.1	0.44401913	1.98E-21	postive
IL15RA	AL157394.1	0.444461656	1.79E-21	postive
LCP2	AL157394.1	0.403672202	1.17E-17	postive
PTAFR	AL157394.1	0.409877947	3.33E-18	postive
RGS1	AL157394.1	0.400251614	2.33E-17	postive
TLR1	AL157394.1	0.464650806	1.46E-23	postive
FPR1	AL354811.1	0.488442899	3.29E-26	postive
ITGA5	AL354811.1	0.404447441	1.00E-17	postive
PROK2	AL354811.1	0.502936888	6.31E-28	postive
SCN1B	AL354811.1	0.458353228	6.77E-23	postive
CXCL10	AL357054.4	0.416458187	8.48E-19	postive
CXCR6	AL357054.4	0.403798769	1.14E-17	postive
IL15	AL357054.4	0.446492631	1.12E-21	postive
IL15RA	AL357054.4	0.442631905	2.72E-21	postive
IL18RAP	AL357054.4	0.432712173	2.55E-20	postive
IRF1	AL357054.4	0.560899422	1.11E-35	postive
LCP2	AL357054.4	0.434395316	1.75E-20	postive
NMI	AL357054.4	0.489729643	2.33E-26	postive
PTAFR	AL357054.4	0.421256154	3.07E-19	postive
RTP4	AL357054.4	0.424104981	1.67E-19	postive

TAPBP	AL357054.4	0.432653676	2.58E-20	postive
HPN	RAMP2-AS1	0.412922512	1.77E-18	postive
SPHK1	AC011944.1	0.407640994	5.26E-18	postive
ITGA5	AL162424.1	0.482543517	1.56E-25	postive
PTGER2	MAP3K4-AS1	0.498325927	2.27E-27	postive
TNFAIP6	MAP3K4-AS1	0.417486298	6.83E-19	postive
ABCA1	AL355102.1	0.670326194	2.45E-55	postive
CYBB	AL355102.1	0.687539714	3.15E-59	postive
PIK3R5	AL355102.1	0.436905725	9.99E-21	postive
STAB1	AL355102.1	0.529580645	2.64E-31	postive
TACR1	AL355102.1	0.455576966	1.32E-22	postive
TLR1	AL355102.1	0.697293387	1.48E-61	postive
ACVR1B	AC121338.2	0.558134907	2.83E-35	postive
MEP1A	AC121338.2	0.556272512	5.28E-35	postive
PTPRE	LINC02178	0.534066449	6.65E-32	postive
SLC7A1	LINC02178	0.431390922	3.41E-20	postive
ABCA1	TXNDC12-AS1	0.672690909	7.42E-56	postive
FZD5	TXNDC12-AS1	0.499030672	1.87E-27	postive
KCNMB2	TXNDC12-AS1	0.409795927	3.38E-18	postive
ABCA1	AC097634.1	0.464006928	1.71E-23	postive

ABCA1	AL117336.1	0.459430762	5.22E-23	postive
BEST1	AL117336.1	0.417270968	7.15E-19	postive
CYBB	AL117336.1	0.434823066	1.59E-20	postive
GPR183	AL117336.1	0.62769703	9.63E-47	postive
IL10RA	AL117336.1	0.445885065	1.29E-21	postive
KCNA3	AL117336.1	0.457132821	9.08E-23	postive
LCP2	AL117336.1	0.414930183	1.17E-18	postive
PDE4B	AL117336.1	0.49153849	1.44E-26	postive
PIK3R5	AL117336.1	0.41425209	1.35E-18	postive
TLR1	AL117336.1	0.432562673	2.63E-20	postive
MSR1	AC067817.2	0.410967592	2.66E-18	postive
FZD5	AL021707.4	0.435408604	1.40E-20	postive
SLC11A2	AL021707.4	0.4007705	2.10E-17	postive
FZD5	AC021321.1	0.518398992	7.51E-30	postive
KCNMB2	AC021321.1	0.403048991	1.33E-17	postive
ABCA1	TTC3-AS1	0.534791139	5.31E-32	postive
FZD5	LINC02340	0.533154877	8.81E-32	postive
GPC3	LINC02340	0.545504266	1.80E-33	postive
SLC11A2	LINC02340	0.411658343	2.31E-18	postive
ABCA1	LINC02042	0.463714247	1.84E-23	postive

FZD5	LINC02042	0.65402692	6.91E-52	postive
KCNMB2	LINC02042	0.509202444	1.08E-28	postive
SLC11A2	LINC02042	0.408501953	4.41E-18	postive
FZD5	AL731569.1	0.428137928	6.96E-20	postive
KCNMB2	AL731569.1	0.491823425	1.33E-26	postive
ABCA1	AC073487.1	0.693941104	9.56E-61	postive
FZD5	AC073487.1	0.431049286	3.68E-20	postive
TACR1	AC073487.1	0.445974606	1.26E-21	postive
ITGA5	AP001107.5	0.443097533	2.45E-21	postive
ABCA1	DLEU2	0.492683476	1.06E-26	postive
ABCA1	AC008870.4	0.572711146	1.87E-37	postive
FZD5	AC008870.4	0.413076549	1.72E-18	postive
SLC11A2	AC008870.4	0.431762749	3.14E-20	postive
P2RY2	AP002761.4	0.740644355	3.74E-73	postive
ABCA1	AC007619.1	0.599316251	1.01E-41	postive
ABCA1	LRP4-AS1	0.437490687	8.76E-21	postive
FZD5	LRP4-AS1	0.421694491	2.80E-19	postive
KCNMB2	LRP4-AS1	0.453166275	2.34E-22	postive
ABCA1	AC012435.3	0.465130879	1.30E-23	postive
ABCA1	MAL2-AS1	0.457974732	7.41E-23	postive

FZD5	MAL2-AS1	0.680581683	1.27E-57	postive
GPC3	MAL2-AS1	0.404390946	1.02E-17	postive
KCNMB2	MAL2-AS1	0.511323955	5.87E-29	postive
SLC11A2	MAL2-AS1	0.439922227	5.05E-21	postive
C3AR1	AC006369.1	0.5008692	1.12E-27	postive
C5AR1	AC006369.1	0.401711683	1.74E-17	postive
CD48	AC006369.1	0.605773981	8.03E-43	postive
CXCL9	AC006369.1	0.59136039	2.09E-40	postive
CXCR6	AC006369.1	0.628333632	7.33E-47	postive
EBI3	AC006369.1	0.400162683	2.37E-17	postive
GP1BA	AC006369.1	0.476752584	6.97E-25	postive
GPR132	AC006369.1	0.56950193	5.78E-37	postive
GPR183	AC006369.1	0.518408529	7.49E-30	postive
IL10RA	AC006369.1	0.630761415	2.57E-47	postive
IL18RAP	AC006369.1	0.508207117	1.43E-28	postive
IL2RB	AC006369.1	0.529868892	2.42E-31	postive
IL7R	AC006369.1	0.457700562	7.92E-23	postive
KCNA3	AC006369.1	0.613123913	4.21E-44	postive
LCK	AC006369.1	0.750194667	5.01E-76	postive
LCP2	AC006369.1	0.659540499	4.97E-53	postive

LTA	AC006369.1	0.69305054	1.56E-60	postive
P2RX7	AC006369.1	0.495435688	5.00E-27	postive
PDE4B	AC006369.1	0.447605094	8.64E-22	postive
PIK3R5	AC006369.1	0.524658372	1.17E-30	postive
RASGRP1	AC006369.1	0.426562745	9.81E-20	postive
RGS1	AC006369.1	0.630354201	3.07E-47	postive
TNFRSF1B	AC006369.1	0.576275535	5.28E-38	postive
TNFRSF9	AC006369.1	0.62358417	5.53E-46	postive
ABCA1	AC130324.1	0.425701951	1.18E-19	postive
APLNR	LINC02285	0.507532578	1.73E-28	postive
BEST1	LINC02285	0.53319373	8.71E-32	postive
C3AR1	LINC02285	0.622303911	9.49E-46	postive
C5AR1	LINC02285	0.545086691	2.06E-33	postive
CD14	LINC02285	0.717916153	8.42E-67	postive
CD48	LINC02285	0.538697831	1.57E-32	postive
CMKLR1	LINC02285	0.703869716	3.52E-63	postive
CSF1	LINC02285	0.637185782	1.54E-48	postive
CYBB	LINC02285	0.625796188	2.17E-46	postive
EBI3	LINC02285	0.403218655	1.29E-17	postive
EMP3	LINC02285	0.590672373	2.71E-40	postive

ADGRE1	LINC02285	0.463835823	1.78E-23	postive
FPR1	LINC02285	0.580641679	1.10E-38	postive
GP1BA	LINC02285	0.466238316	9.85E-24	postive
GPR132	LINC02285	0.491871859	1.31E-26	postive
GPR183	LINC02285	0.505485048	3.09E-28	postive
IL10RA	LINC02285	0.750709305	3.48E-76	postive
IL7R	LINC02285	0.42379215	1.79E-19	postive
KCNA3	LINC02285	0.477518188	5.73E-25	postive
LCK	LINC02285	0.580080035	1.35E-38	postive
LCP2	LINC02285	0.613839484	3.15E-44	postive
LTA	LINC02285	0.61649557	1.06E-44	postive
MARCO	LINC02285	0.404317051	1.03E-17	postive
MSR1	LINC02285	0.464377521	1.56E-23	postive
NLRP3	LINC02285	0.508646929	1.26E-28	postive
P2RX7	LINC02285	0.461771705	2.95E-23	postive
PDE4B	LINC02285	0.450329961	4.57E-22	postive
PIK3R5	LINC02285	0.71046967	7.45E-65	postive
PTAFR	LINC02285	0.415894814	9.55E-19	postive
PTGIR	LINC02285	0.478371007	4.60E-25	postive
RGS1	LINC02285	0.488645103	3.12E-26	postive

RHOG	LINC02285	0.563130877	5.21E-36	postive
SCARF1	LINC02285	0.42540237	1.26E-19	postive
SCN1B	LINC02285	0.617729226	6.38E-45	postive
STAB1	LINC02285	0.570407969	4.21E-37	postive
TACR1	LINC02285	0.505486177	3.09E-28	postive
TIMP1	LINC02285	0.50709554	1.96E-28	postive
TLR1	LINC02285	0.473539931	1.58E-24	postive
TNFRSF1B	LINC02285	0.657525078	1.31E-52	postive
TNFRSF9	LINC02285	0.490067644	2.13E-26	postive
ATP2B1	ATP2B1-AS1	0.596695795	2.76E-41	postive
ABCA1	AC099811.5	0.744007932	3.76E-74	postive
CYBB	AC099811.5	0.413700666	1.51E-18	postive
KIF1B	AC099811.5	0.429360583	5.33E-20	postive
TLR1	AC099811.5	0.432057842	2.94E-20	postive
FZD5	AC005225.2	0.669628561	3.48E-55	postive
GPC3	AC005225.2	0.438281395	7.33E-21	postive
KCNMB2	AC005225.2	0.432662625	2.57E-20	postive
ABCA1	ANKRD10-IT1	0.43316587	2.30E-20	postive
ABCA1	AP001630.1	0.429264155	5.44E-20	postive
CALCRL	AP001630.1	0.403753519	1.15E-17	postive

FZD5	AP001630.1	0.705165028	1.67E-63	postive
GPC3	AP001630.1	0.468331714	5.86E-24	postive
KCNMB2	AP001630.1	0.498926277	1.92E-27	postive
SLC11A2	AP001630.1	0.46117393	3.42E-23	postive
APLNR	AL109741.1	0.580232067	1.27E-38	postive
BDKRB1	AL109741.1	0.450319783	4.58E-22	postive
BEST1	AL109741.1	0.433481251	2.15E-20	postive
C5AR1	AL109741.1	0.510553557	7.32E-29	postive
CALCRL	AL109741.1	0.48811117	3.59E-26	postive
CCL2	AL109741.1	0.496021008	4.26E-27	postive
FPR1	AL109741.1	0.492149242	1.22E-26	postive
GPR183	AL109741.1	0.50850555	1.31E-28	postive
IL10RA	AL109741.1	0.430247813	4.39E-20	postive
IL6	AL109741.1	0.503619562	5.22E-28	postive
ITGA5	AL109741.1	0.497581734	2.78E-27	postive
KCNA3	AL109741.1	0.444057571	1.96E-21	postive
LIF	AL109741.1	0.42182494	2.72E-19	postive
NLRP3	AL109741.1	0.422511044	2.35E-19	postive
PDE4B	AL109741.1	0.553961602	1.14E-34	postive
PIK3R5	AL109741.1	0.437981998	7.84E-21	postive

PTGER2	AL109741.1	0.481438059	2.08E-25	postive
SCARF1	AL109741.1	0.531513521	1.46E-31	postive
TNFAIP6	AL109741.1	0.475381111	9.90E-25	postive
TNFRSF1B	AL109741.1	0.439479427	5.59E-21	postive
SELL	AP006545.2	0.66058411	3.00E-53	postive
ABCA1	AL158166.2	0.544926228	2.16E-33	postive
PTPRE	AL158166.2	0.610431979	1.25E-43	postive
ABCA1	AC078846.1	0.544698285	2.33E-33	postive
KCNMB2	AC078846.1	0.429905092	4.73E-20	postive
MEP1A	AC104534.1	0.923590697	1.21E-173	postive
AHR	PSORS1C3	0.421228291	3.09E-19	postive
KIF1B	AC099850.3	0.542913839	4.12E-33	postive
SLC31A1	AC099850.3	0.408440731	4.46E-18	postive
ABCA1	AC007681.1	0.503951706	4.75E-28	postive
CALCRL	AC007681.1	0.52283273	2.02E-30	postive
FZD5	AC007681.1	0.629305566	4.83E-47	postive
GPC3	AC007681.1	0.512530893	4.15E-29	postive
SLC11A2	AC007681.1	0.487685791	4.02E-26	postive
TLR1	MSC-AS1	0.419723814	4.26E-19	postive
ABCA1	AC108449.2	0.72729428	2.41E-69	postive

CALCRL	AC108449.2	0.409992309	3.25E-18	postive
FZD5	AC108449.2	0.534326661	6.13E-32	postive
KIF1B	AC108449.2	0.528858808	3.29E-31	postive
SLC11A2	AC108449.2	0.43558875	1.34E-20	postive
TACR1	AC108449.2	0.415746771	9.85E-19	postive
ABCA1	AC006566.1	0.466759008	8.66E-24	postive
FZD5	AC006566.1	0.636308023	2.27E-48	postive
KCNMB2	AC006566.1	0.475040216	1.08E-24	postive
SLC11A2	AC006566.1	0.430709988	3.96E-20	postive
FZD5	AC025682.1	0.429776764	4.86E-20	postive
SLC11A2	AC025682.1	0.407769859	5.12E-18	postive
AHR	AC109460.2	0.407265409	5.67E-18	postive
AQP9	SUGCT-AS1	0.514258856	2.52E-29	postive
C3AR1	SUGCT-AS1	0.563432457	4.70E-36	postive
C5AR1	SUGCT-AS1	0.518846452	6.59E-30	postive
CMKLR1	SUGCT-AS1	0.459889424	4.67E-23	postive
FPR1	SUGCT-AS1	0.407098057	5.87E-18	postive
GPR183	SUGCT-AS1	0.425043203	1.36E-19	postive
IL10RA	SUGCT-AS1	0.452410087	2.80E-22	postive
KCNA3	SUGCT-AS1	0.4425106	2.80E-21	postive

LCP2	SUGCT-AS1	0.479172889	3.74E-25	postive
MSR1	SUGCT-AS1	0.462297879	2.60E-23	postive
NLRP3	SUGCT-AS1	0.422007617	2.62E-19	postive
OSM	SUGCT-AS1	0.412091915	2.11E-18	postive
P2RX7	SUGCT-AS1	0.463343311	2.01E-23	postive
PIK3R5	SUGCT-AS1	0.402157996	1.59E-17	postive
PLAUR	SUGCT-AS1	0.427196175	8.55E-20	postive
PTAFR	SUGCT-AS1	0.426170099	1.07E-19	postive
PTGIR	SUGCT-AS1	0.412799862	1.82E-18	postive
RGS1	SUGCT-AS1	0.482240197	1.69E-25	postive
ABCA1	AC021087.3	0.437874653	8.03E-21	postive
FZD5	AC021087.3	0.504020935	4.66E-28	postive
KCNMB2	AC021087.3	0.42475585	1.45E-19	postive
ABCA1	MAP3K20-AS1	0.672618655	7.69E-56	postive
CYBB	MAP3K20-AS1	0.656455423	2.18E-52	postive
IL10RA	MAP3K20-AS1	0.430093715	4.54E-20	postive
KIF1B	MAP3K20-AS1	0.402869446	1.38E-17	postive
NLRP3	MAP3K20-AS1	0.429475229	5.20E-20	postive
PIK3R5	MAP3K20-AS1	0.492673862	1.06E-26	postive
STAB1	MAP3K20-AS1	0.569449494	5.88E-37	postive

TACR1	MAP3K20-AS1	0.488526357	3.22E-26	postive
TLR1	MAP3K20-AS1	0.632594258	1.16E-47	postive
ACVR2A	AL365330.1	0.414007688	1.42E-18	postive
ABCA1	AC137932.3	0.438890483	6.38E-21	postive
BEST1	AC009093.6	0.430568629	4.09E-20	postive
EMP3	AC009093.6	0.414690809	1.23E-18	postive
FPR1	AC009093.6	0.42454352	1.52E-19	postive
GPR183	AC009093.6	0.471500296	2.65E-24	postive
IL10RA	AC009093.6	0.428482864	6.46E-20	postive
IL18RAP	AC009093.6	0.409617008	3.51E-18	postive
LCP2	AC009093.6	0.408633697	4.29E-18	postive
PIK3R5	AC009093.6	0.404291913	1.04E-17	postive
PTGIR	AC009093.6	0.402277282	1.55E-17	postive
SCN1B	AC009093.6	0.439700649	5.31E-21	postive
TNFRSF1B	AC009093.6	0.423025771	2.10E-19	postive
ABCA1	ARAP1-AS2	0.751246269	2.38E-76	postive
CYBB	ARAP1-AS2	0.449611916	5.41E-22	postive
KIF1B	ARAP1-AS2	0.431371972	3.42E-20	postive
STAB1	ARAP1-AS2	0.404291598	1.04E-17	postive
TACR1	ARAP1-AS2	0.442121232	3.06E-21	postive

TLR1	ARAP1-AS2	0.445176144	1.52E-21	postive
ABCA1	AC104938.1	0.533333856	8.34E-32	postive
PIK3R5	AC104938.1	0.403168498	1.30E-17	postive
STAB1	AC104938.1	0.615866726	1.37E-44	postive
TACR1	AC104938.1	0.550785081	3.24E-34	postive
ABCA1	AC245884.9	0.423304125	1.98E-19	postive
FZD5	AC245884.9	0.456779717	9.88E-23	postive
ABCA1	FBXO30-DT	0.485918216	6.43E-26	postive
ABCA1	AC022165.1	0.519304836	5.75E-30	postive
FZD5	AC022165.1	0.495536901	4.86E-27	postive
SLC11A2	AC022165.1	0.401783024	1.71E-17	postive
KCNMB2	AC011815.2	0.458587454	6.40E-23	postive
ABCA1	AL592430.1	0.486295182	5.82E-26	postive
FZD5	AL592430.1	0.669718234	3.32E-55	postive
GPC3	AL592430.1	0.412766802	1.83E-18	postive
KCNMB2	AL592430.1	0.494273523	6.86E-27	postive
SLC11A2	AL592430.1	0.456277127	1.11E-22	postive
KCNMB2	AL356489.2	0.582922552	4.79E-39	postive
VIP	AL356489.2	0.493603264	8.23E-27	postive
ABCA1	AC092828.1	0.535028244	4.93E-32	postive

FZD5	AC092828.1	0.645447946	3.73E-50	postive
KCNMB2	AC092828.1	0.512688007	3.96E-29	postive
SLC11A2	AC092828.1	0.424113173	1.67E-19	postive
CD55	AC062028.1	0.635903994	2.72E-48	postive
AXL	AC009549.1	0.461000352	3.56E-23	postive
SERPINE1	AC009549.1	0.43778296	8.20E-21	postive
ABCA1	AP001020.3	0.640156497	4.09E-49	postive
FZD5	AP001020.3	0.645744597	3.26E-50	postive
KCNMB2	AP001020.3	0.476595053	7.26E-25	postive
SLC11A2	AP001020.3	0.43700652	9.76E-21	postive
FZD5	AC021491.2	0.632262056	1.34E-47	postive
KCNMB2	AC021491.2	0.429088136	5.66E-20	postive
SLC11A2	AC021491.2	0.456725914	1.00E-22	postive
ABCA1	AL596223.1	0.602497632	2.92E-42	postive
CMKLR1	AL596223.1	0.40948642	3.60E-18	postive
CSF1	AL596223.1	0.424409414	1.56E-19	postive
CYBB	AL596223.1	0.714700633	5.94E-66	postive
ADGRE1	AL596223.1	0.480119831	2.93E-25	postive
IL10RA	AL596223.1	0.517415971	1.00E-29	postive
NLRP3	AL596223.1	0.437600764	8.54E-21	postive

PIK3R5	AL596223.1	0.630769254	2.56E-47	postive
STAB1	AL596223.1	0.87381752	4.37E-131	postive
TACR1	AL596223.1	0.77817924	2.98E-85	postive
TLR1	AL596223.1	0.618059453	5.57E-45	postive
ABCA1	AC025287.3	0.473486452	1.60E-24	postive
EMP3	AC134312.5	0.45643652	1.07E-22	postive
MMP14	AC134312.5	0.652338198	1.53E-51	postive
SCN1B	AC134312.5	0.491418633	1.48E-26	postive
SPHK1	AC134312.5	0.420507201	3.61E-19	postive
ABCA1	AC005828.1	0.519644827	5.21E-30	postive
FZD5	AC005828.1	0.630712739	2.63E-47	postive
KCNMB2	AC005828.1	0.506010093	2.66E-28	postive
SLC11A2	AC005828.1	0.422260553	2.48E-19	postive
FZD5	AC106771.1	0.478611483	4.32E-25	postive
GPC3	AC106771.1	0.464733637	1.43E-23	postive
VIP	DLX6-AS1	0.422939417	2.14E-19	postive
ABCA1	AC093423.2	0.730350735	3.39E-70	postive
CYBB	AC093423.2	0.602879575	2.51E-42	postive
KIF1B	AC093423.2	0.407009271	5.98E-18	postive
STAB1	AC093423.2	0.421416819	2.97E-19	postive

TACR1	AC093423.2	0.454563273	1.68E-22	postive
TLR1	AC093423.2	0.631720867	1.70E-47	postive
VIP	AL158211.1	0.40881214	4.14E-18	postive
ABCA1	AC084880.3	0.459429431	5.22E-23	postive
MXD1	AC084880.3	0.409125862	3.88E-18	postive
ITGA5	AC013553.3	0.444183918	1.91E-21	postive
STAB1	AC013553.3	0.421835111	2.72E-19	postive
TACR1	AC013553.3	0.462668145	2.37E-23	postive
ABCA1	AP001107.1	0.607116559	4.71E-43	postive
KCNMB2	LINC01550	0.568381677	8.53E-37	postive
PROK2	LINC01550	0.435230205	1.45E-20	postive
VIP	LINC01550	0.776212884	1.46E-84	postive
SLC28A2	AC025165.5	0.456525172	1.05E-22	postive
NOD2	TM4SF19-AS1	0.423620313	1.85E-19	postive
FZD5	AC079160.1	0.620120378	2.37E-45	postive
SLC11A2	AC079160.1	0.407858189	5.03E-18	postive
ABCA1	AC040904.1	0.678668049	3.44E-57	postive
CYBB	AC040904.1	0.567467935	1.17E-36	postive
TLR1	AC040904.1	0.596756101	2.70E-41	postive
CD40	AL117335.1	0.487873747	3.83E-26	postive

CD48	AL117335.1	0.556764989	4.48E-35	postive
EBI3	AL117335.1	0.686331339	6.02E-59	postive
PTGIR	AL117335.1	0.411447144	2.41E-18	postive
RASGRP1	AL117335.1	0.411275063	2.49E-18	postive
SLAMF1	AL117335.1	0.770733841	1.14E-82	postive
ABCA1	AL354726.1	0.701097012	1.73E-62	postive
FZD5	AL354726.1	0.44262352	2.73E-21	postive
KIF1B	AL354726.1	0.409567563	3.54E-18	postive
FZD5	AC024361.1	0.530462916	2.02E-31	postive
KCNMB2	AC024361.1	0.42552054	1.23E-19	postive
SLC11A2	AC024361.1	0.449647804	5.36E-22	postive
ABCA1	AC108727.1	0.666607136	1.57E-54	postive
IRF7	AL357033.4	0.425015632	1.37E-19	postive
LCK	AL357033.4	0.440467722	4.46E-21	postive
LTA	AL357033.4	0.449420961	5.66E-22	postive
ABCA1	AC244093.4	0.665117167	3.28E-54	postive
FZD5	AC244093.4	0.440369248	4.57E-21	postive
ABCA1	AL162727.2	0.576272248	5.29E-38	postive
FZD5	AL162727.2	0.514820794	2.14E-29	postive
SLC11A2	AL162727.2	0.421960149	2.64E-19	postive

CALCRL	AL008633.1	0.44011782	4.83E-21	postive
FZD5	AL008633.1	0.687790225	2.75E-59	postive
GPC3	AL008633.1	0.542442426	4.79E-33	postive
SLC11A2	AL008633.1	0.46969376	4.17E-24	postive
ABCA1	LINC02256	0.433694138	2.05E-20	postive
CYBB	LINC02256	0.4648195	1.40E-23	postive
IL10RA	LINC02256	0.41030044	3.05E-18	postive
P2RX7	LINC02256	0.415149841	1.12E-18	postive
PIK3R5	LINC02256	0.42721002	8.52E-20	postive
STAB1	LINC02256	0.438775008	6.55E-21	postive
TACR1	LINC02256	0.410256439	3.08E-18	postive
TLR1	LINC02256	0.472103686	2.27E-24	postive
ABCA1	AL139353.2	0.566624528	1.57E-36	postive
FZD5	AL139353.2	0.453320128	2.26E-22	postive
SLC11A2	AL139353.2	0.414455477	1.29E-18	postive
ABCA1	FKBP14-AS1	0.567172111	1.30E-36	postive
ABCA1	ITFG1-AS1	0.505822525	2.81E-28	postive
KCNMB2	ITFG1-AS1	0.400585454	2.18E-17	postive
C3AR1	LINC01914	0.445615384	1.37E-21	postive
CD14	LINC01914	0.438355748	7.20E-21	postive

CMKLR1	LINC01914	0.437955911	7.88E-21	postive
CXCL9	LINC01914	0.542888864	4.15E-33	postive
CXCR6	LINC01914	0.491568065	1.43E-26	postive
CYBB	LINC01914	0.441409265	3.60E-21	postive
IL10RA	LINC01914	0.568432429	8.38E-37	postive
KCNA3	LINC01914	0.41214326	2.09E-18	postive
LCP2	LINC01914	0.513401745	3.23E-29	postive
P2RX7	LINC01914	0.420608408	3.53E-19	postive
PIK3R5	LINC01914	0.53121473	1.60E-31	postive
PTAFR	LINC01914	0.406730399	6.33E-18	postive
SCN1B	LINC01914	0.452743772	2.59E-22	postive
TNFRSF1B	LINC01914	0.466858779	8.45E-24	postive
TNFRSF9	LINC01914	0.53439168	6.01E-32	postive
CALCRL	AC007406.3	0.449060537	6.15E-22	postive
GPC3	AC007406.3	0.402520122	1.48E-17	postive
C3AR1	AC008750.1	0.461770512	2.95E-23	postive
CD48	AC008750.1	0.55673992	4.51E-35	postive
CXCR6	AC008750.1	0.431536043	3.30E-20	postive
EBI3	AC008750.1	0.498772737	2.00E-27	postive
GPR183	AC008750.1	0.438863303	6.42E-21	postive

IL10RA	AC008750.1	0.454150947	1.85E-22	postive
LCK	AC008750.1	0.536358198	3.26E-32	postive
LCP2	AC008750.1	0.560769341	1.16E-35	postive
LTA	AC008750.1	0.437313597	9.11E-21	postive
PIK3R5	AC008750.1	0.41606637	9.21E-19	postive
PTGIR	AC008750.1	0.415985951	9.37E-19	postive
RGS1	AC008750.1	0.472516974	2.05E-24	postive
SLAMF1	AC008750.1	0.451343465	3.60E-22	postive
ABCA1	AP001619.1	0.553761041	1.22E-34	postive
FZD5	AP001619.1	0.479622456	3.33E-25	postive
KCNMB2	AP001619.1	0.411908583	2.19E-18	postive
SLC11A2	AP001619.1	0.40543122	8.23E-18	postive
CXCL10	ETV7-AS1	0.431425821	3.38E-20	postive
CXCR6	ETV7-AS1	0.43557115	1.35E-20	postive
IL15	ETV7-AS1	0.43143651	3.38E-20	postive
IL15RA	ETV7-AS1	0.432472314	2.68E-20	postive
IL18RAP	ETV7-AS1	0.451102692	3.81E-22	postive
IRF1	ETV7-AS1	0.503597138	5.25E-28	postive
NMI	ETV7-AS1	0.430705638	3.97E-20	postive
RTP4	ETV7-AS1	0.423998847	1.71E-19	postive

TAPBP	ETV7-AS1	0.500656807	1.19E-27	postive
APLNR	AC087521.1	0.461712846	3.00E-23	postive
CALCRL	AC087521.1	0.455611639	1.31E-22	postive
CCL2	AC087521.1	0.463907011	1.75E-23	postive
GPR183	AC087521.1	0.546661386	1.24E-33	postive
IL10	AC087521.1	0.425794638	1.16E-19	postive
IL6	AC087521.1	0.4207479	3.43E-19	postive
LIF	AC087521.1	0.403221084	1.29E-17	postive
PDE4B	AC087521.1	0.503244907	5.79E-28	postive
PTGER2	AC087521.1	0.41059159	2.87E-18	postive
PTGIR	AC087521.1	0.415412426	1.06E-18	postive
SCARF1	AC087521.1	0.412335449	2.00E-18	postive
BDKRB1	MIR100HG	0.402818928	1.39E-17	postive
BEST1	MIR100HG	0.454957587	1.53E-22	postive
CCL2	MIR100HG	0.429575052	5.08E-20	postive
EMP3	MIR100HG	0.460763226	3.78E-23	postive
FPR1	MIR100HG	0.425509454	1.23E-19	postive
IL6	MIR100HG	0.455094098	1.48E-22	postive
ITGA5	MIR100HG	0.684038465	2.05E-58	postive
PDE4B	MIR100HG	0.454386462	1.75E-22	postive

PTGER2	MIR100HG	0.451684271	3.32E-22	postive
SCN1B	MIR100HG	0.539006543	1.42E-32	postive
TIMP1	MIR100HG	0.501404046	9.68E-28	postive
TNFAIP6	MIR100HG	0.402013037	1.64E-17	postive
ABCA1	AC008750.4	0.632602834	1.16E-47	postive
FZD5	AC008750.4	0.447646473	8.56E-22	postive
TLR1	AC008750.4	0.400831291	2.07E-17	postive
KCNMB2	STPG3-AS1	0.408792084	4.16E-18	postive
KCNMB2	AC084357.2	0.441217187	3.76E-21	postive
CCL20	LINC02154	0.426465026	1.00E-19	postive
EREG	LINC02154	0.506056166	2.63E-28	postive
HIF1A	LINC02154	0.436392848	1.12E-20	postive
ABCA1	AC008735.1	0.457977229	7.41E-23	postive
ABCA1	AL031600.1	0.424264865	1.61E-19	postive
ABCA1	NR2F2-AS1	0.534248015	6.28E-32	postive
FZD5	NR2F2-AS1	0.535970836	3.68E-32	postive
KCNMB2	NR2F2-AS1	0.420224052	3.83E-19	postive
SLC11A2	NR2F2-AS1	0.468202489	6.05E-24	postive
ABCA1	AL109614.1	0.492327944	1.16E-26	postive
FZD5	AL109614.1	0.419782371	4.21E-19	postive

ABCA1	RASA3-IT1	0.610798773	1.08E-43	postive
CYBB	RASA3-IT1	0.57833181	2.53E-38	postive
TLR1	RASA3-IT1	0.604556857	1.30E-42	postive
ABCA1	AC007684.2	0.601326953	4.61E-42	postive
FZD5	AC007684.2	0.5471575	1.05E-33	postive
KCNMB2	AC007684.2	0.445234744	1.50E-21	postive
SLC11A2	AC007684.2	0.400145113	2.38E-17	postive
ABCA1	GLYCTK-AS1	0.55940399	1.85E-35	postive
PTPRE	AC015660.2	0.411278823	2.49E-18	postive
PIK3R5	AL450326.1	0.442533878	2.79E-21	postive
STAB1	AL450326.1	0.557108849	3.99E-35	postive
TACR1	AL450326.1	0.476449254	7.54E-25	postive
ABCA1	AC007365.1	0.62438418	3.95E-46	postive
FZD5	AC007365.1	0.560062955	1.48E-35	postive
KCNMB2	AC007365.1	0.441023621	3.93E-21	postive
PDE4B	LMF1-AS1	0.417100581	7.41E-19	postive
TNFRSF9	LMF1-AS1	0.401384749	1.86E-17	postive
CMKLR1	LINC01711	0.461698119	3.01E-23	postive
CSF1	LINC01711	0.438040642	7.74E-21	postive
EMP3	LINC01711	0.628543995	6.70E-47	postive

MMP14	LINC01711	0.456633095	1.02E-22	postive
NMUR1	LINC01711	0.594633599	6.07E-41	postive
TIMP1	LINC01711	0.484934742	8.33E-26	postive
ABCA1	AC093799.1	0.430069028	4.56E-20	postive
GABBR1	AC005790.1	0.407169035	5.79E-18	postive
ABCA1	NOP53-AS1	0.424937855	1.39E-19	postive
ABCA1	GNG12-AS1	0.790775814	7.38E-90	postive
BEST1	GNG12-AS1	0.400554194	2.19E-17	postive
CYBB	GNG12-AS1	0.642471497	1.44E-49	postive
KIF1B	GNG12-AS1	0.464522221	1.51E-23	postive
PIK3R5	GNG12-AS1	0.451979714	3.10E-22	postive
STAB1	GNG12-AS1	0.594691603	5.93E-41	postive
TACR1	GNG12-AS1	0.575874162	6.10E-38	postive
TLR1	GNG12-AS1	0.641281697	2.47E-49	postive
ABCA1	ZDHHC20-IT1	0.428787404	6.04E-20	postive
FZD5	ZDHHC20-IT1	0.543677068	3.23E-33	postive
KCNMB2	ZDHHC20-IT1	0.404353571	1.02E-17	postive
SLC11A2	ZDHHC20-IT1	0.426090546	1.09E-19	postive
PTPRE	AC021242.3	0.549559851	4.83E-34	postive
ABCA1	AC139887.1	0.513300702	3.32E-29	postive

FZD5	AC139887.1	0.459419233	5.23E-23	postive
SLC11A2	AC139887.1	0.430860737	3.83E-20	postive
ABCA1	AC110769.2	0.590063412	3.41E-40	postive
FZD5	AC110769.2	0.499549828	1.62E-27	postive
KCNMB2	AC110769.2	0.422973739	2.13E-19	postive
ABCA1	AC011447.3	0.428670603	6.20E-20	postive
FZD5	AC011447.3	0.520299786	4.29E-30	postive
SLC11A2	AC011447.3	0.537163104	2.54E-32	postive
ABCA1	AC138393.2	0.706908774	6.05E-64	postive
KIF1B	AC138393.2	0.42134194	3.02E-19	postive
FPR1	LINC02550	0.400954524	2.02E-17	postive
ABCA1	AL133243.1	0.694915509	5.57E-61	postive
TACR1	AL133243.1	0.433639246	2.07E-20	postive
ABCA1	AC004596.1	0.445416448	1.44E-21	postive
FZD5	AC004596.1	0.420963087	3.27E-19	postive
ABCA1	AL356124.1	0.833049754	5.49E-108	postive
CYBB	AL356124.1	0.546557531	1.28E-33	postive
FZD5	AL356124.1	0.483759506	1.13E-25	postive
STAB1	AL356124.1	0.541374744	6.72E-33	postive
TACR1	AL356124.1	0.565681074	2.17E-36	postive

TLR1	AL356124.1	0.523631603	1.59E-30	postive
CALCRL	AC011472.4	0.564389426	3.39E-36	postive
FZD5	AC011472.4	0.651946917	1.84E-51	postive
GPC3	AC011472.4	0.932322171	4.25E-184	postive
SLC11A2	AC011472.4	0.477471589	5.80E-25	postive
APLNR	AL590226.1	0.600647069	6.00E-42	postive
ITGA5	AL590226.1	0.423373438	1.95E-19	postive
PTGIR	AL590226.1	0.524873852	1.10E-30	postive
SCARF1	AL590226.1	0.457479575	8.35E-23	postive
TIMP1	AL590226.1	0.482332296	1.65E-25	postive
CD14	APCDD1L-DT	0.483566643	1.19E-25	postive
CMKLR1	APCDD1L-DT	0.475841351	8.81E-25	postive
CSF1	APCDD1L-DT	0.422404036	2.40E-19	postive
EMP3	APCDD1L-DT	0.542786715	4.29E-33	postive
SCN1B	APCDD1L-DT	0.53111887	1.65E-31	postive
ABCA1	AC007686.3	0.462664491	2.37E-23	postive
ABCA1	AC068790.3	0.661132017	2.30E-53	postive
FZD5	AC068790.3	0.492339605	1.16E-26	postive
KCNMB2	AC068790.3	0.430463869	4.18E-20	postive
ABCA1	AC104984.5	0.750708351	3.48E-76	postive

CYBB	AC104984.5	0.431931015	3.03E-20	postive
KIF1B	AC104984.5	0.417291042	7.12E-19	postive
TACR1	AC104984.5	0.426003521	1.11E-19	postive
TLR1	AC104984.5	0.43872073	6.63E-21	postive
CD40	U62631.1	0.428996756	5.77E-20	postive
CD48	U62631.1	0.812108312	1.97E-98	postive
EBI3	U62631.1	0.582382763	5.83E-39	postive
GP1BA	U62631.1	0.774205745	7.32E-84	postive
GPR183	U62631.1	0.492300732	1.17E-26	postive
KCNA3	U62631.1	0.425874247	1.14E-19	postive
LCK	U62631.1	0.72674969	3.41E-69	postive
LTA	U62631.1	0.796288521	5.64E-92	postive
SLAMF1	U62631.1	0.656470921	2.17E-52	postive
EBI3	LINC00954	0.458463821	6.59E-23	postive
SLAMF1	LINC00954	0.515315227	1.85E-29	postive
PTPRE	AC083906.3	0.402088318	1.61E-17	postive
CYBB	AC107308.1	0.449489917	5.56E-22	postive
TLR1	AC107308.1	0.465127372	1.30E-23	postive
TLR2	AC107308.1	0.427019189	8.88E-20	postive
ABCA1	FRMD6-AS1	0.508563809	1.29E-28	postive

FZD5	PCAT7	0.448444123	7.11E-22	postive
GPC3	PCAT7	0.479963497	3.05E-25	postive
SLC11A2	PCAT7	0.420805264	3.38E-19	postive
ABCA1	AL590133.1	0.478609258	4.33E-25	postive
ABCA1	SOCAR	0.53045203	2.02E-31	postive
BEST1	SOCAR	0.508263882	1.41E-28	postive
C3AR1	SOCAR	0.482912922	1.42E-25	postive
C5AR1	SOCAR	0.430941005	3.77E-20	postive
CMKLR1	SOCAR	0.442066344	3.10E-21	postive
CXCR6	SOCAR	0.461296268	3.32E-23	postive
CYBB	SOCAR	0.635996132	2.61E-48	postive
FPR1	SOCAR	0.401481822	1.82E-17	postive
GPR183	SOCAR	0.44663523	1.08E-21	postive
IL10RA	SOCAR	0.532763902	9.94E-32	postive
LCP2	SOCAR	0.520807465	3.69E-30	postive
MSR1	SOCAR	0.460989963	3.57E-23	postive
NLRP3	SOCAR	0.476743505	6.99E-25	postive
P2RX7	SOCAR	0.467968652	6.41E-24	postive
PIK3R5	SOCAR	0.526584003	6.56E-31	postive
RGS1	SOCAR	0.454963662	1.53E-22	postive

TLR1	SOCAR	0.618094672	5.49E-45	postive
TNFRSF1B	SOCAR	0.409062413	3.93E-18	postive
ABCA1	AC009716.1	0.66004221	3.90E-53	postive
CYBB	AC009716.1	0.509720068	9.29E-29	postive
KIF1B	AC009716.1	0.457056948	9.25E-23	postive
TLR1	AC009716.1	0.546763031	1.20E-33	postive
C3AR1	AC110995.1	0.468470918	5.66E-24	postive
CD14	AC110995.1	0.511711029	5.25E-29	postive
CMKLR1	AC110995.1	0.516487208	1.32E-29	postive
CSF1	AC110995.1	0.490246345	2.03E-26	postive
CYBB	AC110995.1	0.445292468	1.48E-21	postive
EMP3	AC110995.1	0.426503996	9.94E-20	postive
ADGRE1	AC110995.1	0.423457949	1.92E-19	postive
IL10RA	AC110995.1	0.438606301	6.81E-21	postive
LCP2	AC110995.1	0.401793849	1.71E-17	postive
PIK3R5	AC110995.1	0.459388142	5.27E-23	postive
SCN1B	AC110995.1	0.434798739	1.60E-20	postive
STAB1	AC110995.1	0.408610853	4.31E-18	postive
FZD5	AL354993.2	0.601770142	3.87E-42	postive
KCNMB2	AL354993.2	0.413945375	1.43E-18	postive

SLC11A2	AL354993.2	0.504971251	3.57E-28	postive
SELL	AC119150.1	0.467296217	7.58E-24	postive
CCR7	AL008718.2	0.404030821	1.09E-17	postive
SELL	AC096759.2	0.40480299	9.35E-18	postive
ABCA1	C1QTNF7-AS1	0.669274502	4.15E-55	postive
CYBB	C1QTNF7-AS1	0.675924037	1.42E-56	postive
PIK3R5	C1QTNF7-AS1	0.429180524	5.54E-20	postive
STAB1	C1QTNF7-AS1	0.530705413	1.87E-31	postive
TACR1	C1QTNF7-AS1	0.469305284	4.59E-24	postive
TLR1	C1QTNF7-AS1	0.682586572	4.42E-58	postive
ABCA1	AC004584.1	0.426815309	9.29E-20	postive
ABCA1	MANEA-DT	0.427214894	8.51E-20	postive
ABCA1	AC008543.1	0.443330329	2.32E-21	postive
FZD5	AC008543.1	0.434000911	1.91E-20	postive
KCNMB2	AC008543.1	0.51783923	8.86E-30	postive
FZD5	AC092910.3	0.431103704	3.63E-20	postive
KCNMB2	AC092910.3	0.40326709	1.27E-17	postive
SLC11A2	AC092910.3	0.426149046	1.07E-19	postive
ABCA1	SNHG4	0.559089198	2.05E-35	postive
STAB1	SNHG4	0.41940818	4.55E-19	postive

TACR1	SNHG4	0.414116824	1.38E-18	postive
KIF1B	AL122035.1	0.43055045	4.10E-20	postive
CCL24	AC012625.1	0.73966941	7.22E-73	postive
KCNMB2	AC084864.1	0.501284168	1.00E-27	postive
PTPRE	AC069120.1	0.487979036	3.72E-26	postive
ABCA1	AC007546.1	0.450477024	4.41E-22	postive
SLC11A2	AC007546.1	0.489169085	2.71E-26	postive
P2RY2	AP002761.1	0.443951226	2.01E-21	postive
ITGA5	AC120498.2	0.412861527	1.80E-18	postive
ABCA1	AC090589.3	0.50854283	1.30E-28	postive
ABCA1	NCBP2-AS1	0.524217902	1.34E-30	postive
FZD5	NCBP2-AS1	0.554669583	8.99E-35	postive
SLC11A2	NCBP2-AS1	0.428027405	7.13E-20	postive
ABCA1	AC119800.1	0.456657804	1.02E-22	postive
FZD5	AC119800.1	0.48244587	1.60E-25	postive
KCNMB2	AC119800.1	0.400262375	2.32E-17	postive
BEST1	GORAB-AS1	0.402772147	1.41E-17	postive
C3AR1	GORAB-AS1	0.483212634	1.31E-25	postive
C5AR1	GORAB-AS1	0.479500665	3.44E-25	postive
CLEC5A	GORAB-AS1	0.483612165	1.18E-25	postive

F3	GORAB-AS1	0.412637086	1.88E-18	postive
FPR1	GORAB-AS1	0.462772856	2.31E-23	postive
GPR132	GORAB-AS1	0.408181778	4.71E-18	postive
GPR183	GORAB-AS1	0.407224543	5.72E-18	postive
ICAM1	GORAB-AS1	0.407844646	5.04E-18	postive
IL10RA	GORAB-AS1	0.405534168	8.06E-18	postive
IL15RA	GORAB-AS1	0.401141864	1.95E-17	postive
IL7R	GORAB-AS1	0.479603655	3.35E-25	postive
INHBA	GORAB-AS1	0.496821349	3.43E-27	postive
ITGA5	GORAB-AS1	0.425570948	1.22E-19	postive
LCP2	GORAB-AS1	0.481552397	2.02E-25	postive
MMP14	GORAB-AS1	0.5243425	1.29E-30	postive
OSM	GORAB-AS1	0.49035743	1.97E-26	postive
PLAUR	GORAB-AS1	0.480875591	2.41E-25	postive
PTAFR	GORAB-AS1	0.458360271	6.76E-23	postive
PTGIR	GORAB-AS1	0.537977584	1.96E-32	postive
RGS1	GORAB-AS1	0.511523997	5.54E-29	postive
SPHK1	GORAB-AS1	0.501165357	1.03E-27	postive
TNFAIP6	GORAB-AS1	0.575355054	7.34E-38	postive
ABCA1	TH2LCRR	0.611183761	9.24E-44	postive

FZD5	TH2LCRR	0.473323387	1.67E-24	postive
KCNMB2	TH2LCRR	0.638052021	1.05E-48	postive
BEST1	AL031429.2	0.400747717	2.11E-17	postive
CYBB	AL031429.2	0.521036742	3.45E-30	postive
NLRP3	AL031429.2	0.400483199	2.22E-17	postive
PIK3R5	AL031429.2	0.477244711	6.15E-25	postive
STAB1	AL031429.2	0.505698399	2.91E-28	postive
TACR1	AL031429.2	0.531656569	1.40E-31	postive
TLR1	AL031429.2	0.480165759	2.89E-25	postive
ABCA1	MIRLET7A1HG	0.631370112	1.98E-47	postive
FZD5	MIRLET7A1HG	0.635817446	2.82E-48	postive
KCNMB2	MIRLET7A1HG	0.476544766	7.36E-25	postive
SLC11A2	MIRLET7A1HG	0.440274365	4.67E-21	postive
ABCA1	AL133445.2	0.720644992	1.57E-67	postive
CYBB	AL133445.2	0.562190045	7.18E-36	postive
TACR1	AL133445.2	0.423133487	2.06E-19	postive
TLR1	AL133445.2	0.587262419	9.68E-40	postive
BEST1	AC093010.2	0.411944728	2.17E-18	postive
PTGER2	AC093010.2	0.408906997	4.06E-18	postive
ABCA1	AC005021.1	0.537006735	2.66E-32	postive

FZD5	AC005021.1	0.430845134	3.85E-20	postive
MYC	AL604028.1	-0.409732762	3.43E-18	negative
OSMR	AL604028.1	-0.41856738	5.44E-19	negative
OSMR	SPINT1-AS1	-0.429679139	4.97E-20	negative
ABCA1	CARMN	0.683400845	2.87E-58	postive
CALCRL	CARMN	0.419676019	4.30E-19	postive
FZD5	CARMN	0.552222298	2.02E-34	postive
KCNMB2	CARMN	0.441869381	3.24E-21	postive
TACR1	CARMN	0.415021089	1.15E-18	postive
CD55	BRWD1-AS2	0.402185815	1.58E-17	postive
ABCA1	NUTM2B-AS1	0.548566428	6.68E-34	postive
FZD5	NUTM2B-AS1	0.631668334	1.74E-47	postive
GPC3	NUTM2B-AS1	0.403061673	1.33E-17	postive
KCNMB2	NUTM2B-AS1	0.434289051	1.79E-20	postive
SLC11A2	NUTM2B-AS1	0.434726957	1.63E-20	postive
ABCA1	MKNK1-AS1	0.728358137	1.22E-69	postive
CYBB	MKNK1-AS1	0.538113821	1.88E-32	postive
KIF1B	MKNK1-AS1	0.412287926	2.02E-18	postive
TACR1	MKNK1-AS1	0.404673032	9.60E-18	postive
TLR1	MKNK1-AS1	0.560509956	1.27E-35	postive

ABCA1	AC110792.3	0.524360894	1.28E-30	postive
FZD5	AC110792.3	0.551549141	2.52E-34	postive
KCNMB2	AC110792.3	0.461631494	3.06E-23	postive
ABCA1	SUGT1P4-STRA6LP	0.488356983	3.37E-26	postive
GPC3	AC007953.1	0.424349319	1.58E-19	postive
CHST2	AC018450.1	0.733154463	5.47E-71	postive
KCNMB2	AC012360.1	0.410564199	2.89E-18	postive
ITGA5	MBNL1-AS1	0.69326511	1.39E-60	postive
F3	SFTA1P	0.466279046	9.76E-24	postive
ABCA1	AC073283.1	0.485325826	7.51E-26	postive
FZD5	AC073283.1	0.54318623	3.78E-33	postive
GPC3	AC073283.1	0.405932208	7.44E-18	postive
SLC11A2	AC073283.1	0.432731088	2.54E-20	postive
ABCA1	AC087854.1	0.649789662	5.04E-51	postive
CALCRL	AC087854.1	0.438311706	7.28E-21	postive
CYBB	AC087854.1	0.525320661	9.59E-31	postive
PIK3R5	AC087854.1	0.423988687	1.71E-19	postive
STAB1	AC087854.1	0.408369111	4.53E-18	postive
TACR1	AC087854.1	0.512154505	4.62E-29	postive
TLR1	AC087854.1	0.546035697	1.51E-33	postive

ATP2A2	OGFRP1	0.406550271	6.56E-18	postive
IL1B	LINC01419	0.443437321	2.27E-21	postive
ABCA1	AC023034.1	0.497620396	2.75E-27	postive
FZD5	AC023034.1	0.410455669	2.95E-18	postive
ABCA1	AC010186.3	0.609804416	1.61E-43	postive
FZD5	AC010186.3	0.620259009	2.23E-45	postive
KCNMB2	AC010186.3	0.405891355	7.50E-18	postive
SLC11A2	AC010186.3	0.464018928	1.70E-23	postive
FZD5	AL021878.2	0.465105652	1.30E-23	postive
KCNMB2	AL021878.2	0.426593348	9.74E-20	postive
SLC11A2	AL021878.2	0.473009458	1.81E-24	postive
ABCA1	AP001528.1	0.510008798	8.56E-29	postive
TACR1	AP001528.1	0.403546913	1.20E-17	postive
ABCA1	C2-AS1	0.441969711	3.17E-21	postive
CYBB	C2-AS1	0.43366888	2.06E-20	postive
TLR1	C2-AS1	0.451959485	3.11E-22	postive
ABCA1	AC244093.5	0.741902466	1.59E-73	postive
FZD5	AC244093.5	0.495627682	4.75E-27	postive
KIF1B	AC244093.5	0.42015015	3.89E-19	postive
TACR1	AC244093.5	0.473901252	1.44E-24	postive

ACVR2A	AC004076.2	0.409188616	3.83E-18	postive
ABCA1	AP001432.1	0.639154267	6.41E-49	postive
KIF1B	AP001432.1	0.429547304	5.11E-20	postive
ABCA1	AC073517.1	0.590557389	2.83E-40	postive
FZD5	AC073517.1	0.472686402	1.96E-24	postive
KCNMB2	AC073517.1	0.460891692	3.66E-23	postive
AXL	TTLL11-IT1	0.403224808	1.28E-17	postive
CCL7	TTLL11-IT1	0.42078456	3.40E-19	postive
CXCL10	TTLL11-IT1	0.550292435	3.80E-34	postive
CXCL11	TTLL11-IT1	0.524639125	1.18E-30	postive
F3	TTLL11-IT1	0.445883381	1.29E-21	postive
ICAM1	TTLL11-IT1	0.411596098	2.33E-18	postive
IFITM1	TTLL11-IT1	0.550275768	3.82E-34	postive
IL15	TTLL11-IT1	0.480099279	2.94E-25	postive
IL15RA	TTLL11-IT1	0.40125551	1.90E-17	postive
IL18RAP	TTLL11-IT1	0.41325227	1.66E-18	postive
LAMP3	TTLL11-IT1	0.479744321	3.23E-25	postive
SPHK1	TTLL11-IT1	0.409329453	3.72E-18	postive
FZD5	AL390067.1	0.466697834	8.79E-24	postive
ABCA1	AL606534.1	0.565993691	1.95E-36	postive

CYBB	AL606534.1	0.441094923	3.87E-21	postive
TLR1	AL606534.1	0.493263935	9.02E-27	postive
ABCA1	AC114980.1	0.604430493	1.36E-42	postive
ABCA1	AC112722.1	0.477782829	5.35E-25	postive
FZD5	AC112722.1	0.524020952	1.42E-30	postive
KCNMB2	AC112722.1	0.433152756	2.31E-20	postive
FZD5	LINC02466	0.427948827	7.26E-20	postive
ABCA1	AC092301.1	0.594686459	5.94E-41	postive
ABCA1	AC127024.4	0.65654514	2.09E-52	postive
ABCA1	AC009095.1	0.664694693	4.04E-54	postive
FZD5	AC009095.1	0.455873574	1.23E-22	postive
SLC11A2	AC009095.1	0.418629908	5.37E-19	postive
CALCRL	AC096921.2	0.434467701	1.72E-20	postive
ABCA1	OSMR-AS1	0.419514484	4.45E-19	postive
BEST1	OSMR-AS1	0.417283101	7.13E-19	postive
CYBB	OSMR-AS1	0.515512314	1.75E-29	postive
IL15	OSMR-AS1	0.430939303	3.77E-20	postive
LPAR1	OSMR-AS1	0.43460754	1.67E-20	postive
NAMPT	OSMR-AS1	0.475578245	9.42E-25	postive
OSMR	OSMR-AS1	0.677602787	5.98E-57	postive

P2RX7	OSMR-AS1	0.441311203	3.68E-21	postive
TLR1	OSMR-AS1	0.564659311	3.09E-36	postive
IFNAR1	IL10RB-DT	0.487658959	4.05E-26	postive
IFNGR2	IL10RB-DT	0.411480542	2.39E-18	postive
ABCA1	AC004918.3	0.612955549	4.51E-44	postive
KIF1B	AC004918.3	0.422868899	2.18E-19	postive
ABCA1	AC011405.1	0.655016093	4.33E-52	postive
FZD5	AC011405.1	0.483026964	1.37E-25	postive
FZD5	AC002347.1	0.573543045	1.40E-37	postive
GPC3	AC002347.1	0.428907529	5.88E-20	postive
KCNMB2	AC002347.1	0.421650937	2.83E-19	postive
SGMS2	LINC02604	-0.425985522	1.11E-19	negative
ABCA1	AC004832.4	0.735571624	1.12E-71	postive
CYBB	AC004832.4	0.42108432	3.19E-19	postive
FZD5	AC004832.4	0.48411888	1.03E-25	postive
TACR1	AC004832.4	0.458563369	6.43E-23	postive
TLR1	AC004832.4	0.424818341	1.43E-19	postive
ABCA1	AC115102.1	0.702332343	8.52E-63	postive
STAB1	AC115102.1	0.477936767	5.15E-25	postive
TACR1	AC115102.1	0.507010708	2.01E-28	postive

ABCA1	SDK1-AS1	0.465353224	1.23E-23	postive
CALCRL	SDK1-AS1	0.413156699	1.69E-18	postive
FZD5	SDK1-AS1	0.689068836	1.38E-59	postive
GPC3	SDK1-AS1	0.402987735	1.35E-17	postive
KCNMB2	SDK1-AS1	0.479289175	3.63E-25	postive
SLC11A2	SDK1-AS1	0.408293411	4.60E-18	postive
ABCA1	AC078778.1	0.695263611	4.59E-61	postive
FZD5	AC078778.1	0.588012936	7.33E-40	postive
KCNMB2	AC078778.1	0.446935568	1.01E-21	postive
SLC11A2	AC078778.1	0.409215988	3.81E-18	postive
TACR1	AC078778.1	0.46147345	3.18E-23	postive
OSMR	SENCR	0.41490105	1.18E-18	postive
ABCA1	AC006017.1	0.47581375	8.87E-25	postive
FZD5	AC006017.1	0.674143734	3.54E-56	postive
GPC3	AC006017.1	0.406311174	6.89E-18	postive
KCNMB2	AC006017.1	0.480286331	2.80E-25	postive
SLC11A2	AC006017.1	0.443939698	2.02E-21	postive
AXL	SCAT1	0.441181797	3.79E-21	postive
F3	SCAT1	0.433526145	2.13E-20	postive
HRH1	SCAT1	0.417981825	6.16E-19	postive

INHBA	SCAT1	0.402515593	1.48E-17	postive
OSMR	SCAT1	0.526584366	6.55E-31	postive
PLAUR	SCAT1	0.446897975	1.02E-21	postive
PVR	SCAT1	0.407021392	5.96E-18	postive
SPHK1	SCAT1	0.451872758	3.18E-22	postive
TLR2	SCAT1	0.482444357	1.60E-25	postive
ABCA1	AC012557.1	0.579550558	1.63E-38	postive
FZD5	AC012557.1	0.561037593	1.06E-35	postive
KCNMB2	AC012557.1	0.43934534	5.76E-21	postive
FZD5	AC011389.1	0.437282202	9.18E-21	postive
CD48	RBM38-AS1	0.477776501	5.36E-25	postive
GP1BA	RBM38-AS1	0.573914018	1.22E-37	postive
GPR183	RBM38-AS1	0.435665296	1.32E-20	postive
KCNA3	RBM38-AS1	0.456616508	1.03E-22	postive
LCK	RBM38-AS1	0.460967123	3.59E-23	postive
LTA	RBM38-AS1	0.537584878	2.22E-32	postive
PDE4B	RBM38-AS1	0.413941284	1.44E-18	postive
ABCA1	AC087294.1	0.585800217	1.66E-39	postive
FZD5	AC087294.1	0.569304978	6.19E-37	postive
KCNMB2	AC087294.1	0.48259411	1.54E-25	postive

SLC11A2	AC087294.1	0.426133185	1.08E-19	postive
KCNMB2	AC139100.2	0.421830766	2.72E-19	postive
TLR1	DST-AS1	0.402494968	1.49E-17	postive
PTGER2	NR2F1-AS1	0.412389832	1.98E-18	postive
TIMP1	NR2F1-AS1	0.404288752	1.04E-17	postive
KIF1B	LINC01967	0.435224822	1.46E-20	postive
ABCA1	AL359644.1	0.582356098	5.89E-39	postive
CALCRL	AL359644.1	0.537347851	2.39E-32	postive
FZD5	AL359644.1	0.749334006	9.21E-76	postive
GPC3	AL359644.1	0.653296691	9.76E-52	postive
KCNMB2	AL359644.1	0.443444641	2.26E-21	postive
SLC11A2	AL359644.1	0.481237566	2.19E-25	postive
HPN	AL603839.2	0.415848272	9.64E-19	postive
ABCA1	AC138207.4	0.74886282	1.28E-75	postive
BEST1	AC138207.4	0.419430397	4.53E-19	postive
CYBB	AC138207.4	0.696876526	1.87E-61	postive
IL10RA	AC138207.4	0.456539736	1.05E-22	postive
KIF1B	AC138207.4	0.451778517	3.25E-22	postive
PIK3R5	AC138207.4	0.540491116	8.90E-33	postive
STAB1	AC138207.4	0.689432084	1.13E-59	postive

TACR1	AC138207.4	0.608109557	3.17E-43	postive
TLR1	AC138207.4	0.661043494	2.40E-53	postive
ITGA5	LINC00702	0.732437259	8.74E-71	postive
GABBR1	AC011498.6	0.403994573	1.10E-17	postive
ABCA1	AC099811.1	0.689079057	1.37E-59	postive
KIF1B	AC099811.1	0.404525001	9.89E-18	postive
ABCA1	AC068790.7	0.670756747	1.97E-55	postive
FZD5	AC068790.7	0.46567257	1.13E-23	postive
KCNMB2	AC068790.7	0.4136183	1.54E-18	postive
ABCA1	AC010976.1	0.604550159	1.30E-42	postive
FZD5	AC010976.1	0.583909478	3.33E-39	postive
KCNMB2	AC010976.1	0.46163423	3.05E-23	postive
SLC11A2	AC010976.1	0.439218459	5.93E-21	postive
ABCA1	AL157871.5	0.648041787	1.13E-50	postive
KIF1B	AL157871.5	0.433520203	2.13E-20	postive
OSMR	MCF2L-AS1	-0.441966035	3.17E-21	negative
PLAUR	MCF2L-AS1	-0.404105218	1.08E-17	negative
SGMS2	MCF2L-AS1	-0.43494627	1.55E-20	negative
ABCA1	AC023794.4	0.531511392	1.46E-31	postive
FZD5	AC023794.4	0.625326364	2.65E-46	postive

KCNMB2	AC023794.4	0.497087255	3.18E-27	postive
SLC11A2	AC023794.4	0.441991203	3.15E-21	postive
ABCA1	AC121247.1	0.581332878	8.54E-39	postive
CALCRL	AC121247.1	0.451803925	3.23E-22	postive
FZD5	AC121247.1	0.547991191	8.05E-34	postive
IL1R1	AC121247.1	0.40839338	4.51E-18	postive
TACR1	AC121247.1	0.428458549	6.49E-20	postive
OSMR	PAXIP1-DT	-0.439161202	6.00E-21	negative
TLR2	PAXIP1-DT	-0.407580103	5.32E-18	negative
ABCA1	LINC02595	0.498179215	2.36E-27	postive
CYBB	LINC02595	0.491922313	1.30E-26	postive
TLR1	LINC02595	0.505972349	2.69E-28	postive
ABCA1	ENTPD1-AS1	0.785367495	7.65E-88	postive
BEST1	ENTPD1-AS1	0.402157815	1.59E-17	postive
CYBB	ENTPD1-AS1	0.591127085	2.29E-40	postive
KIF1B	ENTPD1-AS1	0.529503053	2.70E-31	postive
PIK3R5	ENTPD1-AS1	0.404732694	9.48E-18	postive
STAB1	ENTPD1-AS1	0.475464798	9.69E-25	postive
TACR1	ENTPD1-AS1	0.51268347	3.97E-29	postive
TLR1	ENTPD1-AS1	0.608543157	2.67E-43	postive

SELL	AC072039.2	0.461001073	3.56E-23	postive
FZD5	AP000873.1	0.44334842	2.31E-21	postive
KCNMB2	MIR600HG	0.456574682	1.04E-22	postive
FZD5	AL139397.1	0.558643118	2.38E-35	postive
SLC11A2	AL139397.1	0.404389874	1.02E-17	postive
BEST1	AC015819.1	0.460230696	4.30E-23	postive
C3AR1	AC015819.1	0.522013199	2.58E-30	postive
C5AR1	AC015819.1	0.439913106	5.06E-21	postive
CD14	AC015819.1	0.413535115	1.56E-18	postive
CD48	AC015819.1	0.628383232	7.18E-47	postive
CMKLR1	AC015819.1	0.41850145	5.52E-19	postive
CXCL9	AC015819.1	0.489145892	2.73E-26	postive
CXCR6	AC015819.1	0.551146832	2.87E-34	postive
FPR1	AC015819.1	0.482355023	1.64E-25	postive
GP1BA	AC015819.1	0.485157413	7.85E-26	postive
GPR132	AC015819.1	0.565918573	2.00E-36	postive
GPR183	AC015819.1	0.515374503	1.82E-29	postive
IL10RA	AC015819.1	0.619380419	3.22E-45	postive
IL18RAP	AC015819.1	0.525687729	8.59E-31	postive
IL2RB	AC015819.1	0.463915243	1.75E-23	postive

IL7R	AC015819.1	0.567071497	1.34E-36	postive
KCNA3	AC015819.1	0.542221965	5.14E-33	postive
LCK	AC015819.1	0.722193096	6.01E-68	postive
LCP2	AC015819.1	0.635729391	2.93E-48	postive
LTA	AC015819.1	0.717193125	1.31E-66	postive
P2RX7	AC015819.1	0.474769109	1.16E-24	postive
PDE4B	AC015819.1	0.461831742	2.91E-23	postive
PIK3R5	AC015819.1	0.52939682	2.79E-31	postive
PTAFR	AC015819.1	0.447735117	8.38E-22	postive
PTGIR	AC015819.1	0.4867713	5.13E-26	postive
RGS1	AC015819.1	0.520504675	4.04E-30	postive
RHOG	AC015819.1	0.507492161	1.75E-28	postive
TIMP1	AC015819.1	0.443750679	2.11E-21	postive
TNFRSF1B	AC015819.1	0.583316108	4.14E-39	postive
TNFRSF9	AC015819.1	0.532357544	1.13E-31	postive
FZD5	LINC00885	0.47138591	2.73E-24	postive
GPC3	LINC00885	0.425279919	1.30E-19	postive
SLC11A2	LINC00885	0.443920771	2.03E-21	postive
FZD5	LINC02886	0.68594476	7.41E-59	postive
GPC3	LINC02886	0.412314027	2.01E-18	postive

KCNMB2	LINC02886	0.50336583	5.60E-28	postive
CALCRL	AC099482.1	0.400622479	2.16E-17	postive
FZD5	AC099482.1	0.711372577	4.36E-65	postive
GPC3	AC099482.1	0.449341986	5.76E-22	postive
KCNMB2	AC099482.1	0.494231839	6.94E-27	postive
SLC11A2	AC099482.1	0.422957524	2.14E-19	postive
EMP3	SLC12A5-AS1	0.466977492	8.20E-24	postive
NMUR1	SLC12A5-AS1	0.835044584	5.76E-109	postive
TIMP1	SLC12A5-AS1	0.416376004	8.63E-19	postive
ABCA1	KCNQ1OT1	0.491237026	1.56E-26	postive
ABCA1	AC112493.1	0.401642482	1.76E-17	postive
FZD5	AC112493.1	0.634735832	4.54E-48	postive
GPC3	AC112493.1	0.465382197	1.22E-23	postive
KCNMB2	AC112493.1	0.414695213	1.23E-18	postive
SLC11A2	AC112493.1	0.407142379	5.82E-18	postive
FZD5	AC008676.1	0.541668196	6.13E-33	postive
ABCA1	AC034102.8	0.447709139	8.43E-22	postive
FZD5	AC034102.8	0.711663673	3.67E-65	postive
GPC3	AC034102.8	0.441499591	3.53E-21	postive
KCNMB2	AC034102.8	0.490953053	1.68E-26	postive

SLC11A2	AC034102.8	0.473172742	1.74E-24	postive
FZD5	LINC01355	0.482828648	1.45E-25	postive
KCNMB2	LINC01355	0.402206622	1.58E-17	postive
SLC11A2	LINC01355	0.405562416	8.02E-18	postive
ABCA1	GHRLOS	0.462652894	2.38E-23	postive
FZD5	GHRLOS	0.500064703	1.40E-27	postive
RAF1	GHRLOS	0.415614364	1.01E-18	postive
ABCA1	AL353801.3	0.499425519	1.67E-27	postive
FZD5	AL353801.3	0.495396516	5.05E-27	postive
TACR1	AL353801.3	0.429486333	5.18E-20	postive
ITGA5	LINC01081	0.538842828	1.50E-32	postive
CD48	LINC02384	0.42860319	6.29E-20	postive
CXCR6	LINC02384	0.447238344	9.41E-22	postive
KCNA3	LINC02384	0.433931542	1.94E-20	postive
LCK	LINC02384	0.418183643	5.90E-19	postive
LTA	LINC02384	0.451081769	3.83E-22	postive
PTAFR	LINC02384	0.419942025	4.07E-19	postive
TNFRSF1B	LINC02384	0.412843586	1.80E-18	postive
OSMR	AC108860.2	-0.400099901	2.40E-17	negative
RHOG	AC108860.2	-0.402321753	1.54E-17	negative

ITGA5	MIR1-1HG-AS1	0.579020539	1.97E-38	postive
ABCA1	AC092794.2	0.547866066	8.38E-34	postive
FZD5	AC092794.2	0.459107363	5.64E-23	postive
KCNMB2	AC092794.2	0.431803543	3.11E-20	postive
ABCA1	AC103746.1	0.407907323	4.98E-18	postive
CALCRL	AC103746.1	0.402680422	1.43E-17	postive
FZD5	AC103746.1	0.679424535	2.32E-57	postive
GPC3	AC103746.1	0.438422327	7.10E-21	postive
KCNMB2	AC103746.1	0.482430157	1.61E-25	postive
SLC11A2	AC103746.1	0.440963379	3.99E-21	postive
ABCA1	AC008966.2	0.512938943	3.69E-29	postive
ABCA1	AC131934.1	0.718837521	4.79E-67	postive
FZD5	AC131934.1	0.421447217	2.95E-19	postive
TACR1	AC131934.1	0.418803523	5.18E-19	postive
ABCA1	AC078852.2	0.568647304	7.78E-37	postive
FZD5	AC078852.2	0.567427195	1.19E-36	postive
KCNMB2	AC078852.2	0.450554318	4.33E-22	postive
ABCA1	AC026356.1	0.432979938	2.40E-20	postive
BEST1	AC026356.1	0.433699238	2.05E-20	postive
CYBB	AC026356.1	0.474042977	1.39E-24	postive

TLR1	AC026356.1	0.50893316	1.16E-28	postive
ABCA1	AL359220.1	0.462818283	2.29E-23	postive
FZD5	AL359220.1	0.512554911	4.12E-29	postive
SLC11A2	AL359220.1	0.494817195	5.92E-27	postive
ABCA1	AC006059.1	0.725900354	5.85E-69	postive
FZD5	AC006059.1	0.403183787	1.29E-17	postive
KIF1B	AC006059.1	0.404429762	1.01E-17	postive
TACR1	AC006059.1	0.400971878	2.02E-17	postive
ABCA1	AC040934.1	0.536998164	2.67E-32	postive
FZD5	AC040934.1	0.470254515	3.62E-24	postive
KCNMB2	AC040934.1	0.41793384	6.22E-19	postive
SLC11A2	AC040934.1	0.412922297	1.77E-18	postive
FZD5	SEPTIN7-DT	0.444627862	1.72E-21	postive
ABCA1	AC012568.1	0.678046205	4.75E-57	postive
CYBB	AC012568.1	0.413009281	1.74E-18	postive
TLR1	AC012568.1	0.43266898	2.57E-20	postive
CCL7	PROSER2-AS1	0.562502038	6.46E-36	postive
RAF1	PROSER2-AS1	0.487680145	4.03E-26	postive
ABCA1	AC037487.2	0.813885605	3.40E-99	postive
CYBB	AC037487.2	0.551460232	2.59E-34	postive

KIF1B	AC037487.2	0.42792598	7.29E-20	postive
STAB1	AC037487.2	0.482649625	1.52E-25	postive
TACR1	AC037487.2	0.523694081	1.56E-30	postive
TLR1	AC037487.2	0.549248259	5.35E-34	postive
STAB1	AC115618.1	0.454191007	1.83E-22	postive
KCNMB2	AC007541.1	0.430186488	4.45E-20	postive
ABCA1	AL080317.1	0.489659064	2.38E-26	postive
FZD5	AL080317.1	0.693630917	1.14E-60	postive
GPC3	AL080317.1	0.421089105	3.19E-19	postive
KCNMB2	AL080317.1	0.514658176	2.24E-29	postive
SLC11A2	AL080317.1	0.451114421	3.80E-22	postive
ABCA1	AC007314.1	0.612977736	4.47E-44	postive
ABCA1	SLFNL1-AS1	0.444898555	1.62E-21	postive
CD48	AL133467.1	0.67266579	7.51E-56	postive
GP1BA	AL133467.1	0.790982518	6.16E-90	postive
GPR132	AL133467.1	0.429463454	5.21E-20	postive
GPR183	AL133467.1	0.444919124	1.61E-21	postive
IL10RA	AL133467.1	0.475958444	8.55E-25	postive
KCNA3	AL133467.1	0.538446316	1.70E-32	postive
LCK	AL133467.1	0.704724377	2.15E-63	postive

LTA	AL133467.1	0.829500115	2.82E-106	postive
PTGIR	AL133467.1	0.407786961	5.10E-18	postive
RGS1	AL133467.1	0.441787729	3.30E-21	postive
TNFRSF1B	AL133467.1	0.434198055	1.83E-20	postive
TNFRSF9	AL133467.1	0.493224462	9.12E-27	postive
ABCA1	AC093535.1	0.488693444	3.08E-26	postive
FZD5	AC093535.1	0.622345245	9.32E-46	postive
KCNMB2	AC093535.1	0.473445156	1.62E-24	postive
SLC11A2	AC093535.1	0.440518089	4.41E-21	postive
FZD5	CASC20	0.586941869	1.09E-39	postive
GPC3	CASC20	0.472049654	2.31E-24	postive
ABCA1	SH3RF3-AS1	0.600479734	6.40E-42	postive
BEST1	SH3RF3-AS1	0.438309976	7.28E-21	postive
CMKLR1	SH3RF3-AS1	0.525325934	9.58E-31	postive
CSF1	SH3RF3-AS1	0.497737435	2.66E-27	postive
CYBB	SH3RF3-AS1	0.638870717	7.28E-49	postive
IL10RA	SH3RF3-AS1	0.534402412	5.99E-32	postive
ITGB3	SH3RF3-AS1	0.43665353	1.06E-20	postive
MEFV	SH3RF3-AS1	0.430274433	4.36E-20	postive
NLRP3	SH3RF3-AS1	0.580648871	1.09E-38	postive

NMUR1	SH3RF3-AS1	0.428343396	6.66E-20	postive
PIK3R5	SH3RF3-AS1	0.64666947	2.13E-50	postive
SCARF1	SH3RF3-AS1	0.50582354	2.81E-28	postive
STAB1	SH3RF3-AS1	0.782955963	5.80E-87	postive
TACR1	SH3RF3-AS1	0.62774995	9.42E-47	postive
TLR1	SH3RF3-AS1	0.562041807	7.55E-36	postive
ABCA1	RUFY1-AS1	0.57744992	3.47E-38	postive
FZD5	RUFY1-AS1	0.415488526	1.04E-18	postive
ABCA1	AL138831.2	0.492488179	1.11E-26	postive
ABCA1	AL049539.1	0.538495319	1.67E-32	postive
NFKBIA	AC099342.1	0.424585988	1.50E-19	postive
SELE	AC099342.1	0.486778154	5.12E-26	postive
ABCA1	AP000692.1	0.456242775	1.12E-22	postive
SLC11A2	AP000692.1	0.414222427	1.35E-18	postive
ABCA1	RC3H1-IT1	0.628021576	8.38E-47	postive
ABCA1	AC087276.2	0.620290797	2.20E-45	postive
ABCA1	AC018752.1	0.678095828	4.63E-57	postive
CALCRL	AC018752.1	0.419523958	4.44E-19	postive
FZD5	AC018752.1	0.464764308	1.42E-23	postive
KIF1B	AC018752.1	0.487371073	4.37E-26	postive

TLR1	AC018752.1	0.443069881	2.46E-21	postive
ITGA5	HAND2-AS1	0.650712053	3.28E-51	postive
ABCA1	RABGAP1L-AS1	0.432484812	2.68E-20	postive
KCNMB2	RPP38-DT	0.539701481	1.14E-32	postive
FZD5	AC093382.1	0.580598985	1.11E-38	postive
KCNMB2	AC093382.1	0.48893802	2.88E-26	postive
AXL	GAS6-DT	0.591391067	2.07E-40	postive
ICAM1	GAS6-DT	0.400240696	2.33E-17	postive
ITGA5	GAS6-DT	0.48884907	2.95E-26	postive
ABCA1	AL133371.2	0.47884551	4.07E-25	postive
APLNR	AL133371.2	0.414219175	1.36E-18	postive
BEST1	AL133371.2	0.60495903	1.11E-42	postive
C3AR1	AL133371.2	0.633888226	6.59E-48	postive
C5AR1	AL133371.2	0.548160888	7.62E-34	postive
CD14	AL133371.2	0.63727732	1.48E-48	postive
CD48	AL133371.2	0.402242314	1.56E-17	postive
CMKLR1	AL133371.2	0.705713213	1.21E-63	postive
CSF1	AL133371.2	0.644486455	5.78E-50	postive
CYBB	AL133371.2	0.824004772	1.05E-103	postive
EMP3	AL133371.2	0.513577714	3.07E-29	postive

ADGRE1	AL133371.2	0.451794529	3.24E-22	postive
FPR1	AL133371.2	0.534781226	5.33E-32	postive
GPR132	AL133371.2	0.452546434	2.71E-22	postive
GPR183	AL133371.2	0.535540158	4.21E-32	postive
IL10	AL133371.2	0.43894248	6.31E-21	postive
IL10RA	AL133371.2	0.786323879	3.40E-88	postive
IL7R	AL133371.2	0.442012978	3.14E-21	postive
KCNA3	AL133371.2	0.491020059	1.65E-26	postive
LCK	AL133371.2	0.454898458	1.55E-22	postive
LCP2	AL133371.2	0.649786222	5.04E-51	postive
LTA	AL133371.2	0.446400428	1.14E-21	postive
MSR1	AL133371.2	0.585784567	1.67E-39	postive
NLRP3	AL133371.2	0.574788632	8.97E-38	postive
P2RX7	AL133371.2	0.586684002	1.20E-39	postive
PDE4B	AL133371.2	0.467501718	7.20E-24	postive
PIK3R5	AL133371.2	0.77820593	2.91E-85	postive
PTGER2	AL133371.2	0.401332514	1.88E-17	postive
RGS1	AL133371.2	0.489655527	2.38E-26	postive
RHOG	AL133371.2	0.436966636	9.85E-21	postive
SCARF1	AL133371.2	0.402142944	1.60E-17	postive

SCN1B	AL133371.2	0.532384638	1.12E-31	postive
STAB1	AL133371.2	0.671446508	1.39E-55	postive
TACR1	AL133371.2	0.580199577	1.29E-38	postive
TIMP1	AL133371.2	0.420069992	3.96E-19	postive
TLR1	AL133371.2	0.711306066	4.54E-65	postive
TNFRSF1B	AL133371.2	0.588330021	6.51E-40	postive
TNFRSF9	AL133371.2	0.445901177	1.28E-21	postive
ABCA1	FIRRE	0.527109577	5.59E-31	postive
FZD5	FIRRE	0.445407984	1.44E-21	postive
SLC11A2	FIRRE	0.465574651	1.16E-23	postive
GABBR1	AC068620.2	0.408683337	4.25E-18	postive
BEST1	MIR223HG	0.537345428	2.39E-32	postive
C3AR1	MIR223HG	0.428090817	7.03E-20	postive
C5AR1	MIR223HG	0.470064146	3.80E-24	postive
CD14	MIR223HG	0.455608674	1.31E-22	postive
CMKLR1	MIR223HG	0.436139208	1.19E-20	postive
CSF1	MIR223HG	0.424932664	1.40E-19	postive
CYBB	MIR223HG	0.435995336	1.23E-20	postive
FPR1	MIR223HG	0.712464557	2.27E-65	postive
GPR183	MIR223HG	0.445593099	1.38E-21	postive

IL10RA	MIR223HG	0.465551144	1.17E-23	postive
IL18RAP	MIR223HG	0.422325658	2.45E-19	postive
ITGA5	MIR223HG	0.402224123	1.57E-17	postive
LCP2	MIR223HG	0.463511268	1.93E-23	postive
NLRP3	MIR223HG	0.430277862	4.36E-20	postive
PDE4B	MIR223HG	0.425816404	1.15E-19	postive
PIK3R5	MIR223HG	0.510705005	7.01E-29	postive
PROK2	MIR223HG	0.464503462	1.51E-23	postive
SCN1B	MIR223HG	0.439787416	5.21E-21	postive
TNFAIP6	MIR223HG	0.403883709	1.12E-17	postive
TNFRSF1B	MIR223HG	0.423154245	2.05E-19	postive
KCNMB2	AC087742.1	0.435161973	1.48E-20	postive
FZD5	AC073957.3	0.43946429	5.61E-21	postive
SLC11A2	AC073957.3	0.422644719	2.28E-19	postive
BST2	U62317.1	0.411891978	2.20E-18	postive
CXCL10	U62317.1	0.476593945	7.26E-25	postive
CXCL11	U62317.1	0.427379682	8.21E-20	postive
IFITM1	U62317.1	0.490648023	1.82E-26	postive
IL15	U62317.1	0.496177517	4.08E-27	postive
IL15RA	U62317.1	0.520638091	3.88E-30	postive

IL18RAP	U62317.1	0.514213257	2.55E-29	postive
IRF1	U62317.1	0.500100708	1.39E-27	postive
LAMP3	U62317.1	0.411921048	2.18E-18	postive
NMI	U62317.1	0.528390585	3.79E-31	postive
TAPBP	U62317.1	0.474213707	1.33E-24	postive
DCBLD2	LINC00973	0.776461263	1.20E-84	postive
ABCA1	AC007285.1	0.51490964	2.08E-29	postive
ABCA1	AL450998.2	0.690272644	7.16E-60	postive
KIF1B	AL450998.2	0.440797913	4.14E-21	postive
ABCA1	AC069542.1	0.666188406	1.93E-54	postive
ABCA1	AL121652.1	0.515767442	1.62E-29	postive
CALCRL	AL121652.1	0.407474764	5.44E-18	postive
FZD5	AL121652.1	0.69001026	8.26E-60	postive
GPC3	AL121652.1	0.412507079	1.93E-18	postive
KCNMB2	AL121652.1	0.501698552	8.92E-28	postive
SLC11A2	AL121652.1	0.419007008	4.96E-19	postive
RHOG	AC107068.1	-0.425111554	1.34E-19	negative
ABCA1	AC078795.1	0.644897804	4.79E-50	postive
FZD5	AC078795.1	0.467035465	8.09E-24	postive
SLC11A2	AC078795.1	0.406538461	6.58E-18	postive

ABCA1	AC008434.1	0.551759401	2.35E-34	postive
GPR183	AC107959.1	0.413197757	1.68E-18	postive
IL10RA	AC107959.1	0.445991724	1.26E-21	postive
KCNA3	AC107959.1	0.432061289	2.94E-20	postive
LCP2	AC107959.1	0.418436643	5.59E-19	postive
PDE4B	AC107959.1	0.419255289	4.70E-19	postive
TNFRSF1B	AC107959.1	0.404577227	9.78E-18	postive
ABCA1	AL450344.3	0.473665297	1.53E-24	postive
FZD5	AL450344.3	0.675660178	1.63E-56	postive
KCNMB2	AL450344.3	0.511324479	5.87E-29	postive
SLC11A2	AL450344.3	0.419452576	4.51E-19	postive
ITGA5	AC053503.3	0.674637261	2.75E-56	postive
ABCA1	AC026782.2	0.455112127	1.47E-22	postive
FZD5	AC026782.2	0.486318447	5.78E-26	postive
KCNMB2	AC026782.2	0.428829982	5.99E-20	postive
FZD5	U47924.1	0.579379385	1.73E-38	postive
GPC3	U47924.1	0.405224867	8.58E-18	postive
KCNMB2	U47924.1	0.425045641	1.36E-19	postive
ABCA1	H3-3A-DT	0.511545459	5.51E-29	postive
FZD5	H3-3A-DT	0.534020026	6.74E-32	postive

KCNMB2	H3-3A-DT	0.636681504	1.93E-48	postive

Supplementary Table 4. The profile of significantly differently infiltrated immune cells between risk groups in different platforms.

Immune	Cor	*P*-value
T cell CD8+_TIMER	0.397650287	1.87E-16
Neutrophil_TIMER	0.423845326	1.07E-18
Macrophage_TIMER	0.239231087	1.47E-06
Myeloid dendritic cell_TIMER	0.419611229	2.55E-18
B cell plasma_CIBERSORT	-0.242142903	1.08E-06
T cell CD4+ naive_CIBERSORT	-0.162159513	0.001202905
T cell CD4+ memory activated_CIBERSORT	0.191584345	0.000125044
T cell follicular helper_CIBERSORT	-0.217421433	1.27E-05
T cell regulatory (Tregs)_CIBERSORT	-0.271253343	4.16E-08
NK cell resting_CIBERSORT	-0.135786237	0.00680756
Macrophage M1_CIBERSORT	0.231465916	3.24E-06
Macrophage M2_CIBERSORT	0.216728377	1.36E-05
Myeloid dendritic cell activated_CIBERSORT	-0.173455627	0.00052605
Neutrophil_CIBERSORT	0.178394528	0.000360484
T cell CD8+_CIBERSORT-ABS	0.159086932	0.001493043

T cell CD4+ naive_CIBERSORT-ABS	-0.165473695	0.000948782
T cell CD4+ memory resting_CIBERSORT-ABS	0.223681149	6.98E-06
T cell CD4+ memory activated_CIBERSORT-ABS	0.203932929	4.34E-05
NK cell activated_CIBERSORT-ABS	0.250064926	4.64E-07
Macrophage M0_CIBERSORT-ABS	0.148731638	0.003008341
Macrophage M1_CIBERSORT-ABS	0.277403946	1.98E-08
Macrophage M2_CIBERSORT-ABS	0.35592944	2.86E-13
Neutrophil_CIBERSORT-ABS	0.202133787	5.09E-05
Macrophage M1_QUANTISEQ	0.40297682	6.80E-17
Macrophage M2_QUANTISEQ	0.225788264	5.69E-06
NK cell_QUANTISEQ	-0.208447696	2.90E-05
T cell CD8+_QUANTISEQ	0.186744725	0.000185967
T cell regulatory (Tregs)_QUANTISEQ	0.26919491	5.31E-08
Myeloid dendritic cell_QUANTISEQ	-0.261148059	1.35E-07
uncharacterized cell_QUANTISEQ	-0.238691239	1.55E-06
T cell CD8+_MCPCOUNTER	0.181532888	0.000282047
cytotoxicity score_MCPCOUNTER	0.299643229	1.17E-09
NK cell_MCPCOUNTER	0.229145726	4.09E-06
Monocyte_MCPCOUNTER	0.353306083	4.38E-13
Macrophage/Monocyte_MCPCOUNTER	0.353306083	4.38E-13

Myeloid dendritic cell_MCPCOUNTER	0.255354966	2.59E-07
Endothelial cell_MCPCOUNTER	0.207295804	3.22E-05
Cancer associated fibroblast_MCPCOUNTER	0.398294554	1.66E-16
Myeloid dendritic cell activated_XCELL	0.329232051	1.83E-11
T cell CD4+ memory_XCELL	0.163633209	0.001083026
T cell CD4+ central memory_XCELL	-0.239117043	1.48E-06
T cell CD8+ central memory_XCELL	0.175900721	0.000436826
Common myeloid progenitor_XCELL	0.149256142	0.002906422
Myeloid dendritic cell_XCELL	0.24546232	7.61E-07
Endothelial cell_XCELL	0.169524039	0.000705659
Cancer associated fibroblast_XCELL	0.273285958	3.26E-08
Granulocyte-monocyte progenitor_XCELL	0.228612797	4.31E-06
Macrophage_XCELL	0.360224727	1.41E-13
Macrophage M1_XCELL	0.378354033	6.34E-15
Macrophage M2_XCELL	0.22820017	4.49E-06
B cell memory_XCELL	0.156350113	0.00180418
Monocyte_XCELL	0.406496389	3.45E-17
Plasmacytoid dendritic cell_XCELL	0.268067956	6.06E-08
B cell plasma_XCELL	-0.175067067	0.000465531
T cell CD4+ Th2_XCELL	0.302691986	7.78E-10

immune score_XCELL	0.284769297	7.98E-09
stroma score_XCELL	0.257430883	2.05E-07
microenvironment score_XCELL	0.345769888	1.46E-12
Cancer associated fibroblast_EPIC	0.307635183	3.98E-10
T cell CD4+_EPIC	-0.234311824	2.43E-06
Macrophage_EPIC	0.314680933	1.50E-10
NK cell_EPIC	0.270877897	4.35E-08
uncharacterized cell_EPIC	-0.259407418	1.64E-07

Supplementary Table 5. The profile of significantly differently infiltrated immune cells among clusters in different platforms.

Immune	*P*-value
T cell CD8+_TIMER	1.02E-05
Neutrophil_TIMER	2.04E-05
Myeloid dendritic cell_TIMER	2.66E-08
B cell plasma_CIBERSORT	1.70E-05
T cell CD4+ naive_CIBERSORT	0.003417992
T cell CD4+ memory activated_CIBERSORT	0.012030799
T cell follicular helper_CIBERSORT	0.009357812
T cell regulatory (Tregs)_CIBERSORT	0.005696105

T cell gamma delta_CIBERSORT	0.031177748
Macrophage M0_CIBERSORT	0.010468014
Macrophage M1_CIBERSORT	0.002906425
Macrophage M2_CIBERSORT	0.003004212
Myeloid dendritic cell activated_CIBERSORT	0.002385278
T cell CD4+ naive_CIBERSORT-ABS	0.003533924
T cell CD4+ memory activated_CIBERSORT-ABS	0.008282715
T cell regulatory (Tregs)_CIBERSORT-ABS	0.029780272
T cell gamma delta_CIBERSORT-ABS	0.02468908
NK cell activated_CIBERSORT-ABS	0.023161479
Macrophage M0_CIBERSORT-ABS	0.001231506
Macrophage M1_CIBERSORT-ABS	0.00059383
Macrophage M2_CIBERSORT-ABS	2.20E-05
Myeloid dendritic cell resting_CIBERSORT-ABS	0.030680208
Myeloid dendritic cell activated_CIBERSORT-ABS	0.030413047
Macrophage M1_QUANTISEQ	1.68E-08
Macrophage M2_QUANTISEQ	0.001124867
Neutrophil_QUANTISEQ	0.033801257
NK cell_QUANTISEQ	0.000268715
Myeloid dendritic cell_QUANTISEQ	2.88E-09

uncharacterized cell_QUANTISEQ	0.020050322
cytotoxicity score_MCPCOUNTER	0.000174046
NK cell_MCPCOUNTER	0.008567626
Monocyte_MCPCOUNTER	4.01E-05
Macrophage/Monocyte_MCPCOUNTER	4.01E-05
Myeloid dendritic cell_MCPCOUNTER	0.001211173
Neutrophil_MCPCOUNTER	0.00258763
Cancer associated fibroblast_MCPCOUNTER	5.13E-08
Myeloid dendritic cell activated_XCELL	6.12E-10
T cell CD4+ central memory_XCELL	6.79E-05
Myeloid dendritic cell_XCELL	7.49E-07
Endothelial cell_XCELL	0.013097694
Eosinophil_XCELL	0.010341806
Cancer associated fibroblast_XCELL	0.004260862
Hematopoietic stem cell_XCELL	0.010539895
Macrophage_XCELL	3.41E-11
Macrophage M1_XCELL	4.71E-13
Macrophage M2_XCELL	9.47E-07
B cell memory_XCELL	0.03679384
Monocyte_XCELL	4.22E-10

Plasmacytoid dendritic cell_XCELL	1.56E-06
T cell CD4+ Th1_XCELL	0.000416016
T cell CD4+ Th2_XCELL	1.56E-07
immune score_XCELL	1.46E-05
stroma score_XCELL	3.34E-05
microenvironment score_XCELL	1.07E-07
Cancer associated fibroblast_EPIC	8.20E-06
T cell CD4+_EPIC	3.14E-06
T cell CD8+_EPIC	0.020467514
Macrophage_EPIC	2.02E-09
NK cell_EPIC	8.80E-08
uncharacterized cell_EPIC	0.045567615
